# Identification and correction of abnormal, incomplete and mispredicted proteins in public databases

**DOI:** 10.1186/1471-2105-9-353

**Published:** 2008-08-27

**Authors:** Alinda Nagy, Hédi Hegyi, Krisztina Farkas, Hedvig Tordai, Evelin Kozma, László Bányai, László Patthy

**Affiliations:** 1 Institute of Enzymology, Biological Research Center, Hungarian Academy of Sciences, H-1113 Budapest, Hungary

## Abstract

**Background:**

Despite significant improvements in computational annotation of genomes, sequences of abnormal, incomplete or incorrectly predicted genes and proteins remain abundant in public databases. Since the majority of incomplete, abnormal or mispredicted entries are not annotated as such, these errors seriously affect the reliability of these databases. Here we describe the MisPred approach that may provide an efficient means for the quality control of databases. The current version of the MisPred approach uses five distinct routines for identifying abnormal, incomplete or mispredicted entries based on the principle that a sequence is likely to be incorrect if some of its features conflict with our current knowledge about protein-coding genes and proteins: (i) conflict between the predicted subcellular localization of proteins and the absence of the corresponding sequence signals; (ii) presence of extracellular and cytoplasmic domains and the absence of transmembrane segments; (iii) co-occurrence of extracellular and nuclear domains; (iv) violation of domain integrity; (v) chimeras encoded by two or more genes located on different chromosomes.

**Results:**

Analyses of predicted EnsEMBL protein sequences of nine deuterostome (*Homo sapiens, Mus musculus, Rattus norvegicus, Monodelphis domestica, Gallus gallus, Xenopus tropicalis, Fugu rubripes, Danio rerio *and *Ciona intestinalis*) and two protostome species (*Caenorhabditis elegans *and *Drosophila melanogaster*) have revealed that the absence of expected signal peptides and violation of domain integrity account for the majority of mispredictions. Analyses of sequences predicted by NCBI's GNOMON annotation pipeline show that the rates of mispredictions are comparable to those of EnsEMBL. Interestingly, even the manually curated UniProtKB/Swiss-Prot dataset is contaminated with mispredicted or abnormal proteins, although to a much lesser extent than UniProtKB/TrEMBL or the EnsEMBL or GNOMON-predicted entries.

**Conclusion:**

MisPred works efficiently in identifying errors in predictions generated by the most reliable gene prediction tools such as the EnsEMBL and NCBI's GNOMON pipelines and also guides the correction of errors. We suggest that application of the MisPred approach will significantly improve the quality of gene predictions and the associated databases.

## Background

Seven years after the first drafts of the human genome were published [1,2] and three years after the completion of sequencing the human genome, the exact number of the protein-coding genes encoded in this genome is still unknown: the most likely estimates range between 20,000–25,000 genes [3,4]. More significantly, recent analyses have shown that the exact genomic structure of human protein-coding genes is correctly predicted for only about 50–60 % of the genes [5,6]. In other words, despite significant advances in computational gene identification, correct prediction of the genomic structure of the protein-coding genes of higher eukaryotes is still a very difficult task.

The main objective of our MisPred project is to develop tools that can be used to identify mispredicted genes/proteins, primarily from Metazoan genomes, in order to inform scientists of the reliability of predictions and to improve the quality of predictions. The key question is: are there signs that may indicate that the predicted structure of a protein-coding gene might be erroneous? The MisPred approach is based on the principle that a protein-coding gene is likely to be mispredicted if some of its features (or features of the protein it encodes) conflict with our current knowledge about protein-coding genes and proteins.

As a proof of principle, in the present work we describe five approaches – based on five dogmas – to identify suspicious proteins that are likely to be abnormal or mispredicted. Accordingly, the current version of MisPred contains five routines, each focusing on a special type of conflict with one of the dogmas.

**(1) Conflict with the dogma that the subcellular localization of extracellular and transmembrane proteins is defined by the presence of appropriate sequence signals**. The validity of this dogma is supported by studies on various disease-causing mutations, indicating that the absence of functional signal peptides prevents the translocation of proteins across the endoplasmic reticulum membrane and the mislocalized protein is rapidly degraded by the proteasome [7-13]. Similarly, the loss of functional transmembrane helices is known to lead to the mislocalization of membrane proteins [14,15]. A major reason for the rapid degradation of mislocalized extracellular proteins is that their extracellular (usually disulphide-bonded) domains are misfolded in the reductive milieu of the cytoplasm and are recognized and degraded by the protein quality control system of the cell [16].

In the current version of MisPred we used domain families the members of which occur only in the extracellular space (e.g. in secreted extracellular proteins and in the extracellular parts of type I, type II, type III single pass or multispanning transmembrane proteins) to identify proteins that are completely or partially extracellular. The justification for this approach is that certain (usually disulphide-rich) domain families are known to have adapted (and are restricted) to the extracellular space: they occur exclusively in extracellular proteins or extracytoplasmic parts of transmembrane proteins [17,18]. Following identification of extracellular or transmembrane proteins containing such extracellular 'marker' domains, we examined whether they have the sequence signals (secretory signal peptides, signal anchors and/or transmembrane helices) that could target these domains to the extracellular space.

According to this dogma, proteins that contain obligatory extracellular domains but lack appropriate sequence signals (signal peptide, signal anchor and transmembrane segments) are considered suspicious (abnormal and nonviable) since their obligatory extracellular domains are not delivered to the extracellular space where they are stable and properly folded.

**(2) Conflict with the dogma that transmembrane proteins containing both cytoplasmic and extracellular parts have at least one transmembrane segment that passes through the cell membrane**. In the current version of MisPred, we used protein domain families the members of which occur exclusively in the extracellular space and exclusively in the cytoplasmic space to identify transmembrane proteins and we asked whether these proteins possess regions that pass through the cell membrane. According to this dogma, proteins that contain both obligatory extracellular and obligatory cytoplasmic domains but lack transmembrane segment(s) separating them are considered erroneous.

**(3) Conflict with the dogma that obligatory extracellular and obligatory nuclear domains do not co-occur in a single, multidomain protein **[17,18]. According to this dogma, proteins that contain both obligatory extracellular and obligatory nuclear domains are considered abnormal and nonviable since they cannot be delivered to a cellular compartment where both types of domains would be correctly folded and fully functional.

**(4) Conflict with the rule that the protein fold is highly conserved in a domain family, therefore the number of amino acid residues in closely related members of a globular domain family usually fall into a relatively narrow range **[19,20]. This phenomenon reflects the fact that the highly cooperative, rapid folding of protein domains is the result of natural selection [18,21], therefore insertion/deletion of larger segments into/from protein domains may yield macromolecules that are unable to rapidly adopt a correctly folded, viable and stable three-dimensional structure. Accordingly, proteins containing domains that consist of a significantly larger or smaller number of residues than closely related members of the same family may be suspected to be abnormal and nonviable.

**(5) Conflict with the dogma that a protein is encoded by exons located on a single chromosome**. According to this dogma, chimeric proteins whose parts are encoded by two or more different genes located on distinct chromosomes are considered abnormal.

In the present work we describe the results of MisPred analyses of various public databases and discuss the values and limitations of the MisPred approach.

## Results and discussion

### Validation of the MisPred approach on the Swiss-Prot section of the UniProtKB

The Swiss-Prot section of UniProtKB is the gold standard of protein databases therefore we have used Swiss-Prot as the benchmark with which to validate the concepts behind the MisPred approach. In view of the high quality of this manually curated database our original expectation was that very few, if any, of the Swiss-Prot entries are truly erroneous therefore it would provide a useful dataset with which to test the specificity of the different MisPred routines.

MisPred analyses of human, mouse, rat, chick, zebrafish, worm and fly Swiss-Prot entries have indeed identified very few Swiss-Prot entries as truly erroneous (see Table 1). The details of the analyses of the Swiss-Prot entries are described in Additional file 1 [see Additional file 1] and the list of the erroneous entries is deposited in Additional file 2 [see Additional file 2]. The majority of these errors could be corrected by targeted search of genomic and EST databases; the protocol used for the correction of errors will be described in another publication (manuscript in preparation).

**Table 1:** MisPred analysis of Swiss-Prot entries

**UniProtKB/Swiss-Prot**											
**Conflict 1**	Number of proteins	Identified as containing an extracellular domain	Percentage	Identified as suspicious by MisPred	Percentage*	False positives	Percentage*	True errors	Percentage*	Annotated as fragment or chimera by UniProt	Identified as abnormal only by MisPred

Homo sapiens	15638	1431	9.2%	15	1.05%	10	0.70%	5	0.35%	4	1
Mus musculus	13186	1198	9.1%	12	1.00%	7	0.58%	5	0.42%	2	3
Rattus norvegicus	6043	599	9.9%	18	3.01%	2	0.33%	16	2.67%	14	2
Gallus gallus	1635	194	11.9%	22	11.34%	3	1.55%	19	9.79%	12	7
Danio rerio	1290	64	5.0%	4	6.25%	3	4.69%	1	1.56%	1	0
Caenorhabditis elegans	2999	119	4.0%	9	7.56%	1	0.84%	8	6.72%	0	8
Drosophila melanogaster	2463	147	6.0%	5	3.40%	3	2.04%	2	1.36%	1	1

**Conflict 2**	Number of proteins	Identified as containing an extra- and an intracellular domain	Percentage	Identified as suspicious by MisPred	Percentage*	False positives	Percentage*	True errors	Percentage*	Annotated as fragment or chimera by UniProt	Identified as abnormal only by MisPred

Homo sapiens	15638	43	0.3%	8	18.6%	8	18.6%	0	0.0%	0	0
Mus musculus	13186	42	0.3%	6	14.3%	6	14.3%	0	0.0%	0	0
Rattus norvegicus	6043	19	0.3%	2	10.5%	2	10.5%	0	0.0%	0	0
Gallus gallus	1635	10	0.6%	1	10.0%	1	10.0%	0	0.0%	0	0
Danio rerio	2999	2	0.1%	0	0.0%	0	0.0%	0	0.0%	0	0
Caenorhabditis elegans	1290	5	0.4%	1	20.0%	1	20.0%	0	0.0%	0	0
Drosophila melanogaster	2463	8	0.3%	1	12.5%	1	12.5%	0	0.0%	0	0

**Conflict 3**	Number of proteins			Identified as suspicious by MisPred	Percentage*	False positives	Percentage*	True errors	Percentage*	Annotated as fragment or chimera by UniProt	Identified as abnormal only by MisPred
			
Homo sapiens	15638			0	0.0%	0	0.0%	0	0.0%	0	0
Mus musculus	13186			0	0.0%	0	0.0%	0	0.0%	0	0
Rattus norvegicus	6043			0	0.0%	0	0.0%	0	0.0%	0	0
Gallus gallus	1635			0	0.0%	0	0.0%	0	0.0%	0	0
Danio rerio	2999			0	0.0%	0	0.0%	0	0.0%	0	0
Caenorhabditis elegans	1290			0	0.0%	0	0.0%	0	0.0%	0	0
Drosophila melanogaster	2463			0	0.0%	0	0.0%	0	0.0%	0	0

**Conflict 4**	Number of proteins	Proteins containing domains suitable for the study of domain integrity	Percentage	Identified as suspicious by MisPred	Percentage*	False positives	Percentage*	True errors	Percentage*	Annotated as fragment or chimera by UniProt	Identified as abnormal only by MisPred

Homo sapiens	15638	6973	44.6%	10	0.14%	6	0.09%	4	0.06%	3	1
Mus musculus	13186	5808	44.0%	3	0.05%	2	0.03%	1	0.02%	1	0
Rattus norvegicus	6043	2756	45.6%	14	0.51%	0	0.00%	14	0.51%	13	1
Gallus gallus	1635	755	46.2%	8	1.06%	0	0.00%	8	1.06%	8	0
Danio rerio	1290	355	27.5%	1	0.28%	0	0.00%	1	0.28%	1	0
Caenorhabditis elegans	2999	1215	40.5%	2	0.16%	0	0.00%	2	0.16%	0	2
Drosophila melanogaster	2463	1203	48.8%	0	0.00%	0	0.00%	0	0.00%	0	0

**Conflict 5**	Number of proteins			Identified as suspicious by MisPred	Percentage*	False positives	Percentage*	True errors	Percentage*	Annotated as fragment or chimera by UniProt	Identified as abnormal only by MisPred
			
Homo sapiens	15638			5	0.03%	3	0.02%	2	0.01%	0	2
Mus musculus	13186			0	0.00%	0	0.00%	0	0.00%	0	0
Rattus norvegicus	6043			5	0.08%	3	0.05%	2	0.03%	0	2
Gallus gallus	1635			0	0.00%	0	0.00%	0	0.00%	0	0
Danio rerio	1290			18	1.40%	18	1.40%	0	0.00%	0	0
Caenorhabditis elegans	2999			0	0.00%	0	0.00%	0	0.00%	0	0
Drosophila melanogaster	2463			0	0.00%	0	0.00%	0	0.00%	0	0

*Values for suspicious, false positive and true positive sequences are expressed as percentage of the proteins relevant for the given conflict.

The majority of truly erroneous sequences were returned for Conflicts 1 and 4, however, these accounted for only 0.03–1.16% and 0.008–0.49% of the sequences of the different species, respectively.

**Figure 1:**
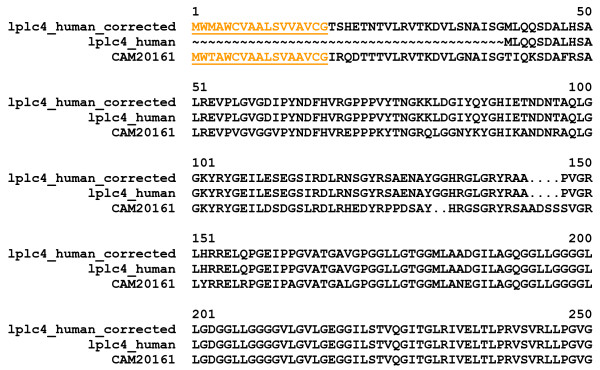
**Error detected by MisPred routine for Conflict 1: the case of the Swiss-Prot entry LPLC4_HUMAN**. The protein contains extracellular domains LBP_BPI_CETP and LBP_BPI_CETP_C but was found to lack both a signal peptide and transmembrane helices. The human sequence was corrected (LPLC4_HUMAN_corrected) by targeted search of the human genome with its mouse ortholog, CAM20161 [EMBL:CAM20161] that has a signal peptide. The alignment shows the N-terminal parts of LPLC4_HUMAN, CAM20161 and LPLC4_HUMAN_corrected. The predicted signal peptides of CAM20161 and LPLC4_HUMAN_corrected are in yellow and underlined.

**Figure 2:**
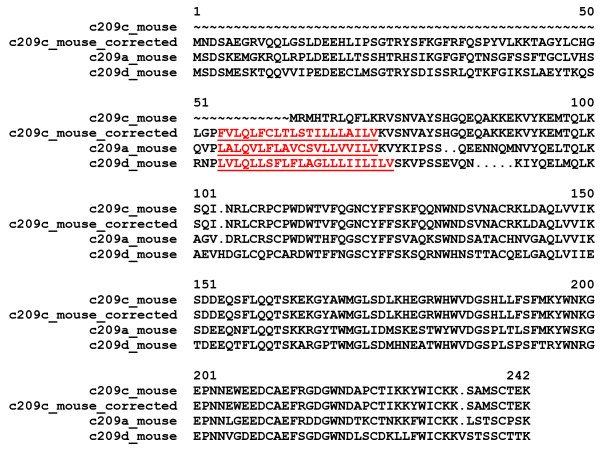
**Error detected by MisPred routine for Conflict 1: the case of the Swiss-Prot entry C209C_MOUSE**. The protein contains an extracellular C-type lectin domain but was found to lack both a signal peptide and transmembrane helices, whereas all closely related proteins (e.g. C209A_MOUSE, C209D_MOUSE [Swiss-Prot:Q91ZX1, Q91ZW8]) are type II transmembrane proteins. The sequence of this protein was corrected by targeted search of mouse genomic and EST sequences. The alignment shows the N-terminal parts of C209C_MOUSE, C209C_MOUSE_corrected, C209A_MOUSE and C209D_MOUSE. The predicted transmembrane helices of C209C_MOUSE_corrected, C209A_MOUSE and C209D_MOUSE are in red and underlined.

**Figure 3:**
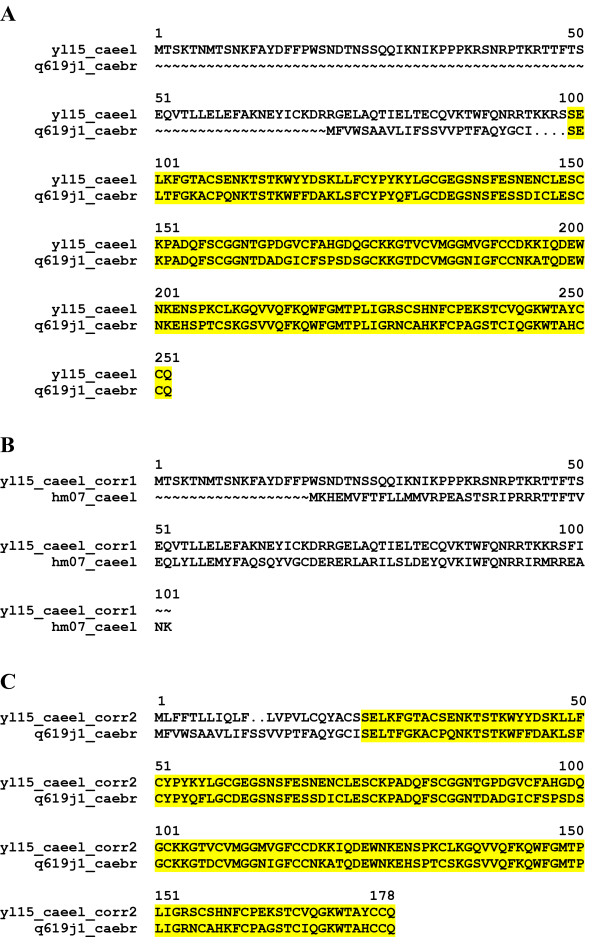
**Error detected by MisPred routine for Conflict 1: the case of the Swiss-Prot entry YL15_CAEEL **The hypothetical homeobox protein C02F12.5 [EnsEMBL: C02F12.5] predicted for chromosome X contains an extracellular Kunitz_BPTI domain but was found to lack both a signal peptide and transmembrane helices. This protein, that also contains a nuclear Homeobox domain, arose through *in silico *fusion of a gene related to the homeobox protein HM07_CAEEL and a gene related to the Kunitz_BPTI containing protein CBG14258, Q619J1_CAEBR. (A) Alignment of YL15_CAEEL and Q619JI_CAEBR shows close homology only in the C-terminal region, highlighted in yellow. (B) Alignment of the YL15_CAEEL_corr1 and HM07_CAEEL. (C) Alignment of YL15_CAEEL_corr2 and Q619J1_CAEBR.

There were three major types of true positives among the Swiss-Prot entries identified by Conflict 1:

1) Fragments of full-length proteins that are not known to be fragments and/or are not annotated as such in the database. For example, LPLC4_HUMAN [Swiss-Prot:P59827] proved to be a fragment and its missing signal peptide could be predicted with the help of the sequence of a full-length mouse ortholog (Figure 1). Similarly, the sequence of C209C_MOUSE [Swiss-Prot:Q91ZW9] (lacking a transmembrane helix) could be corrected by targeted search of mouse genomic and EST sequences (Figure 2).

2) Mispredicted proteins. The hypothetical worm protein YL15_CAEEL [Swiss-Prot:Q11101] is an example for this type of error. The protein arose through *in silico *fusion of a gene related to the homeobox protein HM07_CAEEL [Swiss-Prot:P20270] and a gene related to the Kunitz_BPTI containing protein CBG14258, Q619J1_CAEBR [TrEMBL:Q619J1] (Figure 3).

3) Proteins translated from aberrant transcripts that do not encode viable proteins. For example NOE2_MOUSE [Swiss-Prot:Q8BM13] lacks a signal peptide, whereas the rat ortholog [RefSeq:NP_001015017] and a different isoform of this mouse protein [GenBank:EDL25126] do possess a signal sequence.

The MisPred routine used for the detection of Conflict 1 is characterized by a very low number of false positives. There are three main sources of false positives:

1) Some proteins are identified as suspicious due to the limitations of the bioinformatic tools incorporated in this MisPred routine (e.g. failure to detect some signal peptides and transmembrane helices).

2) Exceptions to the dogma on which this MisPred routine is based, i.e. some secreted proteins truly lack secretory signal peptides since they are subject to leaderless protein secretion [22], such as the secreted proteins GAPR1_HUMAN [Swiss-Prot:Q9H4G4] and TINAG_HUMAN [Swiss-Prot:Q9UJW2].

3) Exceptions to the rule that all members of an extracellular domain family are restricted to the extracellular space.

**Figure 4:**
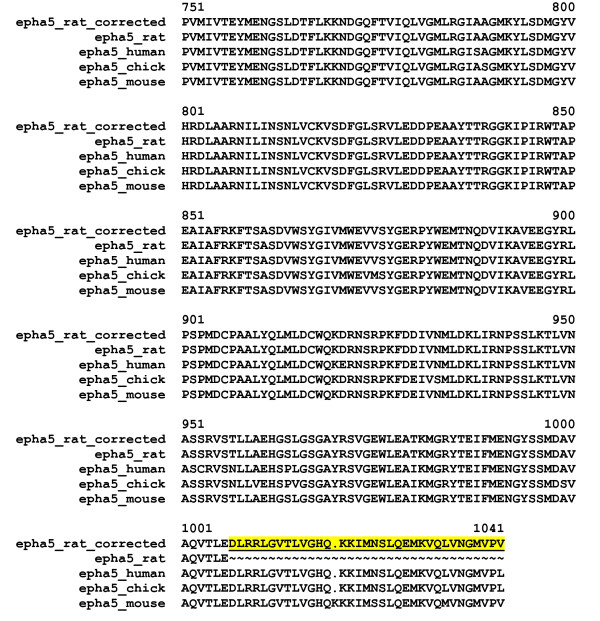
**Error detected by MisPred routine for Conflict 4: the case of the Swiss-Prot entry EPHA5_RAT**. This protein contains a C-terminal truncated SAM_1 domain that deviates significantly from the normal size of this domain family. It is noteworthy that orthologs from mouse, human and chicken contain an intact SAM_1 domain. The sequence of this protein was corrected by targeted search of the rat genome using the sequences of the full-length orthologs. The alignment shows the C-terminal parts of EPHA5_RAT, EPHA5_RAT_corrected, EPHA5_MOUSE [Swiss-Prot:Q60629], EPHA5_HUMAN [Swiss-Prot:P54756] and EPHA5_CHICK [Swiss-Prot:P54755]. The region of the predicted SAM_1 domain of EPHA5_RAT_corrected that is absent in EPHA5_RAT is underlined and highlighted in yellow.

MisPred routine based on Conflict 4 also identified a number of truly erroneous entries. A major source for this type of error is that the Swiss-Prot entry corresponds to an incomplete protein (with a truncated domain). For example, the sequence of EPHA5_RAT [Swiss-Prot: P54757] contains only a fragment of a SAM_1 domain since the protein sequence is truncated at the C-terminal end (Figure 4). The error in EPHA5_RAT could be corrected by targeted search of the rat genome using the sequences of the full-length orthologs (see Figure 4).

The routine based on Conflict 5 identified no erroneous mouse, chicken, worm or fruitfly Swiss-Prot proteins and the proportion of trans-chromosomal chimeras is very low in the case of human (0.01%) and rat (0.03%) sequences. On the other hand, the frequency of suspected chimeras is relatively high in the case of zebrafish (1.4%). As discussed in Additional file 1 [see Additional file 1], the most likely explanation for this observation is that they are false positives: the chromosomal assignment of contigs encoding different parts of these zebrafish genes may not be correct.

MisPred analyses have revealed that none of the Swiss-Prot entries violate the rules underlying Conflicts 2 and 3.

The fact that the number of Swiss-Prot entries identified by MisPred as erroneous is very low attests to both the high quality of this database and the reliability of the MisPred approach. Assuming that the vast majority of the Swiss-Prot sequences that were not detected by MisPred routines are true negatives the false positive rate of the routines was calculated to be ≤ 0.001, i.e. their specificity is very high (≥ 0.999).

### MisPred analysis of the TrEMBL section of UniProtKB

The primary motivation for MisPred analysis of UniProtKB/TrEMBL was that TrEMBL entries are used in various types of evidence-based, extrinsic gene prediction programs and thus have a strong influence on the quality of gene predictions. The results of the analyses of human proteins are summarized in Table 2.

**Table 2:** MisPred analysis of TrEMBL entries

**UniProtKB/TrEMBL**											
**Conflict 1**	Number of proteins	Identified as containing an extracellular domain	Percentage	Identified as suspicious by MisPred	Percentage*	False positives	Percentage*	True errors	Percentage*	Annotated as fragment or chimera by UniProt	Identified as abnormal only by MisPred

Homo sapiens	52237	6732	12.9%	3907	58.0%	ND	ND	ND	ND	ND	ND

**Conflict 2**	Number of proteins	Identified as containing an extra- and an intracellular domain	Percentage	Identified as suspicious by MisPred	Percentage*	False positives	Percentage*	True errors	Percentage*	Annotated as fragment or chimera by UniProt	Identified as abnormal only by MisPred

Homo sapiens	52237	58	0.11%	9	15.5%	9	15.5%	0	0.0%	0	0

**Conflict 3**	Number of proteins			Identified as suspicious by MisPred	Percentage*	False positives	Percentage*	True errors	Percentage*	Annotated as fragment or chimera by UniProt	Identified as abnormal only by MisPred
			
Homo sapiens	52237			0	0.0%	0	0.0%	0	0.0%	0	0

**Conflict 4**	Number of proteins	Proteins containing domains suitable for the study of domain integrity	Percentage	Identified as suspicious by MisPred	Percentage*	False positives	Percentage*	True errors	Percentage*	Annotated as fragment or chimera by UniProt	Identified as abnormal only by MisPred

Homo sapiens	52237	17073	32.7%	2531	14.8%	ND	ND	ND	ND	ND	ND

**Conflict 5**	Number of proteins			Identified as suspicious by MisPred	Percentage*	False positives	Percentage*	True errors	Percentage*	Annotated as fragment or chimera by UniProt	Identified as abnormal only by MisPred
			
Homo sapiens	52237			172	0.33%	0	0.00%	172	0.33%	85	87
Mus musculus	50304			40	0.08%	ND	ND	ND	ND	ND	ND
Rattus norvegicus	8557			5	0.06%	ND	ND	ND	ND	ND	ND
Gallus gallus	5549			6	0.11%	ND	ND	ND	ND	ND	ND
Danio rerio	19623			387	1.97%	ND	ND	ND	ND	ND	ND
Caenorhabditis elegans	30000			0	0.00%	0	0	0	0	0	0
Drosophila melanogaster	26947			49	0.18%	ND	ND	ND	ND	ND	ND

*Values for suspicious, false positive and true positive sequences are expressed as percentage of the proteins relevant for the given conflict.

ND – not determined

The data shown in Table 2 indicate that the proportion of suspicious TrEMBL entries is relatively high in the case of Conflict 1, Conflict 4 and Conflict 5. Importantly, these values are orders of magnitude higher than those for the Swiss-Prot entries, indicating that the vast majority of TrEMBL proteins identified by MisPred as suspicious are truly erroneous.

The majority (58.0%) of human TrEMBL proteins containing at least one extracellular domain were found by MisPred to lack a signal peptide and/or a transmembrane segment in contrast to 1.05% in the case of human Swiss-Prot entries. Similarly, 14.8% of human TrEMBL entries containing at least one member of the Pfam-A domain families suitable for the study of domain integrity were found to contain a domain of abnormal size, while this value is only 0.14% in Swiss-Prot. The reason why a high proportion of TrEMBL proteins are identified by Conflict 1 as suspicious is that many TrEMBL entries are truncated at the N-terminal end and N-terminally truncated secreted proteins are likely to lack the signal peptides. Similarly, the high proportion of TrEMBL entries affected by Conflict 4 reflects the severe contamination of this database with proteins predicted for incomplete cDNAs. Since cDNAs are more likely to be incomplete at their 5' end than their 3' end, the size of Pfam-A domains at the N-terminal end of proteins of the TrEMBL database was found to deviate more significantly from the average size than those of internal domains (data not shown), again indicating that a relatively large proportion of TrEMBL entries are truncated at the N-terminal end. In harmony with this explanation, 95% (Conflict 1) and 100% (Conflict 4) of the suspicious entries are also annotated as fragments in TrEMBL.

**Figure 5:**
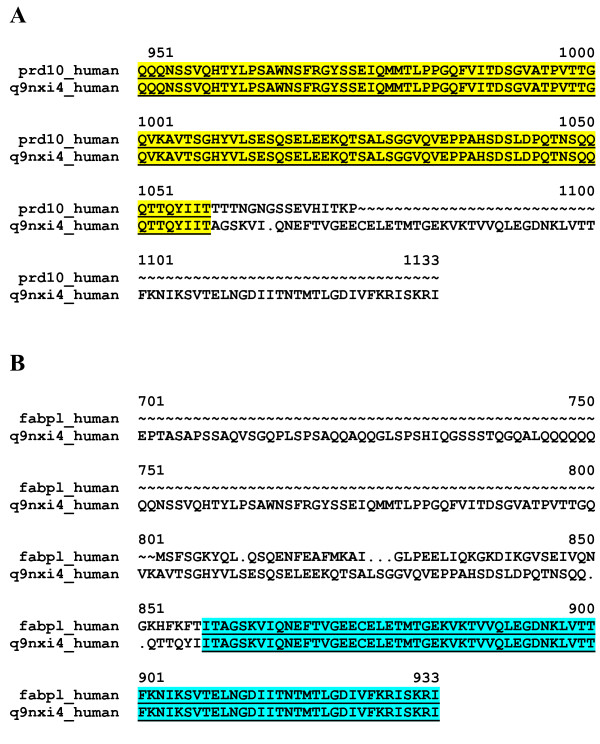
**Error detected by MisPred routine for Conflict 5: the case of the protein Q9NXI4_HUMAN**. The cDNA of this hypothetical protein FLJ20227, cloned from colon mucosa is derived from a chimera of two genes located on chromosome 11 and chromosome 2. The N-terminal part of the protein (underlined and highlighted in yellow) is derived from the gene encoding the PR domain zinc finger protein 10, PRD10_HUMAN (A), the C-terminal part of the protein (underlined and highlighted in blue) is derived from the gene encoding liver fatty acid-binding protein, FABPL_HUMAN (B).

Errors of TrEMBL entries are not only due to the incompleteness of cDNAs; transcripts formed through aberrant splicing and chimeric transcripts may also contribute to errors in this database. Interestingly, there are numerous human TrEMBL entries that are chimeric (0.33%), different segments of the predicted protein sequences being encoded by different genes located on different chromosomes. A large proportion (43.6%) of these chimeric entries are annotated as resulting from the fusion of genes located on different chromosomes through chromosomal translocation in a cancer cell line, 7.6% have no such annotation although the corresponding cDNAs were cloned from cancer tissues. It should be pointed out, however, that there are many chimeric proteins in UniProtKB derived from cDNAs that were cloned from apparently normal tissues (36.6%), suggesting that chimera formation is more general than previously thought. For example, the cDNA of the hypothetical protein FLJ20227 [TrEMBL:Q9NXI4], cloned from colon mucosa, is a chimera of two genes located on chromosome 11 and chromosome 2. The N-terminal part of the protein is derived from the gene encoding the PR domain zinc finger protein 10 (PRD10_HUMAN) [Swiss-Prot:Q9NQV6], the C-terminal part is derived from the gene encoding the liver form of Fatty acid-binding protein (FABPL_HUMAN) [Swiss-Prot:P07148] (see Figure 5). Another factor that increased the number of human chimeric proteins in TrEMBL is that the biotechnology industry has contributed numerous synthetic (chimeric) human constructs to the TrEMBL database (5.8%).

It is also noteworthy that the rate of chimeras is much higher in the case of zebrafish sequences (1.97%) than in the case of other vertebrates. As discussed in Additional file 1 [see Additional file 1], the most likely explanation for this observation is that the chromosomal assignment of contigs encoding these zebrafish genes may not be correct: contigs carrying different fragments of a zebrafish gene may be incorrectly assigned to different chromosomes.

MisPred routines for Conflict 2 and Conflict 3 identified no TrEMBL entries as erroneous. This is primarily due to the fact that sequences predicted *in silico *(that could miss internal transmembrane segments separating extracellular and cytoplasmic domains or fuse tandem genes) are absent from this section of UniProtKB.

### MisPred analysis of the EnsEMBL database and the GNOMON-predicted proteins of the NCBI database

Table 3 and Table 4 summarize the results of the analysis of the EnsEMBL database and the GNOMON-predicted proteins of the NCBI database for the various species and the five different MisPred routines (for details [see Additional file 1]).

**Table 3:** MisPred analysis of EnsEMBL entries

**EnsEMBL**									
**Conflict 1**	Number of proteins	Identified as containing an extracellular domain	Percentage	Identified as suspicious by MisPred	Percentage*	False Positives	Percentage*	True errors	Percentage*

Homo sapiens	48403	3449	7.13%	277	8.03%	ND	ND	ND	ND
Mus musculus	31302	2038	6.51%	151	7.41%	ND	ND	ND	ND
Rattus norvegicus	33745	2390	7.08%	325	13.6%	ND	ND	ND	ND
Monodelphis domestica	32690	2369	7.25%	661	27.9%	ND	ND	ND	ND
Gallus gallus	24168	1519	6.29%	413	27.19%	ND	ND	ND	ND
Xenopus tropicalis	28324	2383	8.41%	931	39.07%	ND	ND	ND	ND
Fugu rubripes	22102	1612	7.29%	627	38.9%	ND	ND	ND	ND
Danio rerio	36065	3312	9.18%	1224	36.96%	ND	ND	ND	ND
Ciona intestinalis	20000	1452	7.26%	670	46.14%	ND	ND	ND	ND
Caenorhabditis elegans	26439	918	3.47%	117	12.75%	ND	ND	ND	ND
Drosophila melanogaster	19789	1071	5.41%	120	11.2%	ND	ND	ND	ND

**Conflict 2**	Number of proteins	Identified as containing an extra- and an intracellular domain	Percentage	Identified as suspicious by MisPred	Percentage*	False Positives	Percentage*	True errors	Percentage*

Homo sapiens	48403	101	0.21%	18	17.82%	18	17.82%	0	0.00%
Mus musculus	31302	50	0.16%	4	8.00%	4	8.00%	0	0.00%
Rattus norvegicus	33745	67	0.2%	12	17.91%	10	14.93%	2	2.99%
Monodelphis domestica	32690	101	0.31%	25	24.75%	9	8.91%	16	15.84%
Gallus gallus	24168	45	0.19%	5	11.11%	4	8.89%	1	2.22%
Xenopus tropicalis	28324	57	0.2%	11	19.3%	5	8.77%	6	10.53%
Fugu rubripes	22102	58	0.26%	19	32.76%	12	20.69%	7	12.07%
Danio rerio	36065	75	0.21%	8	10.67%	7	9.33%	1	1.33%
Ciona intestinalis	20000	29	0.15%	2	6.90%	2	6.90%	0	0.00%
Caenorhabditis elegans	26439	12	0.05%	1	8.33%	1	8.33%	0	0.00%
Drosophila melanogaster	19789	16	0.08%	1	6.25%	1	6.25%	0	0.00%

**Conflict 3**	Number of proteins			Identified as suspicious by MisPred	Percentage*	False Positives	Percentage*	True errors	Percentage*
			
Homo sapiens	48403			1	0.002%	0	0.00%	1	0.002%
Mus musculus	31302			3	0.01%	0	0.00%	3	0.01%
Rattus norvegicus	33745			3	0.01%	0	0.00%	3	0.01%
Monodelphis domestica	32690			0	0.00%	0	0.00%	0	0.00%
Gallus gallus	24168			1	0.004%	0	0.00%	1	0.004%
Xenopus tropicalis	28324			0	0.00%	0	0.00%	0	0.00%
Fugu rubripes	22102			2	0.01%	0	0.00%	2	0.01%
Danio rerio	36065			0	0.00%	0	0.00%	0	0.00%
Ciona intestinalis	20000			0	0.00%	0	0.00%	0	0.00%
Caenorhabditis elegans	26439			0	0.00%	0	0.00%	0	0.00%
Drosophila melanogaster	19789			0	0.00%	0	0.00%	0	0.00%

**Conflict 4**	Number of proteins	Proteins containing domains suitable for the study of domain integrity	Percentage	Identified as suspicious by MisPred	Percentage*	False Positives	Percentage*	True errors	Percentage*

Homo sapiens	48403	16681	34.46%	850	5.1%	ND	ND	ND	ND
Mus musculus	31302	9955	31.80%	306	3.07%	ND	ND	ND	ND
Rattus norvegicus	33745	11826	35.05%	474	4.01%	ND	ND	ND	ND
Monodelphis domestica	32690	11847	36.24%	381	3.22%	ND	ND	ND	ND
Gallus gallus	24168	6261	25.91%	383	6.12%	ND	ND	ND	ND
Xenopus tropicalis	28324	6733	23.78%	318	4.72%	ND	ND	ND	ND
Fugu rubripes	22102	5464	24.72%	278	5.09%	ND	ND	ND	ND
Danio rerio	36065	9402	26.07%	591	6.29%	ND	ND	ND	ND
Ciona intestinalis	20000	2114	10.57%	147	6.95%	ND	ND	ND	ND
Caenorhabditis elegans	26439	3039	11.49%	86	2.83%	ND	ND	ND	ND
Drosophila melanogaster	19789	3341	16.88%	58	1.74%	ND	ND	ND	ND

**Conflict 5**	Number of proteins			Identified as suspicious by MisPred	Percentage*	False Positives	Percentage*	True errors	Percentage*
			
Homo sapiens	48403			0	0.00%	0	0.00%	0	0.00%
Danio rerio	36065			9	0.02%	7	0.02%	2	0.01%

*Values for suspicious, false positive and true positive sequences are expressed as percentage of the proteins relevant for the given conflict.

ND – not determined

**Table 4:** MisPred analysis of NCBI's GNOMON-predicted proteins

**NCBI/GNOMON**									
**Conflict 1**	Number of proteins	Identified as containing an extracellular domain	Percentage	Identified as suspicious by MisPred	Percentage*	False Positives	Percentage*	True errors	Percentage*

Homo sapiens	10125	287	2.83%	93	32.4%	ND	ND	ND	ND
Monodelphis domestica	20110	1293	6.43%	253	19.57%	ND	ND	ND	ND
Gallus gallus	14816	909	6.14%	246	27.06%	ND	ND	ND	ND
Danio rerio	25356	2108	8.31%	562	26.66%	ND	ND	ND	ND

**Conflict 2**	Number of proteins	Identified as containing an extra- and an intracellular domain	Percentage	Identified as suspicious by MisPred	Percentage*	False Positives	Percentage*	True errors	Percentage*

Homo sapiens	10125	4	0.04%	0	0%	0	0.00%	0	0.00%
Monodelphis domestica	20110	32	0.16%	6	18.75%	3	9.38%	3	9.38%
Gallus gallus	14816	22	0.15%	5	22.73%	3	13.64%	2	9.09%
Danio rerio	25356	31	0.12%	11	35.48%	5	16.13%	6	19.35%

**Conflict 3**	Number of proteins			Identified as suspicious by MisPred	Percentage*	False Positives	Percentage*	True errors	Percentage*
			
Homo sapiens	10125			0	0.00%	0	0.00%	0	0.00%
Monodelphis domestica	20110			2	0.01%	0	0.00%	2	0.01%
Gallus gallus	14816			2	0.01%	1	0.01%	1	0.01%
Danio rerio	25356			7	0.03%	3	0.01%	4	0.02%

**Conflict 4**	Number of proteins	Proteins containing domains suitable for the study of domain integrity	Percentage	Identified as suspicious by MisPred	Percentage*	False Positives	Percentage*	True errors	Percentage*

Homo sapiens	10125	1632	16.12%	255	15.63%	ND	ND	ND	ND
Monodelphis domestica	20110	6224	30.95%	111	1.78%	ND	ND	ND	ND
Gallus gallus	14816	3564	24.06%	370	10.38%	ND	ND	ND	ND
Danio rerio	25356	4387	17.31%	385	8.78%	ND	ND	ND	ND

**Conflict 5**	Number of proteins			Identified as suspicious by MisPred	Percentage*	False Positives	Percentage*	True errors	Percentage*
			
Homo sapiens	10125			1	0.01%	0	0.00%	1	0.01%
Danio rerio	25356			25	0.10%	24	0.09%	1	0.004%

*Values for suspicious, false positive and true positive sequences are expressed as percentage of the proteins relevant for the given conflict.

ND – not determined

As illustrated in Tables 3 and 4, a relatively high proportion of EnsEMBL and GNOMON-predicted entries are detected by the routine for Conflict 1 as suspicious (ranging from 8 to 46% for EnsEMBL and 20 to 32% for NCBI entries containing extracellular domains). The most likely explanation for this is that in a large proportion of secreted vertebrate proteins the signal peptide is encoded by an exon separated by a long intron from the downstream exon [23]. Low sequence conservation of signal peptides and low transcript- and EST-coverage of 5' parts of protein-coding genes explain why finding short exons encoding just the signal peptides has a rather low rate of success in the case of vertebrate genomes. This problem is less serious in the case of intron-poor genomes, such as those of *Caenorhabditis elegans *or *Drosophila melanogaster *since their signal peptides are less likely to be encoded by short, distinct exons [24]; this is reflected in a lower proportion of suspicious proteins in the case of EnsEMBL entries of these species. Furthermore, worm and fruitfly were the first metazoan organisms whose genome sequences were determined [25,26] and genome annotation efforts of nearly a decade have significantly improved the quality of gene predictions.

**Figure 6:**
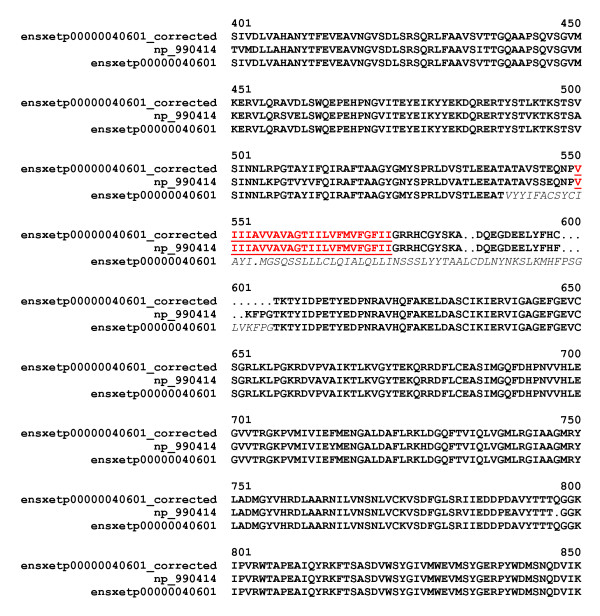
**Error detected by MisPred routine for Conflict 2**. ENSXETP00000040601 of *Xenopus tropicalis *corresponds to the frog ortholog of Ephrin receptor A7, but lacks a typical transmembrane helix between its extracellular FN3 and cytoplasmic Pkinase domains. The mispredicted sequence was corrected by identifying the missing transmembrane sequence using frog EST sequences such as EL820950 [GenBank:EL820950]. The alignment shows the regions containing the transmembrane helices of *Gallus gallus *Ephrin receptor A7 [RefSeq:NP_990414], ENSXETP00000040601 and ENSXETP00000040601_corrected. The predicted transmembrane helices of NP_990414 and ENSXETP00000040601_corrected are in red and underlined, the mispredicted region of ENSXETP00000040601 is in italics.

Very few erroneous EnsEMBL and GNOMON-predicted sequences were detected by the routine for Conflict 2. The explanation for the low rate of this type of error is that – unlike signal peptide segments – transmembrane helices are usually encoded by longer exons that also encode other conserved parts of transmembrane proteins, facilitating their detection. Another factor that facilitates detection of exons of transmembrane regions is that they are more likely to be located in the middle or 3' parts of genes whose transcript- and EST-coverage is relatively high. The true positives for Conflict 2 were found to be of two major types:

(1) The predicted protein lacks transmembrane helices since the corresponding region of the gene is mispredicted. For example, ENSXETP00000040601 [EnsEMBL: ENSXETP00000040601], which corresponds to the frog ortholog of Ephrin receptor A7, lacks a typical transmembrane helix between its extracellular and cytoplasmic domains; the missing transmembrane sequence could be corrected using frog EST sequences (Figure 6). Detailed analysis of this group of true positives (vertebrate EPH receptor tyrosine kinases, Tie-2 receptor tyrosine kinases, skeletal muscle receptor tyrosine kinases, receptor-type tyrosine-protein phosphatases, Notch proteins, etc.) revealed that the regions containing their transmembrane helices are encoded by relatively short exons distinct from those encoding conserved extracellular and cytoplasmic domains [27], making it difficult to find these exons.

(2) The gene was mispredicted by *in silico *fusion of distinct, tandem genes encoding extracellular and cytoplasmic proteins. Several examples of this type of error were found among *Fugu rubripes *proteins, but not in the case of other organisms, including zebrafish. A possible explanation for this observation is that the intergenic distance is significantly shorter in the compact genome of pufferfish than in the case of other vertebrate genomes [28], increasing the chance of *in silico *fusion of tandem genes.

MisPred routine for Conflict 3 detected very few errors in predicted proteins. Analyses of these sequences have revealed that they arose as a result of *in silico *fusion of two or more distinct, tandem genes encoding extracellular and nuclear proteins. Interestingly, proteins containing extracellular Pentaxin and nuclear Chromo domains were found among human, mouse, rat and chicken EnsEMBL proteins. There are several interpretations for their occurrence in different warm-blooded animals. One possible explanation is that since the constituent genes are closely linked in all these species, gene-prediction erroneously fused these otherwise independent genes. An alternative explanation is that these genes truly give rise to novel transcripts and proteins in which nuclear Chromo domains are fused to extracellular Pentaxin domains. In other words, nuclear Chromo domains can co-occur with the extracellular Pentaxin domains, either because the Chromo domain is not an obligatory nuclear domain or the Pentaxin domain is not an obligatory extracellular domain. It is noteworthy in this respect that Chen and Bixby [29,30] have cloned three mouse variants of neuronal pentraxin with Chromo domain (Q6TLW1_MOUSE, Q6TLW0_MOUSE, Q6TKP2_MOUSE) [Swiss-Prot:Q6TLW1, Q6TLW0, Q6TKP2] but none of them have signal peptides suggesting that, unlike the major products of the neuronal pentraxin genes, they are not secreted. It is still possible, however, that the fusion proteins encoded by chimeric transcripts are abnormal in the sense that they are non-viable. It is important to point out that such chimeric transcripts of neuronal pentraxin are very rare, or absent, as revealed by blast searches of EST databases. The fact that the rate of such errors is highest in the case of *Fugu rubripes *may be partly due to the short intergenic distance in the compact pufferfish genome.

MisPred routine for Conflict 4 detected a large number of erroneous proteins both among EnsEMBL entries (1.74–6.95%) and among GNOMON-predicted entries (1.78–15.63%) containing members of Pfam-A domain families suitable for the study of domain integrity. The relatively high rate of erroneous insertion or omission of exons encoding parts of domains indicates that misprediction of exons encoding Pfam-A domains is quite general.

No erroneous human EnsEMBL protein was detected by the routine for Conflict 5, i.e. none of them were chimeras of genes located on different chromosomes. This is not surprising in view of the fact that chromosomal assembly of human genomic contigs is reliable therefore EnsEMBL is exempt from the error of trans-chromosomal prediction of human genes. MisPred analysis of the *Homo sapiens *GNOMON-predicted entries has identified only one sequence, XP_001128605 [RefSeq:XP_001128605], as a chimera of genomic regions located on chromosomes 2 and 7. Note that in the NCBI database XP_001128605 has been replaced recently by the nonchimeric NP_001035225 [RefSeq: NP_001035225], encoded on chromosome 7. In contrast with this, several zebrafish entries were identified as suspicious by the MisPred routine for Conflict 5, an observation best explained by errors in the chromosomal assignment and assembly of zebrafish contigs. For example, ENSDARP00000056920 [EnsEMBL: ENSDARP00000056920] aligns over its entire length with mammalian Casein kinase I isoform gamma-2 proteins, but it is encoded by contigs assigned to chromosomes 2 and 8. XP_001345102 [RefSeq: XP_001345102] corresponds to a fragment of the zebrafish ortholog of ephrin receptor EPHA3, it aligns over its entire length with these receptors, but it is encoded by contigs assigned to chromosome 12 and 25. Incorrect assembly of contigs may lead to the *in silico *fusion of regions located on different chromosomes. For example, the major part of ENSDARP00000077525 [EnsEMBL:ENSDARP00000077525], encoded on chromosome 7, is homologous with mammalian Solute carrier organic anion transporter family member 3A1 proteins, but an unrelated N-terminal extension of ENSDARP00000077525 is encoded on chromosome 11. XP_001345729 [RefSeq: XP_001345729], a zebrafish protein annotated as similar to TRAF interacting protein is a chimera of traf-interacting protein and plasminogen related growth factor receptor 3.

In summary, GNOMON-predicted sequences and EnsEMBL sequences are quite similar inasmuch as similarly high proportion of suspicious proteins can be detected by Conflict 1 and Conflict 4 and fewer errors are detected by Conflict 2, Conflict 3 and Conflict 5 (see Tables 3 and 4). Despite these similarities, there are some differences. For example, in the case of Conflict 2 several EnsEMBL sequences were erroneous because the regions corresponding to their transmembrane helices were mispredicted, whereas this type of error was not found among the GNOMON-predicted sequences. In principle, such differences may reflect differences in the performance of the two gene prediction pipelines or differences in the gene populations covered by the two databases. It should also be pointed out that EnsEMBL is a comprehensive source of known genes and genes predicted with GeneWise [31], as well as the corresponding transcripts and proteins whereas in the NCBI database transcripts/proteins predicted by GNOMON are distinguished from those of known transcripts/proteins by unique (XM_ or XP_) identifiers.

To permit a more direct comparison of the performance of the two gene prediction pipelines we have compared the results of MisPred analyses only for those protein-coding genes for which both EnsEMBL and GNOMON have at least one prediction (see Table 5). Comparison of the two datasets confirmed that the two gene prediction pipelines are similar inasmuch as they suffer primarily from errors detectable by MisPred routines for Conflict 1 and Conflict 4, whereas the rates of errors detectable by routines for Conflicts 2, 3 and 5 are very low. Nevertheless, there are minor differences between the EnsEMBL and NCBI gene prediction pipelines: EnsEMBL is more likely to fail in the identification of exons encoding transmembrane helices, whereas NCBI's GNOMON appears to be more prone to fuse tandem genes *in silico *(for details of these analyses [see Additional file 1]).

**Table 5:** MisPred analysis of human genes predicted by the EnsEMBL and NCBI's GNOMON pipelines

**EnsEMBL**									
**Conflict 1**	Number of proteins	Identified as containing an extracellular domain	Percentage	Identified as suspicious by MisPred	Percentage*	False Positives	Percentage*	True errors	Percentage*

Homo sapiens	2772	147	5.3%	23	15.65%	ND	ND	ND	ND
Monodelphis domestica	10519	680	6.46%	137	20.15%	ND	ND	ND	ND
Gallus gallus	6139	345	5.62%	113	32.75%	ND	ND	ND	ND
Danio rerio	10289	860	8.36%	317	36.86%	ND	ND	ND	ND

**Conflict 2**	Number of proteins	Identified as containing an extra- and an intracellular domain	Percentage	Identified as suspicious by MisPred	Percentage*	False Positives	Percentage*	True errors	Percentage*

Homo sapiens	2772	1	0.04%	0	0.00%	0	0.00%	0	0.00%
Monodelphis domestica	10519	10	0.1%	0	0.00%	0	0.00%	0	0.00%
Gallus gallus	6139	2	0.03%	0	0.00%	0	0.00%	0	0.00%
Danio rerio	10289	20	0.19%	5	25%	4	20%	1	5%

**Conflict 3**	Number of proteins			Identified as suspicious by MisPred	Percentage*	False Positives	Percentage*	True errors	Percentage*
			
Homo sapiens	2772			0	0.00%	0	0.00%	0	0.00%
Monodelphis domestica	10519			0	0.00%	0	0.00%	0	0.00%
Gallus gallus	6139			0	0.00%	0	0.00%	0	0.00%
Danio rerio	10289			0	0.00%	0	0.00%	0	0.00%

**Conflict 4**	Number of proteins	Proteins containing domains suitable for the study of domain integrity	Percentage	Identified as suspicious by MisPred	Percentage*	False Positives	Percentage*	True errors	Percentage*

Homo sapiens	2772	722	26.05%	48	6.65%	ND	ND	ND	ND
Monodelphis domestica	10519	3726	35.42%	119	3.19%	ND	ND	ND	ND
Gallus gallus	6139	1640	26.72%	159	9.70%	ND	ND	ND	ND
Danio rerio	10289	2565	24.93%	197	7.68%	ND	ND	ND	ND

**Conflict 5**	Number of proteins			Identified as suspicious by MisPred	Percentage*	False Positives	Percentage*	True errors	Percentage*
			
Homo sapiens	2772			0	0.00%	0	0.00%	0	0.00%
Danio rerio	10289			0	0.00%	0	0.00%	0	0.00%

**NCBI/GNOMON**									

**Conflict 1**	Number of proteins	Identified as containing an extracellular domain	Percentage	Identified as suspicious by MisPred	Percentage*	False Positives	Percentage*	True errors	Percentage*

Homo sapiens	3012	139	4.61%	32	23.02%	ND	ND	ND	ND
Monodelphis domestica	9703	642	6.62%	112	17.45%	ND	ND	ND	ND
Gallus gallus	5604	310	5.53%	88	28.39%	ND	ND	ND	ND
Danio rerio	8905	742	8.33%	158	21.29%	ND	ND	ND	ND

**Conflict 2**	Number of proteins	Identified as containing an extra- and an intracellular domain	Percentage	Identified as suspicious by MisPred	Percentage*	False Positives	Percentage*	True errors	Percentage*

Homo sapiens	3012	2	0.07%	0	0%	0	0.00%	0	0.00%
Monodelphis domestica	9703	17	0.18%	4	23.53%	2	11.76%	2	11.76%
Gallus gallus	5604	3	0.05%	1	33.33%	1	33.33%	0	0.00%
Danio rerio	8905	16	0.18%	6	37.5%	4	25%	2	12.5%

**Conflict 3**	Number of proteins			Identified as suspicious by MisPred	Percentage*	False Positives	Percentage*	True errors	Percentage*
			
Homo sapiens	3012			0	0.00%	0	0.00%	0	0.00%
Monodelphis domestica	9703			1	0.01%	0	0.00%	1	0.01%
Gallus gallus	5604			0	0.00%	0	0.00%	0	0.00%
Danio rerio	8905			2	0.02%	1	0.01%	1	0.01%

**Conflict 4**	Number of proteins	Proteins containing domains suitable for the study of domain integrity	Percentage	Identified as suspicious by MisPred	Percentage*	False Positives	Percentage*	True errors	Percentage*

Homo sapiens	3012	792	26.3%	41	5.18%	ND	ND	ND	ND
Monodelphis domestica	9703	3420	35.25%	39	1.14%	ND	ND	ND	ND
Gallus gallus	5604	1500	26.77%	208	13.87%	ND	ND	ND	ND
Danio rerio	8905	2059	23.12%	300	14.57%	ND	ND	ND	ND

**Conflict 5**	Number of proteins			Identified as suspicious by MisPred	Percentage*	False Positives	Percentage*	True errors	Percentage*
			
Homo sapiens	3012			1	0.03%	0	0.00%	1	0.03%
Danio rerio	8905			5	0.06%	5	0.06%	0	0.00%

*Values for suspicious, false positive and true positive sequences are expressed as percentage of the proteins relevant for the given conflict.

ND – not determined.

The data refer to human genes for which both gene prediction pipelines generated at least one gene model.

### MisPred analysis of GENCODE sequences

The ENCODE (ENCyclopedia Of DNA Elements) Project aims to identify all functional elements in the human genome sequence. The pilot phase of the project is focused on specified 30 megabases (approximately 1%) of the human genome sequence [32]. GENCODE is a sub-project of ENCODE; its overall goal is to identify all protein-coding genes in the regions of the human genome selected within the ENCODE project.

MisPred analysis of the 1097 GENCODE peptides with the routine for Conflict 1 identified one peptide [GENCODE:AC110015.1-002] as containing an extracellular (Cadherin) domain but lacking signal peptide and transmembrane segments. Blast searches revealed that AC110015.1-002 corresponds to the N-terminal part of CADH2_HUMAN [Swiss-Prot:P19022] but, due to alternative splicing, it lacks the N-terminal secretory signal peptide. It seems unlikely that this transcript encodes a viable protein since – in the absence of a secretory signal peptide – it may not be transported into the extracytoplasmic space.

MisPred analysis of the GENCODE peptides for Conflict 4 identified 67 (6.1% of the total) as containing an abnormally short or abnormally long Pfam-A domain. In the majority of cases the deviant domains are N-terminally or C-terminally truncated simply as a consequence of the incompleteness of the transcripts. Nevertheless, we identified several cases where the domain deviates from normal size as a result of alternative splicing [33].

Examples include AC015691.9-002 [GENCODE:AC015691.9-002] (corresponding to TRIM6_HUMAN [Swiss-Prot:Q9C030]), RP11-247A12.5-001 [GENCODE:RP11-247A12.5-001] (corresponding to CACP_HUMAN [Swiss-Prot:P43155]), XX-FW83563B9.3-006 [GENCODE:XX-FW83563B9.3-006] (corresponding to TAZ_HUMAN [Swiss-Prot:Q16635]), AP006216.3-003 [GENCODE:AP006216.3-003] (corresponding to ZPR1_HUMAN [Swiss-Prot:O75312]), RP11-298J23.1-003 [GENCODE:RP11-298J23.1-003] (corresponding to PEPC_HUMAN [Swiss-Prot:P20142]) and RP11-247A12.4-008 [GENCODE:RP11-247A12.4-008] (corresponding to PTPA_HUMAN [Swiss-Prot:Q15257]) containing domains that deviate from normal size as a result of alternative splicing. Structural and functional analyses of the putative proteins encoded by these alternatively spliced transcripts suggest that, in many cases, the deviation from normal domain-size may not be compatible with the viability of these proteins, suggesting that the transcripts arose through aberrant splicing [33].

MisPred analysis of the GENCODE peptides for Conflicts 2, 3 and 5 did not identify suspicious sequences.

## Conclusion

MisPred may identify proteins as suspicious for three different reasons.

### MisPred may identify normal, viable proteins as suspicious due to errors of genomic data and limitations of the bioinformatic tools incorporated in MisPred

MisPred analyses of predicted zebrafish sequences illustrate the point that a 'correct' protein sequence may be suspected to be a chimera encoded by two or more genes located on different chromosomes if the assembly of the contigs is incorrect.

Errors in the bioinformatic identification of sequence features used to detect the various types of Conflicts may raise unjustified doubts about the viability of some protein sequences. For example, this type of error is encountered in the case of some secreted or transmembrane proteins (identified as such by the presence of obligatory extracellular domains) whose atypical signal peptides or transmembrane helices are not detected with high confidence by the signal peptide or transmembrane helix prediction programs incorporated into the MisPred routines. Similarly, the hmmpfam program may on occasion identify marker domains erroneously, leading to the incorrect prediction of the subcellular localization of a protein domain.

Analyses of the benchmark Swiss-Prot proteins, however, have revealed that the false positive rates of the MisPred routines are lower than 0.001%. For details of the specificity analyses see Materials and methods.

### MisPred may identify normal, viable proteins as suspicious due to limitations of the dogmas on which MisPred routines are based

A survey of the results of MisPred analyses has revealed that there are some exceptions to the dogmas on which the MisPred approach is based. For example, some secreted proteins may truly lack secretory signal peptides since they are subject to leaderless protein secretion [22], some predominantly extracellular, cytoplasmic or nuclear Pfam-A domain families are not always restricted to a single subcellular location and thus may be multilocale etc. Similarly, it cannot be excluded at present that transchromosomal chimeras can be formed and may have normal physiological functions [16]. Nevertheless, the fact that MisPred analyses of protein sequences of the Swiss-Prot database identified very few such exceptions indicates that the dogmas of MisPred are generally valid.

### MisPred may identify truly abnormal, nonviable proteins

MisPred analyses of Swiss-Prot, TrEMBL, EnsEMBL, NCBI and GENCODE sequences identified numerous hypothetical protein sequences that are likely to be nonviable since they violate some of the basic dogmas about viable proteins.

For example, analysis of the TrEMBL sequences revealed that incomplete or abnormal hypothetical proteins translated *in silico *from incomplete cDNAs or aberrant transcripts are quite abundant in this database. A recent study [33] has also revealed that alternative splicing frequently generates transcripts that encode nonviable proteins due to violation of domain integrity, loss of signal peptides, etc.

Interestingly, there are also numerous chimeric entries in the TrEMBL database, a large proportion of which are derived from normal tissues. There are several mechanisms for the creation of chimeric proteins. Unequal crossing over resulting in the fusion of parts of tandem genes and chromosomal translocation resulting in the fusion of genes located on different chromosomes are the two best documented mechanisms for the creation of chimeric genes and proteins. Recent studies have convincingly shown that transcription of tandem genes into a single RNA sequence and translation of the chimeric mRNA into a chimeric protein [34,35] is also a major mechanism for the creation of chimeric proteins. Unneberg and Claverie [36] have recently suggested that chimeric proteins might also be formed through transchromosomal transcription, i.e. if genes located on different chromosomes are expressed in the same "transcription factory" they may give rise to chimeric transcripts.

It should be pointed out that the relatively high proportion of incomplete and abnormal entries in TrEMBL also has an impact on gene prediction. Since most gene prediction pipelines rely on extrinsic information [5] such as those provided by TrEMBL, some of the errors of TrEMBL may be inherited by the databases of predicted genes. It is noteworthy in this respect that the majority of errors of EnsEMBL and GNOMON-predicted sequences can be detected with the use of Conflict 1 and Conflict 4, probably reflecting the fact the TrEMBL sequences also suffer from the same types of errors. The relatively high proportion of transchromosomal chimeric sequences among TrEMBL entries, however, does not have a major impact on gene prediction, provided that contig assembly is unambiguous.

### Sequences not detected by MisPred do not necessarily correspond to normal, viable proteins

Although MisPred identifies many suspicious sequences, it should be emphasized that the routines detect only a fraction of the truly erroneous sequences. First, the MisPred routines described in this manuscript exploit only five of the various dogmas about viable proteins. Second, only a fraction of proteins contains members of well-characterized Pfam-A domain families, i.e. families on which the routines for Conflicts 1, 2, 3 and 4 rely. Third, the number of suspicious sequences identified by Conflicts 1, 2 or 3 significantly underestimates the actual number of sequences that may be affected by these types of errors since we used only validated, obligatory extracellular, cytoplasmic and nuclear Pfam-A domain families to predict the subcellular localization of proteins and did not include numerous extracellular, cytoplasmic or nuclear domain families the members of which may also occur in other subcellular compartments. Fourth, in the case of Conflict 4 MisPred uses a high cut-off value: a domain is judged to be nonviable only if its Pfam-A domain deviates from normal size by at least 40% in length. For details of the sensitivity analyses see Materials and methods.

## Summary

Recent studies have shown that a significant proportion of eukaryotic genes may be mispredicted at the transcript level [5,6]. Since the MisPred routines described here are able to detect many of these errors and may aid the correction of these errors we suggest that the MisPred approach may significantly improve the quality of protein sequence data based on gene-predictions. In order to increase the sensitivity of the approach we are currently developing additional routines, based on the violation of other dogmas about proteins. The MisPred approach may also be used as a discovery tool since it can also serve to explore the limitations of the dogmas on which the various MisPred routines are based.

## Methods

### Protein sequence data analyzed

#### Protein sequence databases

In the analysis of the Swiss-Prot section of the UniProtKB we have included Metazoan species that have at least 1000 Swiss-Prot entries. The UniProtKB Swiss-Prot [37] entries from UniProtKB Version 9.5 (January, 2007) were downloaded from .

The UniProtKB TrEMBL [37] entries from UniProtKB Version 10.5 (May, 2007) were downloaded from .

The 1097 GENCODE protein sequences were obtained from .

The protein sequences of the EnsEMBL database were downloaded from the EnsEMBL website [38], release 41 (October, 2006), found at .

The NCBI's protein sequences of four species (*Homo sapiens*, 15 September 2006, *Monodelphis domestica*, 06 March 2007, *Gallus gallus*, 30 November 2006, *Danio rerio*, March 2007), were obtained by downloading the relevant protein.fa.gz files from the NCBI Genome Data/Annotation Projects website [39], found at . In order to analyze only the sequences predicted by GNOMON , an in-house program was used to extract only GNOMON-predicted FASTA sequences with 'XP_' identifiers.

#### Comparison of the EnsEMBL and NCBI/GNOMON gene predictions

In order to compare the reliability of the two gene prediction pipelines, we analyzed the proportion of suspicious sequences among predicted proteins represented in both EnsEMBL and NCBI's GNOMON-predicted section from four evolutionarily distant species (*Homo sapiens*, *Monodelphis domestica, Gallus gallus *and *Danio rerio*).

To identify the number of genes in each species for which both GNOMON and EnsEMBL have at least one predicted protein sequence, blastp [40] searches were performed on the GNOMON-predicted sequences using EnsEMBL sequences as queries. Protein sequences that displayed 100% identity over at least 25 amino acid residue-long ungapped segments were considered to be encoded by the same gene. The blastp standalone program, version 2.2.13, was obtained from: .

### Prediction of the subcellular localization of proteins by the presence of obligatory extracellular, cytoplasmic or nuclear protein-domains

Recent studies have revealed that there is a strong correlation between the domain-composition of proteins and their subcellular location: some domains are restricted to proteins targeted to the extracellular space, others occur only in proteins present in the cytoplasmic space, whilst others are restricted to proteins of the nucleus [17,18]. Transmembrane multidomain proteins are special in the sense that obligatory extracellular and cytoplasmic domains can legitimately co-occur in a single protein. Accordingly, the presence of obligatory extracellular, cytoplasmic or nuclear domains in a protein may be used to predict its subcellular localization, independent of the detection of sorting signals.

Since domains are most likely to co-occur in multidomain proteins if they belong to the same localization-category, analysis of domain co-occurrence networks is useful for the systematic assignment of domains to different subcellular compartments [17,18]. Our domain co-occurrence analyses of Metazoan UniProtKB entries have identified 166 obligatory extracellular, 115 obligatory cytoplasmic and 126 obligatory nuclear Pfam-A domain families as being restricted to the respective subcellular compartment, the majority of which are also identified as such in the SMART database  [see Additional files 3, 4 and 5], respectively. In the MisPred analyses described in the present work only these obligatory extracellular or cytoplasmic or nuclear domain families were used to predict subcellular localization. Pfam-A domains that are known not to be restricted to a particular cellular compartment, such as immunoglobulin domains, fibronectin type III domains, von Willebrand factor type A domains (i.e. domains that are 'multilocale'), are not reliable predictors of subcellular localization and thus they were not utilized in these analyses.

The programs of the HMMER 2.3.2 software package were used to detect obligatory extracellular, cytoplasmic and nuclear Pfam-A domains. HMM databases of Pfam-A domains were created by retrieving the HMMs of the domains from the Pfam (Pfam_ls) HMM and the Pfam fragment (Pfam_fs) HMM libraries [41]. The presence of Pfam-A domains in protein sequences was detected by searching the HMM databases against protein sequences using the hmmpfam program using 0.00001 as per-domain E-value threshold. We filtered the results for overlapping domain matches and the match with the lowest E-value was accepted.

The Pfam HMM libraries (Release 20.0) were obtained from . The HMMER software package was obtained from .

### Detection of suspicious (incomplete, abnormal and mispredicted) proteins

#### Conflict 1: Conflict between the predicted subcellular localization of proteins and the absence of the corresponding sequence signals

Proteins containing obligatory extracellular Pfam-A domains were analyzed by the PrediSi program [42] to identify the presence of eukaryotic signal peptide sequences (using 0.3 as threshold) and by the TMHMM program [43] to detect the presence of transmembrane helices. Protein sequences containing obligatory extracellular domains but neither a signal peptide nor a transmembrane helix were identified as suspicious. The PrediSi program was downloaded from . The TMHMM program was obtained from .

#### Conflict 2: Presence of obligatory extracellular and cytoplasmic domains and the absence of transmembrane helices

Proteins containing both obligatory extracellular and obligatory cytoplasmic Pfam-A domains were analyzed by the TMHMM program to detect transmembrane helices. Sequences, which contain both extra- and cytoplasmic domains (i.e. putative transmembrane proteins), but do not have a transmembrane helix were identified as suspicious.

#### Conflict 3: Co-occurrence of extracellular and nuclear domains in a single protein

Protein sequences containing both obligatory extracellular and obligatory nuclear domains were identified as suspicious.

#### Conflict 4: Violation of domain integrity

In order to identify the Pfam-A domain families that have a well-defined, conserved sequence length range, we selected only those families whose members do not deviate from the average size by more than 2 standard deviation (SD) values in the high quality Swiss-Prot database (version 48.9). Based on these criteria, about 90% of the Pfam-A domain families present in Swiss-Prot proteins proved to be suitable for the study of domain integrity. The list of the Pfam-A families is deposited in Additional file 6 [see Additional file 6]. We created databases of human, vertebrate and metazoa+fungi Swiss-Prot domain sequences belonging to the reliable Pfam-A families and ran a blastp search with the current set of proteins as queries against the appropriate reliable Swiss-Prot domain sequences. We selected those partial domain matches which share over 60% identity with the query sequence, with an E-value < 1e^-5^, and differ by at least 40% in length. The protein sequences containing these domains with deviant lengths were identified as suspicious. For the details of this method [see Additional file 1].

#### Conflict 5: Chimeric proteins encoded by two or more different genes located on different chromosomes

The protein sequences were matched to the genome of the given species using the BLAT program [44]. We selected matches with >95% identity over ≥ 15 amino acid residue in length and in the case of overlapping matches (if the overlap was >5 residues) we selected the longest match. To eliminate problems encountered with genes encoded by the mitochondrial genome [see Additional file 1] we used an additional BLAT search and discarded those entries which gave >90% match with the mitochondrial genome over more than 90% of their length. Proteins were considered suspicious if two or more of their segments were encoded on different chromosomes. The BLAT program was obtained from . The following genome assemblies were used in these analyses: *Homo sapiens *NCBI 36, Mar 2006; *Mus musculus *NCBI 36, Febr 2006; *Rattus rattus *RGSC 3.4, Nov 2004; *Gallus gallus *WASHUC2, May 2006; *Danio rerio *Zv6, Mar 2006;; *Caenorhabditis elegans *WS170, Jan 2007; *Drosophila melanogaster *BDGP 5, Apr 2006.

### Testing the specificity and sensitivity of MisPred methods

#### Specificity

To calculate the false positive rate (α) and specificity (1-α) of MisPred from the equation α = FP/(FP+TN), we have determined the number of false positives (FP) and true negatives (TN) from the results obtained by application of MisPred to Swiss-Prot entries. In these calculations FP equals the number of entries that were identified with the given method as suspicious, although they do not to violate the given dogma. Considering that Swiss-Prot is a very clean database, the entries not identified by MisPred as suspicious were assumed to be true negatives (TN), i.e. they do not violate the given dogma. Specificity was also calculated by analyzing datasets obtained by mixing correct Swiss-Prot entries and erroneous sequences generated from Swiss-Prot sequences (see below). The false positive rates of the MisPred routines were calculated to be ≤ 0.001, i.e. their specificity is very high (≥ 0.999).

#### Sensitivity

Since at the protein level the major types of errors of gene prediction (failure to find a true exon, erroneous inclusion of a false exon, misprediction of an exon, fusion of exons of tandem genes etc.) are manifested as internal or terminal deletions, internal insertions, terminal extensions and fusions, we have generated datasets of sequences from human Swiss-Prot entries to mimic these errors.

To test the effect of terminal deletions, a group of datasets was created through deletion of 50, 100, 150, 200 etc. residues from their N-terminal end or their C-terminal end. Another group of datasets were obtained by deleting the second, third, fourth etc. 50 or 100 residue-segments of the proteins to study the effect of internal deletions. Terminal extensions or internal insertions were mimicked by addition/insertion of 50 or 100 amino acid segments (with random sequences and average amino acid composition) to the N-terminal and C-terminal end or after positions 50, 100, 150 etc. of these proteins. To mimic the effect of fusions datasets were generated by fusing 5000 randomly selected entries to a different set of 5000 randomly selected proteins.

Subsets of the above datasets were created to gain insight into the factors that influence the sensitivity of the different MisPred methods. The false negative rate (β) and sensitivity (1-β) of MisPred were calculated from the equation β = FN/(TP + FN). Since all entries in these datasets are erroneous (i.e. they differ from the correct sequence), entries detected by MisPred as suspicious are true positives (TP), whereas those not detected by MisPred are false negatives (FN).

The MisPred routine for Conflict 1 detected only 4.5% of the sequences from which the N-terminal 50 residues (that might contain secretory signal peptide or signal-anchor sequences) were removed. One major source of such a low sensitivity is that only a fraction of proteins are secreted or type II transmembrane proteins, the integrity of which can be tested with this method. When we analyzed a dataset generated by N-terminal truncation of proteins containing signal peptide or signal anchor sequences but lacking transmembrane segments, sensitivity increased to 33.1%. The reason why only about a third of the erroneous proteins are detected by MisPred is that the majority of these proteins does not contain an obligatory extracellular Pfam-A domain and is thus 'invisible' to this method. When we restricted MisPred analysis to erroneous entries generated from secreted and type II transmembrane proteins containing an obligatory extracellular Pfam-A domain, sensitivity increased to 87.2%.

Only a very small fraction (0.79%) of the proteins possessing transmembrane helices are detected as erroneous by MisPred routine for Conflict 2 after removing their transmembrane helices. This is due to the fact that few of the transmembrane proteins contain both an obligatory extracellular and an obligatory cytoplasmic Pfam-A domain. If, however, we applied this method to erroneous entries generated from transmembrane proteins containing both an obligatory extracellular and an obligatory cytoplasmic Pfam-A domain, sensitivity increased to 83.7%.

The sensitivity of the MisPred routine for Conflict 3 was found to be 1.06% when we applied it to chimeric proteins generated by random fusion of proteins. Such a low sensitivity is due to several factors. First, nuclear proteins account for only ~14% of the entire proteome [45]. Second, our analyses have shown that only 26.7% of the nuclear proteins identified by Fink et al. [45] (2008) contain obligatory nuclear domains [see Additional file 5]. Third, only a fraction of proteins contains an obligatory extracellular Pfam-A domain. In harmony with this explanation, the sensitivity of the MisPred routine for Conflict 3 was found to be 99.9% when we applied it to chimeric proteins generated by fusion of human Swiss-Prot proteins containing obligatory extracellular domains to proteins containing obligatory nuclear domains.

The sensitivity of the MisPred routine for Conflict 4 was found to depend on the extent of terminal truncation of the protein sequences. Progressive truncation from the C-terminal or N-terminal end increased the sensitivity to a maximum of ~4% when ~250 residues were deleted, deletion of longer segments did not further increase sensitivity. The explanation for this observation is that this type of error becomes undetectable if the entire domain or the major part of the domain is removed. Sensitivity was found to be ~0.6% (or ~3%) in the case of internal deletions of 50 residue (or 100 residue) segments, and ~0.1% (or 4%) in the case of internal insertions of 50 (or 100) residue-long segments with random amino acid sequences, irrespective of the positions of the moving window deletions or the insertions. Addition of such random sequences to the N-terminal end, C-terminal end of human Swiss-Prot entries did not generate errors detectable by MisPred routine for Conflict 4. The relatively low sensitivity of the MisPred routine for Conflict 4 is due to the fact that only a fraction of human Swiss-Prot proteins contain a Pfam-A domain suitable for the detection of domain size deviation (see Table 1). Another factor that contributes to the low sensitivity of this approach is that a protein is detected as erroneous only if its Pfam-A domain deviates from normal size by at least 40% in length (see above).

The sensitivity of the MisPred routine for Conflict 5 was found to be very high (91.5%), when tested on artificial chimeras generated by random fusion of human Swiss-Prot proteins. Analysis of the few false negatives revealed that some of the chimeric proteins were not detected because the constituent proteins are encoded on the same chromosome, in other cases the BLAT match was below the 95% threshold. If we restricted the analyses to chimeras generated by fusion of genes encoded on different chromosomes, sensitivity increased to 92.9%.

### Resolution of Conflicts

In order to test whether a suspicious protein sequence identified by one of the MisPred routines is truly erroneous (or a false positive) we subjected these sequences to additional analyses. Such analyses were performed for all suspicious Swiss-Prot entries identified by all five MisPred routines as well as for all EnsEMBL, GNOMON-predicted and human TrEMBL entries identified by MisPred routines for Conflicts 2, 3 and 5 (for details [see Additional file 1]).

Sequences identified by MisPred routines for Conflict 1 and Conflict 2 as suspicious were analyzed by the SignalP [46] and Phobius [47] programs to detect signal peptides and/or transmembrane helices potentially missed by the PrediSi and/or the TMHMM programs.

To identify cases of non-classical, i.e. not signal peptide triggered protein secretion we used the SecretomeP 1.0b Server [22]. The SecretomeP 1.0b server produces *ab initio *predictions of non-classical protein secretion in eukaryotes. It should be noted, however, that this approach is not necessarily able to decide whether a given entry is an example of leaderless secretion or it is a fragment of a classically secreted protein (that lacks its signal peptide). To exclude the latter possibility, we have also queried EST databases to decide whether the initiating methionine is properly defined or the open reading frame may be extended in the upstream direction.

To further test whether a suspicious protein sequence (sequence A) identified by one of the MisPred routines is truly erroneous it was used as query to search protein and nucleic acid sequence databases with blastp and tblastn, respectively, to identify homologous sequences from the same as well as from other species. If the search yielded a perfect match (100% identity over at least 25 amino acid residues) with a different protein sequence from the same species (sequence B), but the latter protein was judged 'normal' by the same MisPred routine that identified sequence A as suspicious then we concluded that the error has been both validated and corrected (by sequence B). If the sequence similarity search identified close homologs of sequence A (paralogs from the same species, orthologs and/or paralogs from related species) but the latter protein(s) were judged 'normal' by the same MisPred routine that identified sequence A as suspicious then we concluded that the error has been validated. In the majority of such cases the erroneous sequence A could be corrected through targeted search of the appropriate genomic region of the relevant species with various gene prediction programs as well as search of EST databases, using the 'normal' homologous sequence(s) as queries. The protocol used for the correction of errors will be described in another publication (manuscript in preparation).

## Authors' contributions

AN, KF, HT and EK have developed the MisPred methods for Conflicts 1, 2, 3 and 5, HH developed the MisPred method for Conflict 4. LB was involved in bioinformatic analyses of the protein sequences identified as suspicious by the different MisPred routines and in correction of erroneous sequences. LP was involved in conceiving and planning the project.

## Supplementary Material

Additional file 1 Comments on entries identified by MisPred routines as suspicious and detailed description of Conflict 4. The file describes detailed analyses and comments of the entries identified as suspicious by the different MisPred routines, and contains the detailed description of the procedure of the identification of suspicious sequences by Conflict 4. Click here for file

Additional file 2 List of erroneous Swiss-Prot sequences identified by MisPred. The file contains the list of erroneous Swiss-Prot sequences identified by MisPred. Click here for file

Additional file 3 List of extracellular Pfam-A domain families. The file contains the list of extracellular Pfam-A domain families. Click here for file

Additional file 4 List of intracellular Pfam-A signaling domain families. The file contains the list of intracellular Pfam-A signaling domain families. Click here for file

Additional file 5 List of nuclear Pfam-A domain families. The file contains the list of nuclear Pfam-A domain families. Click here for file

Additional file 6 List of Pfam-A domain families suitable for the study of domain integrity. The file contains the list of Pfam-A domain families suitable for the study of domain integrity. Click here for file
