# Supplementary material for: Identification and correction of abnormal, incomplete and mispredicted proteins in public databases
Source: BMC Bioinformatics. 2008 Aug 27;9:353. doi: 10.1186/1471-2105-9-353 (PMC2542381; doi:10.1186/1471-2105-9-353)

Additional file 1. Comments on entries identified by MisPred routines as suspicious and detailed description of Conflict 4. Part 1 contains comments on suspicious Swiss-Prot, EnsEMBL- and GNOMON-predicted proteins, and on EnsEMBL and NCBI entries predicted for the same set of genes. Part 2 contains detailed description of the procedure of the identification of suspicious proteins by Conflict 4.

Part 1. Comments on entries identified by MisPred routines as suspicious

Comments on Swiss-Prot entries identified by MisPred routines as suspicious

Conflict 1

Analysis of the *Mus musculus* Swiss-Prot proteins by MisPred identified 12 proteins as suspicious. Four of the suspicious proteins proved to be false positives: although PrediSi failed to detect their signal peptides these were detected with the SignalP and/or Phobius programs (these false positives are annotated in Swiss-Prot as possessing signal peptides).

Three mouse proteins (GAPR1_MOUSE, TENR_MOUSE, VEGFD_MOUSE) containing extracellular domains apparently truly lack classical signal peptides (i.e. none of the signal prediction programs detected a signal sequence) and are probably subject to leaderless protein secretion [1]. Analysis of the remaining five entries revealed that they are truly abnormal and two of these (LY6G_MOUSE, MCPT3_MOUSE) are also annotated as fragments in Swiss-Prot.

C209C_MOUSE lacks both signal peptide and signal anchor sequences, whereas all of the closely related entries are type II transmembrane proteins. A corrected sequence of this protein could be predicted by targeted search of mouse genomic and EST sequences (Figure 2).

NOE2_MOUSE also lacks a signal peptide, whereas the rat ortholog (NP_001015017) and a different isoform of this mouse protein (EDL25126) do possess a signal sequence. TMPS7_MOUSE lacks a transmembrane helix: it is an N-terminally truncated version of mouse matriptase 3 (AAY66996).

In the case of *Rattus norvegicus* proteins MisPred identified 18 proteins as suspicious. One of these proteins, TENR_RAT, containing extracellular domains apparently truly lacks classical signal peptides (i.e. none of the signal prediction programs detected a signal sequence) and is probably subject to leaderless protein secretion [1].

Fourteen proteins ANGP2_RAT, CADH4_RAT, CATG_RAT, CO5_RAT, CO8B_RAT, COBA1_RAT, FA9_RAT, GDF11_RAT, KLK10_RAT, KLK3_RAT, MUC2L_RAT, NID1_RAT, PDGFB_RAT, SFRP1_RAT proved to be true positives (also annotated as fragments in Swiss-Prot). CSF2_RAT, MIP2A_RAT are also fragments of larger precursors although not annotated as such in Swiss-Prot.

Analysis of *Gallus gallus* Swiss-Prot proteins by MisPred identified 22 as being suspicious. Analysis of these proteins revealed that 19 proteins are in fact abnormal, and 12 of these are also annotated as fragments by Swiss-Prot. The remaining seven entries (SECR_CHICK, PYY_CHICK, IGF2_CHICK, NMU_CHICK, AMP1_CHICK, RNL2_CHICK, IPK1L_CHICK) are also fragments of larger precursors but are not annotated as such by Swiss-Prot.

In the case of *Danio rerio* proteins MisPred identified four proteins as suspicious. One protein, OSTC_DANRE, is truly abnormal and is also annotated as a fragment by Swiss-Prot.

Analysis of *Caenorhabditis elegans* Swiss-Prot proteins by MisPred identified 9 proteins as being suspicious. Detailed analyses of these sequences revealed that LAML1_CAEEL, NAS10_CAEEL, YP95_CAEEL, YOW6_CAEEL, NAS13_CAEEL, CUBN_CAEEL, YL54_CAEEL are true positives: they lack secretory signal peptides and transmembrane helices, although their close homologs contain such sequence signals. (In the majority of these cases the mispredicted sequences could be corrected by targeted search of genomic sequences using close homologs as queries).

NUCG_CAEEL is a mitochondrial protein that truly lacks a eukaryotic-type secretory signal peptide although it contains an extracellular Endonuclease_NS domain, cautioning that this Pfam-A domain family is not obligatory extracellular.

YL15_CAEEL, containing an extracellular Kunitz_BPTI domain, was also identified as suspicious since it lacked signal peptide and transmembrane sequences. Blast searches have confirmed that this is a mispredicted chimeric protein, created through *in silico* joining of two tandem genes located on *Caenorhabditis elegans* chromosome X. The Homeobox-containing region is derived from a gene closely related to the gene encoding the nuclear protein HM07_CAEEL, whereas the Kunitz_BPTI-containing region is derived from the *C. elegans* ortholog of an extracellular protein of *C. briggsae*, Q619J1_CAEBR. The structures of the constituent genes could be predicted using the sequences of these homologs (Figure 3). (Note that the co-occurrence of a predominantly nuclear Homeobox domain and an obligatory extracellular Kunitz_BPTI domain also violates the rule underlying Conflict 3).

Analysis of *Drosophila melanogaster* proteins identified 5 entries as suspicious. Two of these (CHIT1_DROME, CHIT3_DROME) are fragments of longer proteins possessing signal peptides. PGPLE_DROME appears to be a false positive that truly lacks a signal peptide, probably because its extracellular Amidase_2 domain is not necessarily associated with secretory signal peptides.

Conflict 4

The MisPred routine for Conflict 4 revealed that of the 15638 human proteins 6973 contained at least one domain belonging to Pfam-A domain families suitable for the study of domain integrity [see Additional file 6]. Ten of these proteins were identified by MisPred as suspicious. Four proteins contained an incomplete domain, whereas the remaining six proteins

were false positives due to failure to detect the full-length Pfam-A domain or a false hit with a Pfam-A domain. Three of the four abnormal proteins (HB2T_HUMAN, POK9_HUMAN, M4A4E_HUMAN) were also annotated as fragments by Swiss-Prot, one protein (VHLL_HUMAN) identified by MisPred as abnormal was not annotated as a fragment in Swiss-Prot. VHLL_HUMAN is closely related to VHL_HUMAN but its C-terminal VHL domain is truncated [2].

Analysis of mouse Swiss-Prot proteins has identified three proteins as suspicious. Only one of these proved to be truly abnormal: MYO1A_MOUSE contains a truncated Myosin_TH1 domain (the protein is also annotated as a fragment by Swiss-Prot).

Analysis of rat Swiss-Prot proteins with the MisPred routine for Conflict 4 has identified 14 proteins that contain a domain of abnormal size. 13 of these proteins are fragments and are also annotated as such by Swiss-Prot. EPHA5_RAT contains a C-terminally truncated SAM_1 domain, although not annotated as a fragment by Swiss-Prot. It is noteworthy that orthologs from mouse, human and chicken contain an intact SAM_1 domain. The sequence could be corrected with targeted search of the rat genome using the sequences of the full-length orthologs (Figure 4).

Analysis of chicken Swiss-Prot proteins has identified 8 proteins as abnormal, all of which were also annotated as fragments by Swiss-Prot.

Only one of the *Danio rerio* Swiss-Prot proteins was identified as abnormal by Conflict 4. DHH_BRARE, containing a truncated HH_signal domain, is a fragment of the full-length protein; it is also annotated as a fragment by Swiss-Prot.

Analyses of *C. elegans* Swiss-Prot proteins have identified two proteins that contain a domain of deviant size. YQS2_CAEEL, containing an N-terminally truncated Patched domain, is actually a fragment of PTC1_CAEEL, although not annotated as a fragment by Swiss-Prot. (The latter protein contains a full-length Patched domain.) PME6_CAEEL, containing a truncated

WGR domain, is a fragment of PME1_CAEEL, the latter protein contains a full-length WGR domain. (Note that PME6_CAEEL in release 45.5 has been deleted.)

None of the *Drosophila melanogaster* Swiss-Prot entries these proteins were identified by the MisPred routine for Conflict 4.

Conflict 5

MisPred identified five human Swiss-Prot entries as suspected to be translated from chimeras of genes located on different chromosomes. Two of these, CR030_HUMAN and MED12_HUMAN, were true chimeras derived from unrelated genes located on different chromosomes. In the case of MED12_HUMAN, the N-terminal 22 residues are encoded by chromosome 1, whereas residues 23-2198 are encoded by chromosome X. (It should be pointed out that the sequence of MED12_HUMAN was determined from a cDNA derived from cervix carcinoma).

In the case of CR030_HUMAN, the N-terminal 1-563 residues of this hypothetical protein are encoded by chromosome 18, whereas residues 564-583 are derived from *Homo sapiens* asparagine-linked glycosylation 12 homolog (ALG12), encoded on chromosome 22.

The remaining three sequences, CYB_HUMAN, COX1_HUMAN and APOA_HUMAN, proved to be false positives. CYB_HUMAN and COX1_HUMAN are encoded by the human mitochondrial genome, however, in the BLAT search they did not give a perfect match with this genome because the BLAT search uses the nuclear genetic code (not the mitochondrial genetic code). On the other hand, these protein sequences gave shorter, perfect matches with nuclear chromosomes containing sequences related to these genes. To eliminate this source of false positives, in all subsequent studies of Conflict 5 we used a modified BLAT protocol that removed entries encoded by mitochondrial genes (see Materials and Methods).

Analysis of APOA_HUMAN illustrates the point that the primary reason why some sequences may be incorrectly identified by MisPred as chimeras is that there are closely related genes (gene segments) on different chromosomes and these may give short matches with the query sequence. The gene for APOA_HUMAN is located on chromosome 6 but a perfect match with its first 16 residues is also given by chromosome 2. Despite this limitation of our approach (that short perfect matches may be given by different chromosomes), the percentage of false positives is very low (0.02%).

MisPred identified 5 rat proteins as suspected to be chimeras. Two of these sequences, TRPC3_RAT and SYJ2B_RAT, were true chimeras, derived from unrelated genes located on different chromosomes.

BLAT searches identified 18 zebrafish proteins as suspected to be chimeras. One sequence proved to be a false positive since it gave a short perfect match with another chromosome. The remaining zebrafish sequences (ACC4A_DANRE, AN13C_DANRE, BCL7B_DANRE, CC104_DANRE, CHMA1A_DANRE, EXT1B_DANRE, FGFR2_DANRE, JAG1A_DANRE, GP175_DANRE, KC15B_DANRE, LDHA_DANRE, PA24A_DANRE, PALD_DANRE, TC1D1_DANRE, UBE2F_DANRE, UBTD1_DANRE, WN10A_DANRE) appeared to be truly “chimeric” inasmuch as they are derived from two or more genomic regions assigned to different chromosomes. It should be noted, however, that none of these proteins are chimeras of unrelated genes, they all encode full-length sequences typical of the given protein family.

The fact that the proportion of suspicious proteins (i.e. chimeras derived from genomic regions assigned to different chromosomes) is significantly higher in zebrafish (1.4%) than in the other vertebrates (0.00-0.08%) might be explained by assuming that, for some reason, formation of chimeric transcripts of homologous genes is more likely to occur in zebrafish than in other

vertebrates. A possible support for this explanation might be found in the fact that the common ancestor of ray-finned fishes experienced a whole genome duplication, therefore fish genomes usually have duplicates of single orthologs found in other vertebrates [3-5]. In principle, the higher number of close homologs may increase the chance of interchromosomal homologous recombination (or transchromosomal transcription) and the subsequent formation of chimeric transcripts and chimeric proteins.

An implicit prediction of this explanation, however, is that transcripts of trans-chromosomal chimeras are likely to be rare, whereas normal transcripts of the parent genes are expected to be more abundant. Our observation that ESTs corresponding to the "chimeric" transcripts were found, but the predicted "non-chimeric" transcripts were missing from EST databases (data not shown) suggests that this explanation of the high rate of true positives in zebrafish may not be valid.

An alternative explanation is that the chromosomal assignment of contigs encoding these zebrafish genes may not always be correct. It is noteworthy in this respect that the chromosomal matches of these chimeric proteins are sometimes (e.g. AN13C_DANRE, CHMA1A_DANRE, LDHA_DANRE) found at the termini of short zebrafish contigs, the termini of the different contigs coincide with the boundaries of the apparent chimera formation.

MisPred identified no mouse, chicken, worm or fruitfly Swiss-Prot proteins as suspected to be chimeras.

Comments on mispredicted EnsEMBL entries identified by MisPred routines as suspicious

Conflict 2

Mispredicted entries include the rat proteins ENSRNOP00000019138 (a Notch protein homolog) and ENSRNOP00000036086 (related to Angiopoietin-1 receptor precursor, TIE-2), the opossum EPH receptor B1 homologs ENSMODP00000021175, ENSMODP00000031819, ENSMODP00000032387, ENSMODP00000032388, ENSMODP00000032389, ENSMODP00000033795, ENSMODP00000033797, ENSMODP00000033802, ENSMODP00000033804, ENSMODP00000035096, ENSMODP00000035099, ENSMODP00000036530, ENSMODP00000036533, ENSMODP00000036535, ENSMODP00000038060 and ENSMODP00000038066, and the chicken Notch-1 homolog ENSGALP00000003735. All of these proteins lack transmembrane helices, whereas their equivalents from the same or related species do possess transmembrane segments.

Similarly, the frog proteins ENSXETP00000020884, encoding an erbB-3 receptor tyrosine-protein kinase, ENSXETP00000037512, encoding a homolog of Notch 2 preprotein, ENSXETP00000038207, ENSXETP00000041110 and ENSXETP00000041112 encoding homologs of Eph receptor EphA4b, lack the transmembrane helix characteristic of orthologs of these transmembrane proteins. ENSXETP00000040601, which corresponds to the frog ortholog of Ephrin receptor A7, also lacks a typical transmembrane helix between its extracellular and intracellular domains. The missing transmembrane sequence could be corrected using frog EST sequences such as EL820950.

The *Danio rerio* protein ENSDARP00000000102 contains extracellular Fz and Kringle domains and a cytoplasmic Pkinase domain but lacks a transmembrane segment. This mispredicted protein is encoded by the same gene as unplugged isoform FL of *Danio rerio*

(AAT07679) that encodes a functional receptor tyrosine kinase (with intact transmembrane helix) related to skeletal muscle receptor tyrosine kinases.

The seven true positives of *Fugu rubripes* include NEWSINFRUP00000149801, a protein related to Receptor-type tyrosine-protein phosphatase kappa precursor, NEWSINFRUP00000165700 and NEWSINFRUP00000175769, proteins related to EPH receptor tyrosine kinases but lack transmembrane helices separating their extracellular and intracellular domains.

Analysis of the remaining four true positive pufferfish sequences has revealed that they lack transmembrane helices for a different reason: they probably arose through *in silico* fusion of distinct, tandem genes encoding extracellular and intracellular proteins. BLAST searches of the human genome with the pufferfish fusion proteins revealed that homologs of its constituent genes are usually not closely linked (either separated by numerous genes or located on different chromosomes) in the human genome.

For example, NEWSINFRUP00000146634 contains an extracellular IGFBP domain and an intracellular signaling PIP5K domain. BLAST searches revealed that the IGFBP domain is derived from an IGF binding protein 6 related gene, whereas the PIP5K domain is encoded by a phosphatidylinositol-4-phosphate-5-kinase related gene. BLAST searches of the human genome with this fusion protein revealed that orthologs of its constituent genes are separated by more than 4 Mb and numerous genes on chromosome 12 of the human genome.

NEWSINFRUP00000155548 contains extracellular CUB and Trypsin domains and an intracellular RhoGAP domain. BLAST searches revealed that the CUB and Trypsin domains are encoded by a trypsin-related gene, whereas the RhoGAP domain is encoded by a FAM13A1_v2 protein related gene. BLAST searches of the human genome with this fusion protein revealed that

homologs of its constituent genes are separated by more than 6 Mb and numerous genes on chromosome 4 of the human genome.

NEWSINFRUP00000161639 contains an extracellular VWD domain and an intracellular signaling Y_phosphatase domain. BLAST searches revealed that the VWD domain is encoded by an otogelin related gene, whereas the Y_phosphatase domain is encoded by protein tyrosine phosphatase receptor type Q related gene. BLAST searches of the human genome with this fusion protein revealed that orthologs of the constituent genes are closely linked (separated by less than 1 Mb) on chromosome 12 in the human genome.

NEWSINFRUP00000161730 contains extracellular PSI domain and intracellular LIM and SH3_1 domains. BLAST searches revealed that the PSI domain is encoded by Plexin domain-containing protein 1 precursor related gene, whereas the LIM and SH3_1 domains are encoded by LIM and SH3 protein 1 related gene. BLAST searches of the human genome with this fusion protein revealed that orthologs of the constituent genes (*LASP1* and *PLXD1*) are also linked (on chromosome 17) in the human genome.

Conflict 3

The MisPred analyses of EnsEMBL sequences for Conflict 3 have identified 1 human, 3 mouse, 3 rat, 0 opossum, 1 chicken, 0 frog, 2 pufferfish, 0 zebrafish, 0 sea squirt, 0 worm and 0 fruitfly EnsEMBL sequences as suspicious, i.e. they contain both extracellular and nuclear domains.

Human ENSP00000374642, mouse ENSMUSP00000023060, mouse ENSMUSP00000086712, mouse ENSMUSP00000086714, rat ENSRNOP00000004703, rat ENSRNOP00000022586, rat ENSRNOP00000052999, and chicken ENSGALP00000019866 were all found to contain extracellular Pentaxin and nuclear Chromo domains. BLAST searches

revealed that their Pentaxin domains are encoded by a neuronal pentraxin receptor related gene, whereas the Chromo domain is encoded by a Chromobox protein homolog 6 related gene.

The *Fugu rubripes* protein NEWSINFRUP00000161053 contains nuclear SNF2_N and Helicase_C domains (intracellular Ras domains) and extracellular Cadherin domains. BLAST searches revealed that the SNF2_N and Helicase_C domains are encoded by a homolog of DNA repair and recombination protein RAD54B (the Ras domain is encoded by a gene related to the GTP binding protein gene overexpressed in skeletal muscle) and the Cadherin domains are encoded by a Cadherin 17 related gene. BLAST searches of the human genome with this pufferfish fusion protein revealed that orthologs of the three constituent genes (*RAD54b*, *GEM* and *CDH17*) are also linked (on chromosome 8) in the human genome.

The *Fugu rubripes* protein NEWSINFRUP00000168199 contains nuclear zf-C3HC4 and zf-B_box domains (an intracellular SPRY domain) and an extracellular C1q domain. BLAST searches revealed that the zf-C3HC4, zf-B_box (and SPRY) domains are encoded by a RING finger protein-related gene, whereas the C1q domain is encoded by a cerebellin 1 precursor related gene. BLAST searches of the human genome with this fusion protein revealed that homologs of its constituent genes are not linked in the human genome.

Comments on erroneous GNOMON-predicted proteins identified by MisPred routines as suspicious

Conflict 2

Proteins XP_001372663, XP_001372858 and XP_001381008 contain both extracellular (SEA) and intracellular (C1_1, Pkinase, Pkinase_C) domains but lack transmembrane helices. BLAST searches have confirmed that these predicted protein sequences arose through the fusion of genes encoding a protein related to mucin 16 and a gene encoding a protein kinase C zeta type. BLAST searches of the human genome revealed that the constituent genes are located on different chromosomes (chromosome 19 and chromosome 1).

Protein XP_426670 contains an extracellular Insulin domain and intracellular WD40 domains, but no transmembrane domain. BLAST searches revealed that this predicted protein sequence arose through the fusion of tandem genes encoding a protein related to WD repeat protein and a relaxin 3 precursor related gene. BLAST searches of the human genome revealed that the constituent genes are located on different chromosomes (chromosome 1 and chromosome 19) in the human genome.

Protein XP_424494 contains an extracellular Somatomedin_B domain and intracellular RasGEF and DEP domains but no transmembrane segment. BLAST searches revealed that this predicted protein sequence arose through the fusion of tandem genes encoding a protein related to placental protein 11 and a Rap guanine nucleotide exchange factor (GEF) 3 related gene. BLAST searches of the human genome with this chicken fusion protein revealed that the constituent genes are also closely linked (on chromosome 12) in the human genome. The fact that no cDNAs or ESTs were found that span these constituent genes, suggests that they are distinct genes.

XP_001337907 was found to contain extracellular Lectin_C and intracellular kinesin domains. BLAST searches revealed that the protein arose through fusion of a kinesin-related gene and a novel C-type lectin domain containing protein.

XP_001340361 contains extracellular SRCR domains and an intracellular CH domain. This protein apparently arose through fusion of a Scavenger receptor cysteine-rich type 1 protein-related gene and a gene encoding a filamin-related protein. BLAST searches revealed that the constituent genes are located on different chromosomes (chromosome 12 and chromosome X) in the human genome.

XP_696912 contains an extracellular Laminin_G_2 domain and an intracellular Exo_endo_phos domain. This protein apparently arose through fusion of a collagen XXVII proalpha 1 chain precursor related gene and an inositol polyphosphate-5-phosphatase E related gene. BLAST searches of the human genome with this zebrafish fusion protein revealed that the constituent genes are on chromosome 9 in the human genome separated by more than 22 Mb (and numerous genes).

XP_001343892 contains extracellular EGF domains and an intracellular VPS9 domain. This protein apparently arose through fusion of a NOTCH related gene and a Rab5-activating protein 6 related gene. BLAST searches of the human genome with this zebrafish fusion protein revealed that the constituent genes are on chromosome 9 in the human genome, separated by 10 Mb (and numerous genes).

XP_690248 contains two extracellular CUB domains and an intracellular CH domain. BLAST searches revealed that the CUB domains are encoded by a procollagen C-endopeptidase enhancer 2 related gene, whereas the intracellular CH domain is encoded by the plastin 1 gene. BLAST searches of the human genome with this zebrafish fusion protein revealed that the constituent genes are linked on chromosome 3 in the human genome separated by a gene for

transient receptor potential cation channel, subfamily C, member 1. EST searches provided no evidence for cotranscription of these genes.

XP_001334415 contains a C-terminal extracellular OLF domain and an N-terminal intracellular signaling WH1 domain. BLAST searches revealed that the OLF domain is encoded by latrophilin-2 related gene, the WH1 domain is encoded by Wiskott-Aldrich syndrome-related gene, whereas the middle of the protein is related to retroviral polyproteins. BLAST searches of the human genome with this zebrafish fusion protein revealed that the constituent genes are on two chromosomes in the human genome (Wiskott-Aldrich syndrome-related gene is on chromosome 7, latrophilin-2 related gene is on chromosome 19).

Conflict 3

XP_001375906 was found to contain both extracellular (MHC_I, C1_set) and nuclear (Pou, Homeobox) domains. BLAST searches have confirmed that this predicted protein sequence arose through the fusion of tandem genes encoding the opossum ortholog of the POU domain, class 5, transcription factor, PO5F1_PIG, and a gene encoding a protein related to MHC class I antigens. BLAST searches of the human genome with this opossum fusion protein revealed that the constituent genes are on different chromosomes (chromosome 12 and chromosome 6) in the human genome.

XP_001380072 was found to contain three extracellular WAP domains and a nuclear histone domain. BLAST searches revealed that this predicted protein arose through fusion of a gene encoding a whey acidic protein-like protein and a gene for Histone H2A.l. BLAST searches of the human genome with this opossum fusion protein revealed that homologs of the constituent genes are located on different chromosomes in the human genome.

XP_425476 contains an extracellular Pentaxin and a nuclear Chromo domain. BLAST searches revealed that the Pentaxin domain is encoded by a neuronal pentraxin receptor related gene, whereas the Chromo domain is encoded by a Chromobox protein homolog 6 related protein. As discussed above (cf. EnsEMBL sequences) homologs of the constituent genes are closely linked in human and other mammalian species.

XP_686306 contains an extracellular Lectin_C and a nuclear zf-B_box domain. BLAST searches revealed that the Lectin_C domain is encoded by gene related to serum lectin isoform 5 precursor of *Salmo salar*, whereas the zf-B_box domain is encoded by a Tripartite motif-containing protein 2 related protein. BLAST search provided no evidence for the linkage of related genes in the human genome.

XP_001337208 contains extracellular SRCR domain and nuclear SCAN and zf-CCHC domains. BLAST searches revealed that the SCAN and zf-CCHC domains are encoded by a gene related to LReO_3 of *Oryzias latipes*, whereas the SRCR domain is most closely related to those of neurotrypsin. BLAST searches revealed that homologs of the constituent genes are not linked in the human genome.

XP_001338835 contains an extracellular A4_EXTRA domain and nuclear zf-C2H2 domains. BLAST searches revealed that the zf-C2H2 domains are encoded by the gene encoding the zebrafish protein CAM13007, similar to vertebrate PR domain containing 10 (PRDM10). The A4 EXTRA domain is encoded by the gene encoding the zebrafish protein CAM13008, a novel protein similar to vertebrate amyloid beta (A4) precursor-like protein 2 (APLP2). BLAST searches of the human genome with this fusion protein revealed that the constituent genes are closely linked (separated by less than 1 Mb) on chromosome 11 in the human genome. Note, however, that no ESTs were found that would support co-transcription of these genes in vertebrate genomes.

XP_001332478 contains extracellular Ldl_recept_a and Ldl_recept_b domains and a nuclear zf-C4 domain. BLAST searches revealed that the zf-C4 domain is encoded by a gene related to hepatocyte nuclear factor 4 alpha isoform, whereas the Ldl_recept_a and Ldl_recept_b domains are most closely related to low-density lipoprotein receptor-related protein 1 precursor. BLAST searches revealed that orthologs of the constituent genes are located on different chromosomes (chromosome 20 and chromosome 12) in the human genome.

Comments on EnsEMBL and NCBI entries predicted for the same set of genes

Conflict 1

MisPred analyses of EnsEMBL and NCBI entries predicted for the same set of genes have revealed that the proportion of suspicious sequences detectable by the routine for Conflict 1 are quite similar for EnsEMBL- and GNOMON-predictions in the case of human (15.65% vs. 23.02%), opossum (20.15% vs. 17.45%) and chicken (32.75% vs. 28.39%) genes. In the case of zebrafish genes, however, the proportion of suspicious proteins was found to be lower in the case of GNOMON-predicted proteins (21.29%) than EnsEMBL entries (36.86%).

Conflict 2

MisPred analyses have returned no human EnsEMBL or GNOMON-predicted sequences for Conflict 2 as being erroneous. In the case of chicken, MisPred analyses have returned 1 GNOMON protein but no EnsEMBL proteins as suspicious. The GNOMON-predicted protein was a false positive due to lack of detection of its transmembrane helix by TMHMM.

In the case of opossum no EnsEMBL sequences but four GNOMON-predicted sequences were identified by MisPred as suspicious. Two of the latter were false positives due to lack of detection of their transmembrane helices by TMHMM, but two were truly erroneous: they arose through *in silico* fusion of genes encoding extracellular and intracellular proteins.

In the case of *Danio rerio*, MisPred analysis identified five EnsEMBL sequences and six GNOMON-predicted sequences as being suspicious. Four of the EnsEMBL entries and four of the GNOMON-predicted entries were false positives due to failure of TMHMM to detect their transmembrane helices or false Pfam-A domain hits.

One truly erroneous EnsEMBL sequence, ENSDARP00000000102, similar to Tyrosine-protein kinase transmembrane receptor ROR1 precursor, contains extracellular Fz and Kringle domains and an intracellular Pkinase domain but lacks the transmembrane segment separating its constituent extracellular and intracellular domains. It should be noted that the corresponding GNOMON-predicted sequence, XP_001332675, does contain the expected transmembrane helix.

The remaining two truly erroneous GNOMON-predicted proteins of *Danio rerio* apparently arose through the *in silico* fusion of genes encoding extracellular and intracellular proteins. XP_690248 arose through fusion of a procollagen C-endopeptidase enhancer 2 related gene and a plastin 1 related gene. Similarly, XP_001334415 probably arose through fusion of a latrophilin-2 related gene and a Wiskott-Aldrich syndrome related gene.

In summary, the results of the MisPred analyses of human, opossum, chicken and zebrafish sequences suggest that the GNOMON and EnsEMBL pipelines are characterized by similarly low proportion of error detectable by the routine for Conflict 2. It should be pointed out, however, that GNOMON appears to be slightly more prone to erroneous fusion of genes.

Conflict 3

MisPred analysis has found no human or chicken EnsEMBL or GNOMON-predicted proteins containing both extracellular and nuclear Pfam-A domains.

In the case of opossum EnsEMBL proteins no sequence was found to contain both extracellular and nuclear Pfam-A domains, whereas in the case of opossum GNOMON-predicted proteins XP_001375906 contains an extracellular MHC-I domain and intracellular signaling Pou and Homeobox domains. This protein probably arose through the *in silico* fusion of Hereditary hemochromatosis protein homolog precursor related gene and a POU domain class 5 transcription factor 1-like protein related gene.

MisPred analysis of zebrafish entries has found no EnsEMBL proteins containing both extracellular and nuclear Pfam-A domains, whereas two GNOMON-predicted proteins were identified as suspicious by MisPred. One of these sequences was a false positive, due to a false Pfam-A domain hit. Protein XP_001332478 was found to contain extracellular Ldl_recept_a and Ldl_recept_b domains and an intracellular signaling zf-C4 domain. This sequence probably arose through the *in silico* fusion of a Hnf4a protein related gene and a gene closely related to low-density lipoprotein receptor-related protein 1.

In summary, MisPred analysis of human, opossum, chicken and zebrafish sequences predicted by GNOMON and EnsEMBL pipelines has revealed that the rates of error detectable by Conflict 3 are characterized by similarly very low values for all of these species. It should be pointed out, however, that GNOMON appears to be slightly more prone to the erroneous fusion of tandem genes.

Conflict 4

MisPred analyses of EnsEMBL and GNOMON entries predicted for the same set of genes have revealed that the proportion of suspicious sequences predicted by the two pipelines are not drastically different for human (6.65% vs. 5.18%) and chicken (9.70% vs. 13.87%) genes, but there are somewhat greater differences regarding opossum (3.19% vs. 1.14%) and zebrafish (7.68% vs. 14.57%) genes.

Conflict 5

MisPred identified none of the human EnsEMBL- and only one of the GNOMON-predicted sequences as being encoded by two genomic segments located on different chromosomes. As discussed above XP_001128605 (now replaced by the nonchimeric NP_001035225) is a chimera of genomic regions located on chromosomes 2 and 7.

In the case of zebrafish, Mispred identified none of the EnsEMBL- and five of the GNOMON-predicted sequences as suspicious. Analyses of the latter sequences have shown that none of them are truly chimeric: they had >90 % full-length match with chromosome regions designated as NA_random or UN_random.

Part 2. Detailed description of the detection of suspicious proteins by Conflict 4

Identification of Pfam-A families suitable for the study of the domain integrity

In order to identify the Pfam-A domain families that have a well-defined, conserved sequence length range (and are thus suitable for the study of domain integrity), we selected only those families whose members do not deviate from the average size by more than 2 SD values in the high quality Swiss-Prot database (version 48.9).

First, we calculated the average length and standard deviation for all Pfam-A domain families. For this calculation we used the seed alignments provided by Pfam (Pfam Release 19.0) since the sequences included in these alignments are usually full-length representatives of the Pfam-A domain families. Pfam Release 19.0 was downloaded from http://www.sanger.ac.uk/Software/Pfam/index.shtml.

In the next step we analyzed Pfam-A domains in Swiss-Prot sequences and selected those presumably incomplete Swiss-Prot domains whose beginning or end was missing their first or last >5 positions in the full alignments of the domain families, and whose lengths differed by at least 2 standard deviations from the Pfam-A seed averages. To check if these domains are genuinely incomplete or their domain boundaries were incorrectly defined by Pfam we ran a blastp search with the Swiss-Prot proteins containing the incomplete domains as queries against the full set of the Pfam-A domains to identify the missing parts of the domains. If the length of the corrected domain was inside the conserved length range (seed average ±2 SD) then the domain was considered to be complete.

Pfam-A domain families were deemed to be suitable for the study of domain integrity if they have either never been incomplete in Swiss-Prot proteins or all members of the family could be completed. Based on these criteria, about 90% of the Pfam-A domain families in the Swiss-

Prot proteins proved to have a well-defined, conserved length range and so can be used to identify mispredicted or abnormal proteins containing domains of deviant length. Furthermore, we also found that many Pfam-A domains that appear fragments at first sight in the seed alignments can be easily amended into full-length domains by comparing the vicinity of these domain fragments to homologous full-length Pfam-A domains. Of the 2752 Pfam-A domain families present in human Swiss-Prot proteins 2529 families, of the 2779 Pfam-A domain families present in vertebrate Swiss-Prot proteins 2515; of the 3559 Pfam-A domain families present in metazoa+fungi Swiss-Prot proteins 3010 families appeared to be suitable for the study of domain integrity.

Interestingly, a survey of the Pfam-A families that most frequently violate domain integrity revealed that they are usually found in numerous tandem copies in proteins (e.g. collagen triple helix repeat, Ankyrin repeat, Spectrin repeat, Plectin repeat, Tetratricopeptide repeat, cadherin domains, fibronectin type III domain, etc.) (see Supplementary table 1). The presence of the collagen triple helix repeat in this table simply reflects the fact that the dogma of a narrow domain-size range is not valid for fibrous proteins such as collagens.

In order to avoid artificial split-up of domain matches by blastp, we did not mask low complexity regions. However, in some instances this resulted in partial matches of two domains belonging to different Pfam-A families, therefore we removed these matching Pfam-A families from the set of the reliable Pfam-A families.

When we compared the domains of the 'reliable' Pfam-A families to one another we identified several pairwise matches with high significance and pairwise similarity (>60% identity) where the two domains belong to different Pfam-A families (see Supplementary table 2). Many of these Pfam-A domains match one another with high similarity because one domain is embedded in another. For example, the most frequently co-occurring pairs are the Fibronectin

type II domains, fn2 (PF00040) and peptidase_M10 (PF00413) domains, where the PF00040 was found to be embedded in a PF00413 domain 766 times (in gelatinases). As overlapping domains would cause ambiguity in the filtering of deviant domains, we eliminated the longer Pfam-A families that encompass the shorter ones. Another reason why members of different Pfam-A families may match one another with high similarity is because they belong to different but closely related Pfam families. For example, both bZIP_2 and bZIP_1 belong to the bZIP-like leucine zipper clan, both Arm and HEAT belong to the Armadillo repeat superfamily clan, both LRR_1 and LRR_2 belong to the Leucine Rich Repeat clan, both APH and Choline_kinase belong to the Protein kinase superfamily clan (see Supplementary table 2). To avoid ambiguity in the filtering of deviant domains, we eliminated both Pfam-A families from the list of domains suitable for the filtering task.

Using this approach we created databases of human, vertebrate and metazoa+fungi Swiss-Prot domain sequences belonging to Pfam-A families suitable for the study of domain integrity. The list of Pfam-A domain families suitable for the study of domain integrity is deposited in Additional file 6.

Identification of cases of violation of the domain integrity

In order to set a proper similarity threshold for the domain searches above which most partial matches would be eliminated we ran an all-against-all blastp search for the domains of all reliable Pfam-A families (i.e. whose members were never incomplete or where all could be repaired). The results for the human Sushi domains are shown in Supplementary figure 1 where the relative lengths (shorter/longer) of any two matching domains are plotted as a function of their percentage identity. As a contrast we also show the results of an all-against-all blastp search for all human Cadherin domains (see Supplementary figure 2). Unlike the conserved-length Sushi domains, the relative lengths of the matches among the Cadherin domains vary greatly even

above 60% identity, the threshold suggested above. It must be noted that as Cadherin proved to be a Pfam-A family of irregular type where not all of the incomplete members could be repaired; this domain type was already disqualified as a filter to search for incorrectly defined domains (see above).

After running an all-against-all blastp search for all domains we were able to establish the reliability of the approach as a function of the required sequence similarity between any two matching domains of the same type. We found that using a cut-off value of 60% sequence identity of two domain sequences eliminated at least 99% of partial matches, thus reducing the number of false positives to below 1%, i.e. this is an appropriate cut-off value for pairwise domain comparisons to filter for proteins containing fragment domains.

We ran a blastp search with the current set of proteins as queries against the appropriate reliable Swiss-Prot domain sequences, and selected those partial domain matches which share over 60% identity with the query sequence, with an E-value $< 1e^{-5}$, and differ by at least 40% in length. The protein sequences containing these domains with deviant lengths were identified as suspicious.

Size-distribution of gaps in pairwise matches of Pfam-A domains

We calculated the longest continuous gap for each pairwise domain match with > 60% sequence identity. The longest gap (indel) for any pairwise match with >60% sequence identity between any two domains belonging to a reliable Pfam-A family was 51, whereas between any two domains belonging to an unreliable Pfam-A family it was 52. The frequency distribution of the gaps by gap-size for the two sets of domains is shown in Supplementary figure 3.

It is clear from this figure that the data closely follow straight lines in double-logarithmic plots for both the reliable and the unreliable domain families. What is more, there is no significant difference between the two slopes. In other words, gap size distribution follows a

power law for both groups, i.e. the shortest gaps are the most abundant, whereas longer gaps are increasingly less frequent. This type of distribution is consistent with (and supports) the dogma on which MisPred routine 4 is based: insertion/deletion of longer segments are increasingly less likely to be compatible with the viability of a protein fold.

References

1. Bendtsen JD, Jensen LJ, Blom N, Von Heijne G, Brunak S: **Feature-based prediction of non-classical and leaderless protein secretion.** *Protein Eng Des Sel.* 2004, **17**:349-56.
2. Qi H, Gervais ML, Li W, DeCaprio JA, Challis JR, Ohh M: **Molecular cloning and characterization of the von Hippel-Lindau-like protein.** *Mol Cancer Res.* 2004, **2**:43-52.
3. Robinson-Rechavi M, Marchand O, Escriva H, Bardet PL, Zelus D, Hughes S, Laudet V: **Euteleost fish genomes are characterized by expansion of gene families.** *Genome Res.* 2001, **11**:781-8.
4. Taylor JS, Braasch I, Frickey T, Meyer A, Van de Peer Y: **Genome duplication, a trait shared by 22000 species of ray-finned fish.** *Genome Res.* 2003, **13**:382-90.
5. Kasahara M, Naruse K, Sasaki S, Nakatani Y, Qu W, Ahsan B, Yamada T, Nagayasu Y, Doi K, Kasai Y, Jindo T, Kobayashi D, Shimada A, Toyoda A, Kuroki Y, Fujiyama A, Sasaki T, Shimizu A, Asakawa S, Shimizu N, Hashimoto S, Yang J, Lee Y, Matsushima K, Sugano S, Sakaizumi M, Narita T, Ohishi K, Haga S, Ohta F, Nomoto H, Nogata K, Morishita T, Endo T, Shin-I T, Takeda H, Morishita S, Kohara Y: **The medaka draft genome and insights into vertebrate genome evolution.** *Nature* 2007, **447**:714-9.

Supplementary table 1. Pfam-A families that most frequently violate domain integrity in human Swiss-Prot proteins.

Pfam ID	Number of copies in human Swiss-Prot entries	Number of deviant family members	Percent of deviant family members	Number of deviant family members that could be corrected	Percent of deviant family members that could be corrected	Number of deviant family members that could not be corrected	Percent of deviant family members that could not be corrected	Average length	Standard deviation	Description
PF01391	487	192	39	185	96	7	4	60	0	Collagen triple helix repeat (20 copies)
PF00023	717	107	15	30	28	77	72	33,48	2,35	Ankyrin repeat
PF00028	630	97	15	90	93	7	7	94,53	4,81	Cadherin domain
PF00400	688	54	8	30	56	24	44	39,23	2,35	WD domain, G-beta repeat
PF00076	224	36	16	24	67	12	33	70,6	2,53	RNA recognition motif. (a.k.a. RRM, RBD, or RNP domain)
PF00435	315	32	10	19	59	13	41	105,85	2,71	Spectrin repeat
PF00041	402	27	7	16	59	11	41	84,44	5,47	Fibronectin type III domain
PF01344	171	24	14	12	50	12	50	47,22	3,06	Kelch motif
PF00053	167	24	14	12	50	12	50	50,67	5,07	Laminin EGF-like (Domains III and V)
PF00069	302	24	8	20	83	4	17	270,75	17,3	Protein kinase domain
PF00681	76	19	25	9	47	10	53	44,95	0,3	Plectin repeat
PF00595	202	18	9	10	56	8	44	84,1	5,01	PDZ domain (Also known as DHR or GLGF)
PF00018	151	16	11	13	81	3	9	56,11	1,09	SH3 domain
PF00071	107	14	13	11	79	3	21	165,21	6,36	Ras family
PF00036	303	13	4	4	31	9	69	28,89	0,56	EF hand
PF00063	38	12	32	2	17	10	83	678,32	17,52	Myosin head (motor domain)
PF00989	19	12	63	5	42	7	58	111,55	5,12	PAS fold
PF00067	57	12	21	8	67	4	33	459,12	11,37	Cytochrome P450
PF00324	21	12	57	12	100	0	0	467,64	23,01	Amino acid permease
PF00515	247	11	4	3	27	8	73	34	0,07	Tetratricopeptide repeat

Supplementary table 2. Nested and overlapping Pfam-A domain families.

Pfam1: Pfam ID of longer domain	Pfam name	Average size	Standard deviation	Pfam2: Pfam ID of shorter domain	Pfam name	Average size	Standard deviation	Number of times Pfam1 and Pfam2 overlap
PF00413	Peptidase_M10	164,59	31,97	PF00040	fn2	41,34	1,28	766
PF00406	ADK	177,13	12,95	PF05191	ADK_lid	36,06	0,32	97
PF00389	2-Hacid_dh	317,81	8,52	PF02826	2-Hacid_dh_C	178,23	5,4	89
PF02463	SMC_N	1033,91	307,22	PF06470	SMC_hinge	116,73	7,95	89
PF07716	bZIP_2	54,53	1,16	PF00170	bZIP_1	58,94	2,31	57
PF05221	AdoHcyase	457,38	21,27	PF00670	AdoHcyase_NAD	162,25	1,02	40
PF00478	IMPDH	446,25	54,33	PF00571	CBS	123,73	15,22	25
PF04857	CAF1	305,75	122,27	PF01424	R3H	56,08	1,64	25
PF00443	UCH	417,91	131,18	PF00627	UBA	40,49	0,82	18
PF05192	MutS_III	305,26	9,61	PF05190	MutS_IV	93,63	2,43	18
PF03917	GSH_synth_ATP	479,4	10,82	PF03199	GSH_synthase	104,55	2,16	17
PF02166	Androgen_recep	436,75	9,22	PF02155	GCR	374,5	2,12	12
PF01546	Peptidase_M20	322,42	24,47	PF07687	M20_dimer	115,06	21,64	9
PF02463	SMC_N	1033,91	307,22	PF04423	Rad50_zn_hook	55,21	1,18	9
PF01137	RTC	329,09	9,34	PF05189	RTC_insert	101,9	6,48	8
PF01193	RNA_pol_L	195,56	68,81	PF01000	RNA_pol_A_bac	120,27	8,7	8
PF00514	Arm	41,26	1,21	PF02985	HEAT	38,72	1,01	5
PF07723	LRR_2	25,92	1,03	PF00560	LRR_1	22,97	2,17	5
PF04998	RNA_pol_Rpb1_5	580,84	52,22	PF04990	RNA_pol_Rpb1_7	134,29	1,99	4
PF04998	RNA_pol_Rpb1_5	580,84	52,22	PF04992	RNA_pol_Rpb1_6	189,43	8,17	4
PF07690	MFS_1	368,24	29,28	PF05978	DUF895	156,27	1,42	4
PF03881	Fructosamin_kin	287,5	1	PF01636	APH	236,9	16,93	3
PF04931	DNA_pol_V	768,8	28,02	PF03066	Nucleoplasmin	175,68	31,07	3
PF01500	Keratin_B2	153,44	20,49	PF04360	Serglycin	150	0	2
PF01636	APH	236,9	16,93	PF01633	Choline_kinase	219,12	23,36	2
PF03344	Daxx	737,5	2,12	PF04849	HAP1_N	306	13,66	2
PF05308	DUF729	240,25	14,8	PF06346	Drf_FH1	161,5	10,28	1

Supplementary figure 1. Relative lengths of 254 matching human Sushi domains in pairwise blastp alignments. Relative length (calculated by dividing the length of the shorter with that of the longer member of the pairs aligned) is plotted as a function of percent sequence identity. Note that the length of matching domains does not deviate by more than 10% if sequence identity is >60%.

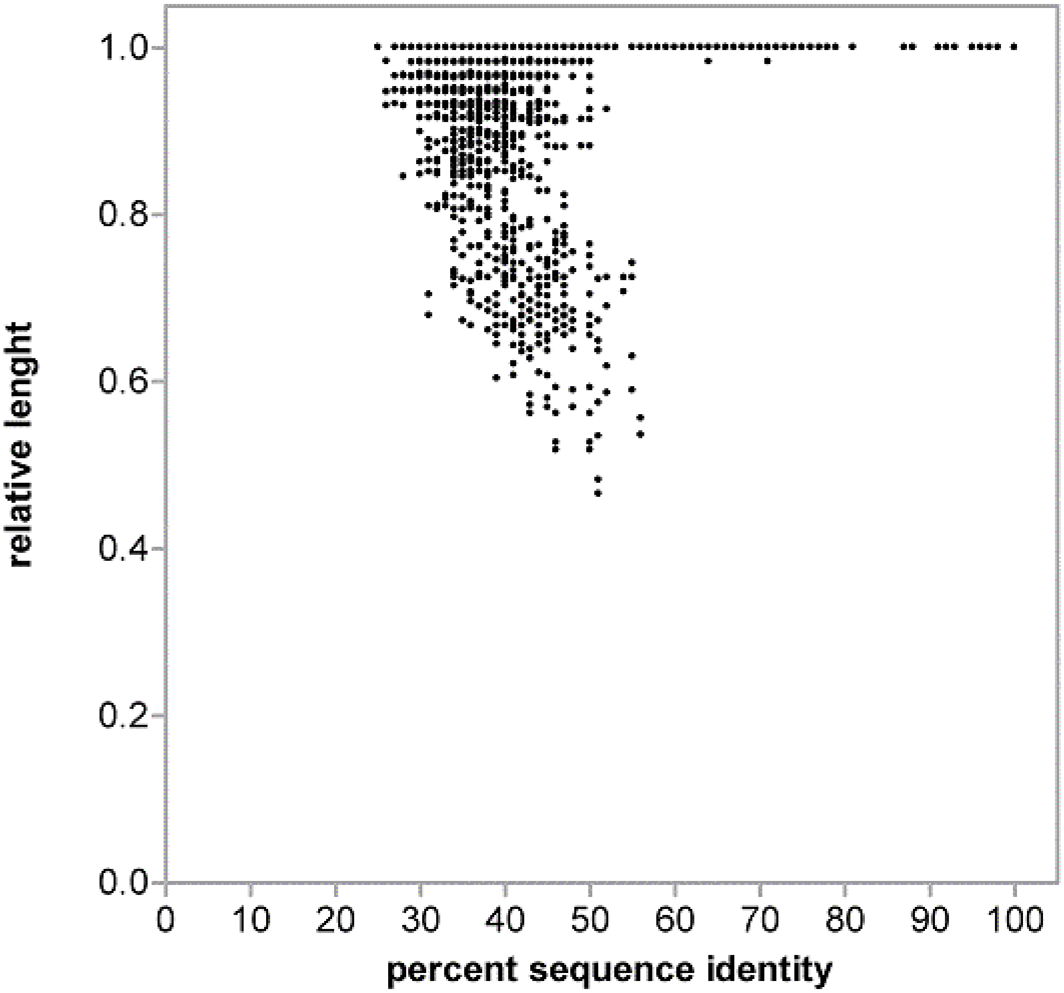

Supplementary figure 2. Relative lengths of 630 matching human Cadherin domains in pairwise blastp alignments. Relative length (calculated by dividing the length of the shorter with that of the longer member of the pairs aligned) is plotted as a function of percent sequence identity. Note that the length of matching domains deviates by more than 10% even if sequence identity is >95%. This domain family was not used for the filtering process described in this paper as not all its deviant domain representatives could be repaired.

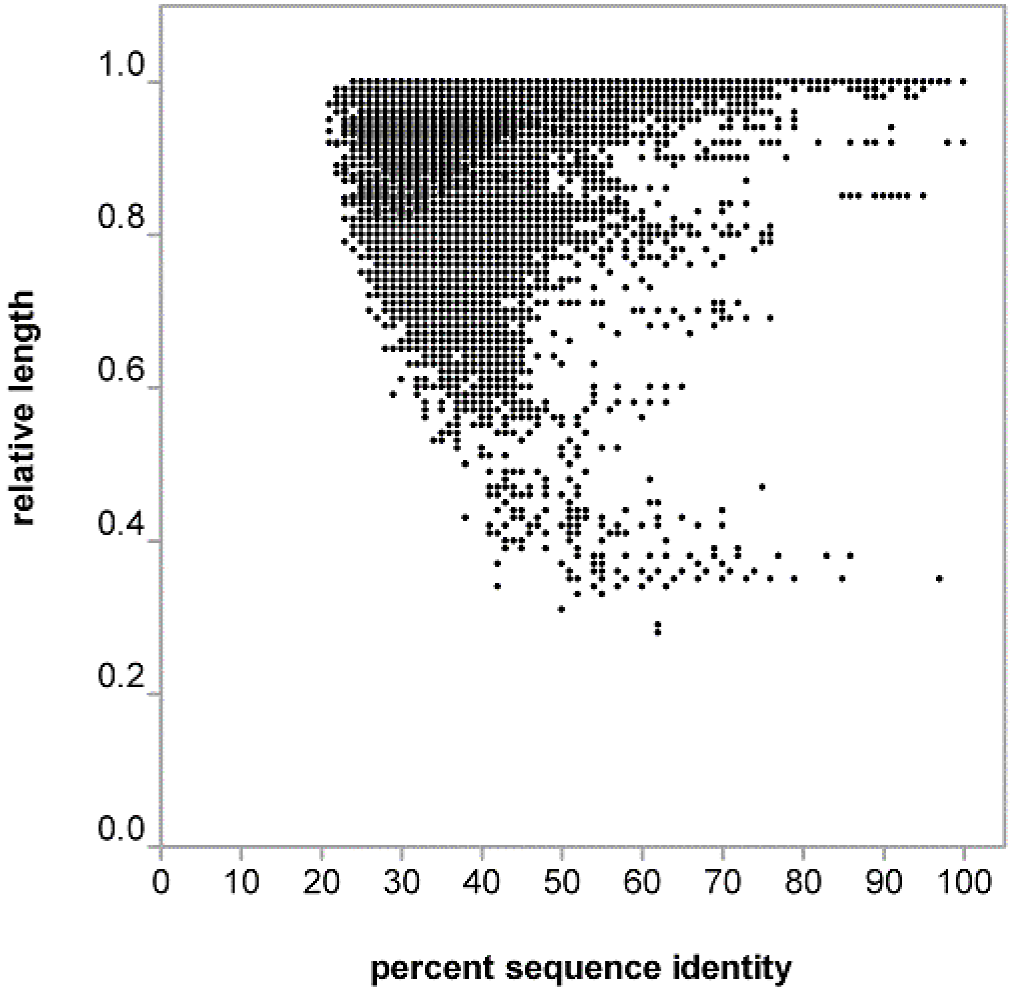

Supplementary figure 3. Frequency distribution of gaps for regular (▲) and deviant (○) domain families with respect to gap-size in pairwise domain alignments. The longest continuous gap was considered for any pairwise domain alignment with >60% percentage identity. The figure shows the frequency of gaps of a given size (y axis, log10 scale) as a function of the size of the gaps (x axis, log10 scale). Note that the longest recorded indel was 51 and 52 amino acids for regular and deviant domain families, respectively. The linear nature of the log-log representation of the data reveals a power-law distribution.

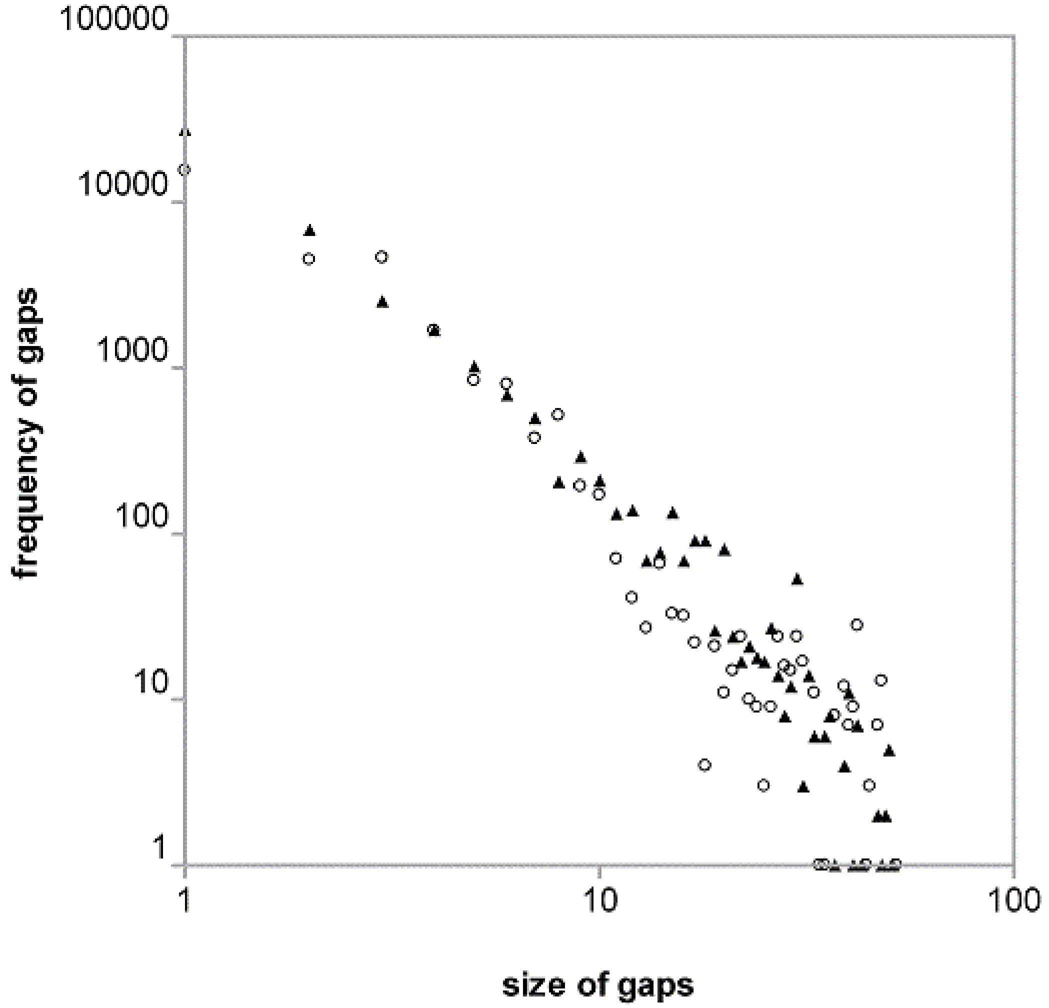

Supplement: Additional file 1 — Comments on entries identified by MisPred routines as suspicious and detailed description of Conflict 4. The file describes detailed analyses and comments of the entries identified as suspicious by the different MisPred routines, and contains the detailed description of the procedure of the identification of suspicious sequences by Conflict 4. [file 1471-2105-9-353-S1.pdf]
