# Supplementary material for: Identification and correction of abnormal, incomplete and mispredicted proteins in public databases
Source: BMC Bioinformatics. 2008 Aug 27;9:353. doi: 10.1186/1471-2105-9-353 (PMC2542381; doi:10.1186/1471-2105-9-353)
Supplement: Additional file 2 — List of erroneous Swiss-Prot sequences identified by MisPred. The file contains the list of erroneous Swiss-Prot sequences identified by MisPred. [file 1471-2105-9-353-S2.pdf]

Additional file 2. List of erroneous Swiss-Prot sequences identified by MisPred. Comments on these Swiss-Prot entries are found in Additional file 1, Part 1. Entries not annotated as fragments or chimeras in Swiss-Prot are in bold in the table.

Homo sapiens	**Conflict 1**	**Conflict 4**	**Conflict 5**
	ACROL_HUMAN	HB2T_HUMAN	**CR030_HUMAN**
	MUC5A_HUMAN	POK9_HUMAN	**MED12_HUMAN**
	POZP3_HUMAN	M4A4E_HUMAN	
	HB2G_HUMAN	**VHLL_HUMAN**	
	**LPLC4_HUMAN**		
Mus musculus	**Conflict 1**	**Conflict 4**	**Conflict 5**
	**C209C_MOUSE**	MYO1A_MOUSE	
	LYG6_MOUSE		
	MCPT3_MOUSE		
	**NOE2_MOUSE**		
	**TMPS7_MOUSE**		
Rattus norvegicus	**Conflict 1**	**Conflict 4**	**Conflict 5**
	ANGP2_RAT	ADA18_RAT	**TRPC3_RAT**
	CADH4_RAT	AL7A1_RAT	**SYJ2B_RAT**
	CATG_RAT	AMPD2_RAT	
	CO5_RAT	CO8B_RAT	
	CO8B_RAT	DUS7_RAT	
	COBA1_RAT	**EPHA5_RAT**	
	**CSF2_RAT**	E2F1_RAT	
	FA9_RAT	FER_RAT	
	GDF11_RAT	KITH_RAT	
	KLK10_RAT	MAAI_RAT	
	KLK3_RAT	PTN23_RAT	
	**MIP2A_RAT**	RPGF4_RAT	
	MUC2L_RAT	SFRP1_RAT	
	NID1_RAT	UCK2_RAT	
	PDGFB_RAT		
	SFRP1_RAT		
Gallus gallus	**Conflict 1**	**Conflict 4**	**Conflict 5**
	**SECR_CHICK**	ALDOC_CHICK	
	CO2A1_CHICK	AMPD1_CHICK	
	TGFB1_CHICK	AT2B1_CHICK	
	CATL_CHICK	FZD3_CHICK	
	FINC_CHICK	FZD6_CHICK	
	**PYY_CHICK**	HXA9_CHICK	
	**IGF2_CHICK**	LAMBV_CHICK	
	**NMU_CHICK**	S100G_CHICK	
	**AMP1_CHICK**		
	CFBL_CHICK		
	**RNL2_CHICK**		
	**IPK1L_CHICK**		
	LAMB1_CHICK		
	LAMBV_CHICK		
	FIBB_CHICK		
	SEM4D_CHICK		
	BMP2_CHICK		
	WNT1_CHICK		
	SORL_CHICK		
Danio rerio	**Conflict 1**	**Conflict 4**	**Conflict 5**
	OSTC_BRARE	DHH_BRARE	
Caenorhabditis elegans	**Conflict 1**	**Conflict 4**	**Conflict 5**
	**YOW6_CAEEL**	PME6_CAEEL	
	**YL54_CAEEL**	**YQS2_CAEEL**	
	**YL15_CAEEL**		
	**NAS10_CAEEL**		
	**LAML1_CAEEL**		
	**YP95_CAEEL**		
	**NAS13_CAEEL**		
	**CUBN_CAEEL**		
Drosophila melanogaster	**Conflict 1**	**Conflict 4**	**Conflict 5**

	**CHIT3_DROME**		
	**CHIT1_DROME**		
