# Supplementary material for: Identification and correction of abnormal, incomplete and mispredicted proteins in public databases
Source: BMC Bioinformatics. 2008 Aug 27;9:353. doi: 10.1186/1471-2105-9-353 (PMC2542381; doi:10.1186/1471-2105-9-353)
Supplement: Additional file 3 — List of extracellular Pfam-A domain families. The file contains the list of extracellular Pfam-A domain families. [file 1471-2105-9-353-S3.pdf]

Additional file 3. List of extracellular Pfam-A domain families. The table contains obligatory extracellular Pfam-A domain families which were used to predict subcellular localization of proteins. Our domain co-occurrence analyses of Metazoan UniProtKB entries have identified 166 obligatory extracellular Pfam-A domain families, the majority of which are also identified as such in the SMART database.

**Description**	**SMART name**	**Pfam name**	**Pfam ID**
Amyloid A4	A4_EXTRA	A4_EXTRA	PF02177
Plant lipid transfer protein / seed storage protein / trypsin-alpha amylase inhibitor domain family	AAI	Tryp_alpha_amyl	PF00234
Serum albumin	ALBUMIN	Serum_albumin	PF00273
Ami_2	Ami_2	Amidase_2	PF01510
Ami_3	Ami_3	Amidase_3	PF01520
Adhesion-associated domain present in MUC4 and other proteins	AMOP	AMOP	PF03782
Anaphylatoxin homologous domain	ANATO	ANATO	PF01821
APPLE domain	APPLE	PAN	PF00024
Bulb-type mannose-specific lectin	B_lectin	B_lectin	PF01453
Bacterial Ig-like domain (group 1)	BID_1	Big_1	PF02369
Bacterial Ig-like domain 2	BID_2	Big_2	PF02368
Bacterial OsmY and nodulation domain	BON	BON	PF04972
Bowman-Birk type proteinase inhibitor	BowB	Bowman-Birk_leg	PF00228
BPI/LBP/CETP N-terminal domain	BPI1	LBP_BPI_CETP	PF01273
Complement component C1q domain	C1Q	C1q	PF00386
Netrin C-terminal Domain	C345C	NTR	PF01759
C-terminal tandem repeated domain in type 4 procollagens	C4	C4	PF01413
Cadherin repeats	CA	Cadherin	PF00028
Calcitonin	CALCITONIN	Calc_CGRP_IAPP	PF00214
Cellulose Binding Domain Type IV	CBD_IV	CBM_6	PF03422
Domain abundant in complement control proteins; SUSHI repeat; short complement-like repeat (SCR)	CCP	Sushi	PF00084
CFEM	CFEM	CFEM	PF05730
A domain in the BMP inhibitor chordin and in microbial proteins	CHRD	CHRD	PF07452
Chitin binding domain	ChtBD1	Chitin_bind_1	PF00187
Chitin-binding domain type 2	ChtBD2	CBM_14	PF01607
Chitin-binding domain type 3	ChtBD3	CBM_5_12	PF02839
Clostridial hydrophobic, with a conserved W residue, domain	ChW	ChW	PF01584
CLUSTERIN alpha chain	CLa	Clusterin	PF01093
CLUSTERIN Beta chain	CLb	Clusterin	PF01093
C-type lectin (CTL) or carbohydrate-recognition domain (CRD)	CLECT	Lectin_C	PF00059
Connexin homologues	CNX	Connexin	PF00029
Fibrillar collagens C-terminal domain	COLFI	COLFI	PF01410
Corticotropin-releasing factor	CRF	CRF	PF00473

*Highlighted **extracellular** domain families found to be multilocale.

**Description**	**SMART name**	**Pfam name**	**Pfam ID**
Granulocyte-macrophage colony-simulating factor (GM-CSF)	CSF2	GM_CSF	PF01109
Domain first found in C1r, C1s, uEGF, and bone morphogenetic protein	CUB	CUB	PF00431
Defensin/corticostatin family	DEFSN	Defensin_beta	PF00711
Homologues of snake disintegrins	DISIN	Disintegrin	PF00200
DM4/DM12 family of domains in Drosophila melanogaster proteins of unknown function	DM4_12	DM4_12	PF07841
Cysteine-rich domain currently specific to Drosophila	DM6	DUF1431	PF07248
Possible catecholamine-binding domain present in a variety of eukaryotic proteins	DoH	DOMON	PF03351
Delta serrate ligand	DSL	DSL	PF01414
Epidermal growth factor-like domain	EGF	EGF	PF00008
Calcium-binding EGF-like domain	EGF_CA	EGF_CA	PF07645
Laminin-type epidermal growth factor-like domain	EGF_Lam	Laminin_EGF	PF00053
Endothelin	END	Endothelin	PF00322
Ependymins	EPEND	Ependymin	PF00811
Ephrin receptor ligand binding domain	EPH_lbd	Ephrin_lbd	PF01404
Four repeated domains in the Fasciclin I family of proteins, present in many other contexts	FAS1	Fasciclin	PF02469
Fibrinogen-related domains (FReDs)	FBG	Fibrinogen_C	PF00147
Fungal-type cellulose-binding domain	fCBD	CBM_1	PF00734
Fibronectin type 1 domain	FN1	fn1	PF00039
Fibronectin type 2 domain	FN2	fn2	PF00040
Frizzled	FRI	Fz	PF01392
Furin-like repeats	FU	Furin-like	PF00757
G2 nidogen domain and fibulin	G2F	G2F	PF07474
Gastrin / cholecystokinin / caerulein family	GASTRIN	Gastrin	PF00918
Glycoprotein hormone alpha chain homologues	GHA	Hormone_6	PF00236
Glycoprotein hormone beta chain homologues	GHB	Cys_knot	PF00007
Domain containing Gla (gamma-carboxyglutamate) residues	GLA	Gla	PF00594
Glucagon like hormones	GLUCA	Hormone_2	PF00123
Glycosyl hydrolase family 10	Glyco_10	Glyco_hydro_10	PF00331
Glyco_18	Glyco_18	Glyco_hydro_18	PF00704
Glycosyl hydrolases family 25	Glyco_25	Glyco_hydro_25	PF01183
Glycosyl hydrolases family 32	Glyco_32	Glyco_hydro_32	PF00251
G-protein-coupled receptor proteolytic site domain	GPS	GPS	PF01825
Granulin	GRAN	Granulin	PF00396
Hint (Hedgehog/Intein) domain C-terminal region	HintC	Hint	PF01079
Hint (Hedgehog/Intein) domain N-terminal region	HintN	Hint	PF01079

*Highlighted **extracellular** domain families found to be multilocale.

Description	SMART name	Pfam name	Pfam ID
Domain present in hormone receptors	HormR	HRM	PF02793
Hemopexin-like repeats	HX	Hemopexin	PF00045
Hydrophobins	HYDRO	Hydrophobin	PF01185
Insulin growth factor-binding protein homologues	IB	IGFBP	PF00219
Interferon alpha, beta and delta	IFabd	Interferon	PF00143
Interleukin-10 family	IL10	IL10	PF00726
Interleukin-2 family	IL2	IL2	PF00715
Interleukins 4 and 13	IL4_13	IL4	PF00727
Interleukin-6 homologues	IL6	IL6	PF00489
Interleukin-7 and interleukin-9 family	IL7	IL7	PF01415
Insulin / insulin-like growth factor / relaxin family	IlGF	Insulin	PF00049
Integrin beta subunits (N-terminal portion of extracellular region)	INB	Integrin_beta	PF00362
Integrin alpha (beta-propellor repeats)	Int_alpha	Integrin_alpha	PF00357
Kazal type serine protease inhibitors	KAZAL	Kazal_1	PF00050
Kazal type serine protease inhibitors	KAZAL	Kazal_2	PF07648
Knottins	Knot1	Defensin_2	PF01097
Kringle domain	KR	Kringle	PF00051
BPTI/Kunitz family of serine protease inhibitors	KU	Kunitz_BPTI	PF00014
Laminin B domain	LamB	Laminin_B	PF00052
Laminin G domain	LamG	Laminin_G_1	PF00054
Laminin N-terminal domain (domain VI)	LamNT	Laminin_N	PF00055
LCCL	LCCL	LCCL	PF03815
Low-density lipoprotein receptor domain class A	LDLa	Ldl_recept_a	PF00057
Leukemia inhibitory factor	LIF_OSM	LIF_OSM	PF01291
Link (Hyaluronan-binding)	LINK	Xlink	PF00193
Lipoprotein N-terminal Domain	LPD_N	Vitellogenin_N	PF01347
Ly-6 antigen / uPA receptor -like domain	LU	UPAR_LY6	PF00021
Low-density lipoprotein-receptor YWTD domain	LY	Ldl_recept_b	PF00058
Alpha-lactalbumin / lysozyme C	LYZ1	Lys	PF00062
Lysozyme subfamily 2	LYZ2	Glucosaminidase	PF01832
Membrane-attack complex / perforin	MACPF	MACPF	PF01823
Domain in meprin, A5, receptor protein tyrosine phosphatase mu (and others)	MAM	MAM	PF00629
Domain involved in innate immunity and lipid metabolism	ML	E1_DerP2_DerF2	PF02221
NEAr Transporter domain	NEAT	NEAT	PF05031
Nerve growth factor (NGF or beta-NGF)	NGF	NGF	PF00243
Neurohypophysial hormones	NH	Hormone_5	PF00184

*Highlighted **extracellular** domain families found to be multilocale.

Description	SMART name	Pfam name	Pfam ID
Extracellular domain of unknown function in nidogen (entactin) and hypothetical proteins	NIDO	NIDO	PF06119
Domain found in Notch and Lin-12	NL	Notch	PF00066
Neuromedin U	NMU	NMU	PF02070
Tissue inhibitor of metalloproteinase family	NTR	TIMP	PF00965
DNA/RNA non-specific endonuclease*	NUC	Endonuclease_NS	PF01223
Olfactomedin-like domains	OLF	OLF	PF02191
Osteopontin	OSTEO	Osteopontin	PF00865
Phospholipase A2	PA2c	Phospholip_A2_1	PF00068
Pancreatic hormones / neuropeptide F / peptide YY family	PAH	Hormone_3	PF00159
Divergent subfamily of APPLE domains	PAN_AP	PAN	PF00024
Bacterial periplasmic substrate-binding proteins	PBPb	SBP_bac_3	PF00497
P or trefoil or TFF domain	PD	Trefoil	PF00088
Platelet-derived and vascular endothelial growth factors (PDGF, VEGF) family	PDGF	PDGF	PF00341
Papain family cysteine protease	Pept_C1	Peptidase_C1	PF00112
Insect pheromone/odorant binding protein domains	PhBP	PBP_GOBP	PF01395
Repeats in polycystic kidney disease 1 (PKD1) and other proteins	PKD	PKD	PF00801
Major prion protein	PRP	Prion	PF00377
Paramecium Surface Antigen Repeat	PSA	Paramecium_SA	PF01508
Domain found in Plexins, Semaphorins and Integrins	PSI	PSI	PF01437
Plant trypsin inhibitors	PTI	Squash	PF00299
Pentraxin / C-reactive protein / pentaxin family	PTX	Pentaxin	PF00354
Pancreatic ribonuclease	RNAse_Pc	RnaseA	PF00074
Serum amyloid A proteins	SAA	SAA	PF00277
Saposin/surfactant protein-B A-type DOMAIN	SAPA	SapA	PF02199
Saposin (B) Domains	SapB	SapB_1	PF05184
Saposin (B) Domains	SapB	SapB_2	PF03489
SCP / Tpx-1 / Ag5 / PR-1 / Sc7 family of extracellular domains	SCP	SCP	PF00188
Intercrine alpha family (small cytokine C-X-C) (chemokine CXC)	SCY	IL8	PF00048
Domain found in sea urchin sperm protein, enterokinase, agrin	SEA	SEA	PF01390
Semaphorin domain	Sema	Sema	PF01403
ShK toxin domain	ShKT	ShTK	PF01549
Somatomedin B -like domains	SO	Somatomedin_B	PF01033
Scavenger receptor Cys-rich	SR	SRCR	PF00530

*Highlighted **extracellular** domain families found to be multilocale.

Description	SMART name	Pfam name	Pfam ID
Soybean trypsin inhibitor (Kunitz) family of protease inhibitors	STI	Kunitz_legume	PF00197
Transforming growth factor-beta (TGF-beta) family	TGFB	TGF_beta	PF00019
Thaumatin family	THN	Thaumatin	PF00314
Tachykinin family	TK	Tachykinin	PF02202
Tumour necrosis factor family	TNF	TNF	PF00229
Tumor necrosis factor receptor / nerve growth factor receptor repeats	TNFR	TNFR_c6	PF00020
Transferrin	TR_FER	Transferrin	PF00405
Transthyretin	TR_THY	Transthyretin	PF00576
Trypsin-like serine protease	Tryp_SPc	Trypsin	PF00089
Thrombospondin type 1 repeats	TSP1	TSP_1	PF00090
Thyroglobulin type I repeats	TY	Thyroglobulin_1	PF00086
Uteroglobin	UTG	Uteroglobin	PF01099
von Willebrand factor (vWF) type C domain	VWC	VWC	PF00093
von Willebrand factor (vWF) type D domain	VWD	VWD	PF00094
Four-disulfide core domains	WAP	WAP	PF00095
Wnt-inhibitory factor-1 like domain	WIF	WIF	PF02019
Found in Wnt-1	WNT1	wnt	PF00110
Present in yeast cell wall integrity and stress response component proteins	WSC	WSC	PF01822
Zinc-dependent metalloprotease	ZnMc	Peptidase_M10	PF00413
Zona pellucida (ZP) domain	ZP	Zona_pellucida	PF00100
ADAM-TS Spacer 1	-	ADAM_spacer1	PF05986
Animal haem peroxidase	-	An_peroxidase	PF03098
Antistasin	-	Antistasin	PF02822
Copper type II ascorbate-dependent monooxygenase, C-terminal domain	-	Cu2_monoox_C	PF03712
Copper type II ascorbate-dependent monooxygenase, N-terminal domain	-	Cu2_monooxygen	PF01082
Laminin Domain I	-	Laminin_I	PF06008
Laminin Domain II	-	Laminin_II	PF06009
Laminin G domain	TSPN	Laminin_G_2	PF02210
Class I Histocompatibility antigen, domains alpha 1 and 2	-	MHC_I	PF00129
Class II histocompatibility antigen, beta domain	-	MHC_II_beta	PF00969
Agrin NtA domain	-	NtA	PF03146
Reprolysin (M12B) family zinc metalloprotease	-	Reprolysin	PF01421

*Highlighted **extracellular** domain families found to be multilocale.
