# Supplementary material for: Identification and correction of abnormal, incomplete and mispredicted proteins in public databases
Source: BMC Bioinformatics. 2008 Aug 27;9:353. doi: 10.1186/1471-2105-9-353 (PMC2542381; doi:10.1186/1471-2105-9-353)
Supplement: Additional file 4 — List of intracellular Pfam-A signaling domain families. The file contains the list of intracellular Pfam-A signaling domain families. [file 1471-2105-9-353-S4.pdf]

Additional file 4. List of intracellular Pfam-A signaling domain families. The table contains obligatory cytoplasmic Pfam-A domain families which were used to predict subcellular localization of proteins. Our domain co-occurrence analyses of Metazoan UniProtKB entries have identified 115 obligatory cytoplasmic Pfam-A domain families, the majority of which are also identified as such in the SMART database.

Description	SMART name	Pfam name	Pfam ID
14-3-3 homologues	14_3_3	14-3-3	PF00244
Actin depolymerisation factor/cofilin -like domains	ADF	Cofilin_ADF	PF00241
Ankyrin repeats	ANK	Ank	PF00023
ARF-like small GTPases; ARF, ADP-ribosylation factor	ARF	Arf	PF00025
Putative GTP-ase activating proteins for the small GTPase, ARF	ArfGap	ArfGap	PF01412
Band 4.1 homologues	B41	Band_41	PF00373
Cytochrome b-561 / ferric reductase transmembrane domain	B561	Cytochrom_B561	PF03188
Bulb-type mannose-specific lectin	B_lectin	B_lectin	PF01453
BAG domains, present in regulator of Hsp70 proteins	BAG	BAG	PF02179
BH4 Bcl-2 homology region 4	BH4	BH4	PF02180
Bruton's tyrosine kinase Cys-rich motif	BTK	BTK	PF00779
Protein kinase C conserved region 1 (C1) domains (Cysteine-rich domains)	DAG_PE-bind	C1_1	PF00130
Calponin homology domain	CH	CH	PF00307
Two component signalling adaptor domain	CheW	CheW	PF01584
Domain found in NIK1-like kinases, mouse citron and yeast ROM1, ROM2	CNH	CNH	PF00780
Cyclic nucleotide-monophosphate binding domain	cNMP	cNMP_binding	PF00027
Cullin	CULLIN	Cullin	PF00888
Adenylyl- / guanylyl cyclase, catalytic domain	CYCc	Guanylate_cyc	PF00211
Diacylglycerol kinase accessory domain (presumed)	DAGKa	DAGK_acc	PF00609
Diacylglycerol kinase catalytic domain (presumed)	DAGKc	DAGK_cat	PF00781
Domain present in Dishevelled and axin	DAX	DIX	PF00778
Death effector domain	DED	DED	PF01335
Domain found in Dishevelled, Egl-10, and Pleckstrin	DEP	DEP	PF00610
Dual specificity phosphatase, catalytic domain	DSPc	DSPc	PF00782
Domain of Unknown Function with GGDEF motif	DUF1	GGDEF	PF00990
Domain of Unknown Function 2	DUF2	EAL	PF00563
Dynamin, GTPase	DYNc	Dynamin_N	PF00350
Epsin N-terminal homology (ENTH) domain	ENTH	ENTH	PF01417
A Receptor for Ubiquitination Targets	FBOX	F-box	PF00646
Fes/CIP4 homology domain	FCH	FCH	PF00611
Contains two conserved F residues	FF	FF	PF01846
Formin Homology 2 Domain	FH2	FH2	PF02181
Forkhead associated domain	FHA	FHA	PF00498
Protein present in Fab1, YOTB, Vac1, and EEA1	FYVE	FYVE	PF01363
Growth-Arrest-Specific Protein 2 Domain	GAS2	GAS2	PF02187
Dynamin GTPase effector domain	GED	GED	PF02212

Description	SMART name	Pfam name	Pfam ID
G protein gamma subunit-like motifs	GGL	G-gamma	PF00631
LGN motif, putative GEFs specific for G-alpha GTPases	GoLoco	GoLoco	PF02188
Domain in glucosyltransferases, myotubularins and other putative membrane-associated proteins	GRAM	GRAM	PF02893
Guanylate kinase homologues	GuKc	Guanylate_kin	PF00625
HAMP (Histidine kinases, Adenylyl cyclases, Methyl binding proteins, Phosphatases) domain	HAMP	HAMP	PF00672
Histidine kinase-like ATPases	HATPase_c	HATPase_c	PF02518
Domain Homologous to E6-AP Carboxyl Terminus with	HECTc	HECT	PF00632
His Kinase A (phosphoacceptor) domain	HisKA	HisKA	PF00512
Histidine Phosphotransfer domain	HPT	Hpt	PF01627
Rho effector or protein kinase C-related kinase homology region 1 homologues	Hr1	HR1	PF02185
I/LWEQ domain	ILWEQ	I_LWEQ	PF01608
Inositol polyphosphate phosphatase, catalytic domain homologues	IPPc	Exo_endo_phos	PF03372
Short calmodulin-binding motif containing conserved Ile and Gln residues	IQ	IQ	PF00612
Immunoreceptor tyrosine-based activation motif	ITAM	ITAM	PF02189
Kinesin motor, catalytic domain. ATPase	KISc	Kinesin	PF00225
Domain in receptor targeting proteins Lin-2 and Lin-7	L27	L27	PF02828
Zinc-binding domain present in Lin-11, Isl-1, Mec-3	LIM	LIM	PF00412
Low molecular weight phosphatase family	LMWPc	LMWPc	PF01451
Methyl-accepting chemotaxis-like domains (chemotaxis sensory transducer)	MA	MCPsignal	PF00015
Myosin	MYSc	Myosin_head	PF00063
Domain in Myosin and Kinesin Tails	MyTH4	MyTH4	PF00784
Motif C-terminal to PAS motifs (likely to contribute to PAS structural domain)	PAC	PAC	PF00785
PAS domain	PAS	PAS	PF00989
PASTA	PASTA	PASTA	PF03793
P21-Rho-binding domain	PBD	PBD	PF00786
Pleckstrin homology domain	PH	PH	PF00169
Phosphoinositide 3-kinase, region postulated to contain C2 domain	PI3K_C2	PI3K_C2	PF00792
PI3-kinase family, p85-binding domain	PI3K_p85B	PI3K_p85B	PF02192
PI3-kinase family, Ras-binding domain	PI3K_rbd	PI3K_rbd	PF00794
Phosphoinositide 3-kinase family, accessory domain (PIK domain)	PI3Ka	PI3Ka	PF00613
Phosphoinositide 3-kinase, catalytic domain	PI3Kc	PI3_PI4_kinase	PF00454
Phosphatidylinositol phosphate kinases	PIPKc	PIP5K	PF01504
Cytoplasmic phospholipase A2, catalytic subunit	PLAc	PLA2_B	PF01735

**Description**	**SMART name**	**Pfam name**	**Pfam ID**
Phospholipase C, catalytic domain (part); domain X	PLCXc	PI-PLC-X	PF00388
Phospholipase C, catalytic domain (part); domain Y	PLCYc	PI-PLC-Y	PF00387
Phospholipase D. Active site motifs	PLDc	PLDc	PF00614
Serine/threonine phosphatases, family 2C, catalytic domain	PP2Cc	PP2C	PF00481
Phosphotyrosine-binding domain, phosphotyrosine-interaction (PI) domain	PTB	PID	PF00640
Phosphotyrosine-binding domain (IRS1-like)	PTBI	IRS	PF02174
Protein tyrosine phosphatase, catalytic domain	PTPc	Y_phosphatase	PF00102
Protein tyrosine phosphatase, catalytic domain, undefined specificity	PTPc_DSPc	Y_phosphatase	PF00102
Protein tyrosine phosphatase, catalytic domain motif	PTPc_motif	Y_phosphatase	PF00102
PhoX homologous domain, present in p47phox and p40phox	PX	PX	PF00787
Domain associated with PX domains	PXA	PXA	PF02194
Ras association (RalGDS/AF-6) domain	RA	RA	PF00788
Ran-binding domain	RanBD	Ran_BP1	PF00638
Ras subfamily of RAS small GTPases	RAS	Ras	PF00071
GTPase-activator protein for Ras-like GTPases	RasGAP	RasGAP	PF00616
Guanine nucleotide exchange factor for Ras-like small GTPases	RasGEF	RasGEF	PF00617
Guanine nucleotide exchange factor for Ras-like GTPases; N-terminal motif	RasGEFN	RasGEF_N	PF00618
Raf-like Ras-binding domain	RBD	RBD	PF02196
Regulator of G protein signalling domain	RGS	RGS	PF00615
GTPase-activator protein for Rho-like GTPases	RhoGAP	RhoGAP	PF00620
Guanine nucleotide exchange factor for Rho/Rac/Cdc42-like GTPases	RhoGEF	RhoGEF	PF00621
RIIalpha, Regulatory subunit portion of type II PKA R-subunit	RIIa	RIIa	PF02197
RIO-like kinase	RIO	RIO1	PF01163
Extension to Ser/Thr-type protein kinases	S_TK_X	Pkinase_C	PF00433
Serine/Threonine protein kinases, catalytic domain	S_TKc	Pkinase	PF00069
Sterile alpha motif	SAM	SAM_1	PF00536
Sterile alpha motif	SAM	SAM_2	PF07647
Sec7 domain	Sec7	Sec7	PF01369
Src homology 2 domains	SH2	SH2	PF00017
Suppressors of cytokine signalling	SOCS	SOCS_box	PF07525
Spectrin repeats	SPEC	Spectrin	PF00435
In StAR and phosphatidylcholine transfer protein	START	START	PF01852
Protein kinase; unclassified specificity	STYKc	Pkinase	PF00069
Helical region found in SNAREs	t_SNARE	SNARE	PF05739

Description	SMART name	Pfam name	Pfam ID
Homologues of the ligand binding domain of Tar	TarH	TarH	PF02203
Domain in Tre-2, BUB2p, and Cdc16p. Probable Rab-GAPs	TBC	TBC	PF00566
Metallochaperone-like domain	TRASH	YHS	PF04945
Tyrosine kinase, catalytic domain	TyrKc	Pkinase	PF00069
Ubiquitin associated domain	UBA	UBA	PF00627
Ubiquitin homologues	UBQ	ubiquitin	PF00240
Domain present in ubiquitin-regulatory proteins	UBX	UBX	PF00789
Domain present in VPS-27, Hrs and STAM	VHS	VHS	PF00790
Domain present in VPS9	VPS9	VPS9	PF02204
WD40 repeats	WD40	WD40	PF00400
WASP homology region 1	WH1	WH1	PF00568
Wiskott Aldrich syndrome homology region 2	WH2	WH2	PF02205
Domain with 2 conserved Trp (W) residues	WW	WW	PF00397
A20-like zinc fingers	ZnF_A20	zf-A20	PF01754
Zinc-binding domain, present in Dystrophin, CREB-binding protein	ZnF_ZZ	ZZ	PF00569
Domain present in ZO-1 and Unc5-like netrin receptors	ZU5	ZU5	PF00791
