# Supplementary material for: Identification and correction of abnormal, incomplete and mispredicted proteins in public databases
Source: BMC Bioinformatics. 2008 Aug 27;9:353. doi: 10.1186/1471-2105-9-353 (PMC2542381; doi:10.1186/1471-2105-9-353)
Supplement: Additional file 5 — List of nuclear Pfam-A domain families. The file contains the list of nuclear Pfam-A domain families. [file 1471-2105-9-353-S5.pdf]

Additional file 5. List of nuclear Pfam-A domain families. The table contains obligatory nuclear Pfam-A domain families which were used to predict subcellular localization of proteins. Our domain co-occurrence analyses of Metazoan UniProtKB entries have identified 126 obligatory nuclear Pfam-A domain families, the majority of which are also identified as such in the SMART database.

**Description**	**SMART name**	**Pfam name**	**Pfam ID**
3'-5' exonuclease	35EXOc	3_5_exonuc	PF01612
5'-3' exonuclease	53EXOc	5_3_exonuc	PF01367
tRNA-specific and double-stranded RNA adenosine deaminase (RNA-specific editase)	ADEAMc	A_deamin	PF02137
DNA-binding domain in plant proteins such as APETALA2 and EREBPs	AP2	AP2	PF00847
AP endonuclease family 2	AP2Ec	AP_endonuc_2	PF01261
Bromo adjacent homology domain	BAH	BAH	PF01426
Basic domain in HLH proteins of MYOD family	BASIC	Basic	PF01586
B-Box-type zinc finger	BBOX	zf-B_box	PF00643
BCL (B-Cell lymphoma); contains BH1, BH2 regions	BCL	Bcl-2	PF00452
Breast cancer carboxy-terminal domain	BRCT	BRCT	PF00533
BRIGHT, ARID (A/T-rich interaction domain) domain	BRIGHT	ARID	PF01388
Domain in transcription and CHROMO domain helicases	BRK	TCH	PF07533
Basic region leucin zipper	BRLZ	bZIP	PF00170
Bromo domain	BROMO	Bromodomain	PF00439
Domain in transcription factors and synapse-associated proteins	BSD	BSD	PF03909
Bromodomain transcription factors and PHD domain containing proteins	BTP	Bromo_TP	PF07524
CCAAT-Binding transcription Factor	CBF	CBFB_NFYA	PF02045
Chromatin organization modifier domain	CHROMO	Chromo	PF00385
Catalytic domain of ctd-like phosphatases	CPDc	NIF	PF03031
Cold shock protein domain	CSP	CSD	PF00313
Copper-Fist	Cu_Fist	Copper-fist	PF00649
Domain in different transcription and chromosome remodeling factors	DDT	DDT	PF02791
DEAD-like helicases superfamily	DEXDc	SNF2_N	PF00176
Doublesex DNA-binding motif	DM	DM	PF00751
Double-stranded RNA binding motif	DSRM	dsrm	PF00035
Domain A in dwarfin family proteins	DWA	MH1	PF03165
Domain B in dwarfin family proteins	DWB	MH2	PF03166
Domain in DSRM or ZnF_C2H2 domain containing proteins	DZF	DZF	PF07528
Domain at the C-termini of GCD6, eIF-2B epsilon, eIF-4 gamma and eIF-5	eIF5C	W2	PF02020
Elongator protein 3, MiaB family, Radical SAM	Elp3	Radical_SAM	PF04055
Endonuclease III	ENDO3c	HhH-GPD	PF00730
Erythroblast transformation specific domain	ETS	Ets	PF00178

Description	SMART name	Pfam name	Pfam ID
EXOIII	EXOIII	Exonuc_X-T	PF00929
FORKHEAD	FH	Fork_head	PF00250
"FY-rich" domain, C-terminal region	FYRC	FYRC	PF05965
"FY-rich" domain, N-terminal region	FYRN	FYRN	PF05964
Glycine rich nucleic binding domain	G_patch	G-patch	PF01585
GIY-YIG type nucleases (URI domain)	GIYc	GIY-YIG	PF01541
Domain in histone families 1 and 5	H15	Linker_histone	PF00538
Histone 2A	H2A	Histone	PF00125
Histone H2B	H2B	Histone	PF00125
Histone H3	H3	Histone	PF00125
Histone H4	H4	Histone	PF00125
Homeobox associated leucin zipper	HALZ	HALZ	PF02183
Helicase superfamily c-terminal domain	HELICc	Helicase_C	PF00271
Helix-hairpin-helix DNA-binding motif class 1	HhH1	HHH	PF00633
High mobility group	HMG	HMG_box	PF00505
Domain in high mobilty group proteins HMG14 and HMG 17	HMG17	HMG14_17	PF01101
HNH nucleases	HNHc	HNH	PF01844
Domain in histone-like proteins of HNS family	HNS	Histone_HNS	PF00816
Ligand binding domain of hormone receptors	HOLI	Hormone_recep	PF00104
Helicase and RNase D C-terminal	HRDC	HRDC	PF00570
Domain in helicases and associated with SANT domains	HSA	HSA	PF07529
Helix_turn_helix, arabinose operon control protein	HTH_ARAC	HTH_AraC	PF00165
Helix_turn_helix, Arsenical Resistance Operon Repressor	HTH_ARSR	HTH_5	PF01022
Helix_turn_helix ASNC type	HTH_ASNC	AsnC_trans_reg	PF01037
Helix_turn_helix, Deoxyribose operon repressor	HTH_DEOR	DeoR	PF00455
Helix-turn-helix diphteria tox regulatory element	HTH_DTXR	Fe_dep_repress	PF01325
Helix_turn_helix gluconate operon transcriptional repressor	HTH_GNTR	GntR	PF00392
Helix_turn _helix lactose operon repressor	HTH_LACI	LacI	PF00356
Helix_turn_helix multiple antibiotic resistance protein	HTH_MARR	MarR	PF01047
Helix_turn_helix, mercury resistance	HTH_MERR	MerR	PF00376
Helix-turn-helix XRE-family like proteins	HTH_XRE	HTH_3	PF01381
Intron encoded nuclease repeat motif	IENR1	NUMOD1	PF07453
Intron-encoded nuclease repeat 2	IENR2	NUMOD3	PF07460
Interferon regulatory factor	IRF	IRF	PF00605
A domain family that is part of the cupin metalloenzyme superfamily	JmjC	JmjC	PF02373

Description	SMART name	Pfam name	Pfam ID
Small domain found in the jumonji family of transcription factors	JmjN	JmjN	PF02375
KOW (Kyprides, Ouzounis, Woese) motif	KOW	KOW	PF00467
In nuclear membrane-associated proteins	LEM	LEM	PF03020
Ligase N family	LIGANc	DNA_ligase_aden	PF01653
Domain in DAP-5, eIF4G, MA-3 and other proteins	MA3	MA3	PF02847
MADS	MADS	SRF-TF	PF00319
Methyl-CpG binding domain	MBD	MBD	PF01429
Minichromosome maintenance proteins	MCM	MCM	PF00493
ATPase domain of DNA mismatch repair MUTS family	MUTSac	MutS_V	PF00488
NEUZ	NEUZ	Neuralized	PF07177
Orange domain	ORANGE	Hairy_orange	PF07527
ParB-like nuclease domain	ParB	ParBc	PF02195
Paired Box domain	PAX	PAX	PF00292
PHD zinc finger	PHD	PHD	PF00628
Short conserved domain in transcriptional regulators	Plus3	Plus-3	PF03126
DNA polymerase III beta subunit	POL3Bc	DNA_pol3_beta	PF00712
DNA polymerase A domain	POLAc	DNA_pol_A	PF00476
DNA polymerase type-B family	POLBc	DNA_pol_B	PF00136
DNA polymerase alpha chain like domain	POLIIIAc	PHP	PF02811
A domain found in a protein subunit of human RNase MRP and RNase P ribonucleoprotein complexes and archaeal proteins	POP4	UPF0086	PF01868
Found in Pit-Oct-Unc transcription factors	POU	Pou	PF00157
Domain associated with HOX domains	POX	POX	PF07526
PRE_C2HC	PRE_C2HC	PRE_C2HC	PF07530
N-terminal to some SET domains	PreSET	Pre-SET	PF05033
Proline-rich domain in spliceosome associated proteins	PSP	PSP	PF04046
DNA/RNA-binding repeats in PUR-alpha/beta/gamma and in hypothetical proteins from spirochetes and the Bacteroides-Cytophaga-Flexibacter bacteria	PUR	PurA	PF04845
Ribonuclease III family	RIBOc	Ribonuclease_3	PF00636
DNA-directed RNA-polymerase II subunit	RPOL4c	RNA_pol_Rpb4	PF03874
RNA polymerase subunit 8	RPOL8c	RNA_pol_Rpb8	PF03870
RNA polymerase subunit 9	RPOL9	RNA_POL_M_15KD	PF02150
RNA polymerase I subunit A N-terminus	RPOLA_N	RNA_pol_Rpb1_2	PF00623
RNA polymerase subunit CX	RPOLCX	DNA_RNApol_7kD	PF03604
RNA polymerases D	RPOLD	RNA_pol_A_bac	PF01000

Description	SMART name	Pfam name	Pfam ID
RPR	RPR	DUF618	PF04818
S4 RNA-binding domain	S4	S4	PF01479
Putative DNA-binding (bihelical) motif predicted to be involved in chromosomal organisation	SAP	SAP	PF02037
SET (Su(var)3-9, Enhancer-of-zeste, Trithorax) domain	SET	SET	PF00856
Found in Skp1 protein family	Skp1	Skp1	PF01466
snRNP Sm proteins	Sm	LSM	PF01423
SPK	SPK	SPK	PF04435
SET and RING finger associated domain	SRA	YDG_SRA	PF02182
STE like transcription factors	STE	STE	PF02200
Domain first found in the mice T locus (Brachyury) protein	TBOX	T-box	PF00907
TEA domain	TEA	TEA	PF01285
Transcription initiation factor IIE	TFIIE	TFIIE_alpha	PF02002
Domain in the central regions of transcription elongation factor S-II (and elsewhere)	TFS2M	TFIIS_M	PF07500
Bacterial DNA topoisomerase I DNA-binding domain	TOP1Ac	Topoisom_bac	PF01131
Bacterial DNA topoisomeraes I ATP-binding domain	TOP1Bc	Topoisom_bac	PF01131
DNA Topoisomerase I (eukaryota)	TOPEUc	Topoisom_I	PF01028
TOPRIM	TOPRIM	Toprim	PF01751
Xeroderma pigmentosum G I-region	XPGI	XPG_I	PF00867
Xeroderma pigmentosum G N-region	XPGN	XPG_N	PF00752
Z-DNA-binding domain in adenosine deaminases	Zalpha	z-alpha	PF02295
BED zinc finger	ZnF_BED	zf-BED	PF02892
C2C2 Zinc finger	ZnF_C2C2	TFIIS_C	PF01096
Zinc finger	ZnF_C2H2	zf-C2H2	PF00096
Zinc finger	ZnF_C2HC	zf-CCHC	PF00098
Zinc finger	ZnF_C3H1	zf-CCCH	PF00642
C4 zinc finger in nuclear hormone receptors	ZnF_C4	zf-C4	PF00105
Zinc finger	ZnF_CHCC	zf-CHC2	PF01807
Zinc finger in DBF-like proteins	ZnF_DBF	zf-DBF	PF07535
Zinc finger binding to DNA consensus sequence [AT]GATA[AG]	ZnF_GATA	GATA	PF00320
Plant mutator transposase zinc finger	ZnF_PMZ	SWIM	PF04434
