# Supplementary material for: Identification and correction of abnormal, incomplete and mispredicted proteins in public databases
Source: BMC Bioinformatics. 2008 Aug 27;9:353. doi: 10.1186/1471-2105-9-353 (PMC2542381; doi:10.1186/1471-2105-9-353)
Supplement: Additional file 6 — List of Pfam-A domain families suitable for the study of domain integrity. The file contains the list of Pfam-A domain families suitable for the study of domain integrity. [file 1471-2105-9-353-S6.pdf]

Additional file 6. List of Pfam-A domain families suitable for the study of domain integrity. The table contains Pfam-A domain families present in human, vertebrate and metazoa+fungi Swiss-Prot proteins that have a well-defined, conserved sequence length range and thus proved to be suitable for the study of domain integrity. The detailed description of the identification of Pfam-A families suitable for the study of the domain integrity is found in Additional file 1, Part 2.

**Human** Pfam ID												
PF00001	PF00323	PF00694	PF01119	PF01549	PF02171	PF02823	PF03487	PF04263	PF05060	PF05997	PF07169	PF08202
PF00005	PF00324	PF00696	PF01120	PF01551	PF02172	PF02824	PF03489	PF04265	PF05063	PF06001	PF07175	PF08204
PF00006	PF00326	PF00698	PF01121	PF01553	PF02173	PF02825	PF03491	PF04272	PF05064	PF06008	PF07177	PF08210
PF00009	PF00327	PF00704	PF01122	PF01556	PF02174	PF02826	PF03493	PF04275	PF05071	PF06009	PF07189	PF08213
PF00010	PF00328	PF00705	PF01124	PF01557	PF02177	PF02827	PF03494	PF04280	PF05076	PF06010	PF07202	PF08215
PF00013	PF00329	PF00708	PF01125	PF01562	PF02178	PF02828	PF03501	PF04300	PF05083	PF06012	PF07221	PF08216
PF00014	PF00330	PF00709	PF01126	PF01564	PF02179	PF02836	PF03508	PF04376	PF05089	PF06017	PF07225	PF08221
PF00017	PF00333	PF00711	PF01127	PF01565	PF02180	PF02837	PF03509	PF04377	PF05090	PF06025	PF07260	PF08232
PF00019	PF00334	PF00714	PF01129	PF01566	PF02181	PF02838	PF03511	PF04379	PF05091	PF06026	PF07263	PF08235
PF00021	PF00335	PF00715	PF01130	PF01569	PF02182	PF02840	PF03516	PF04382	PF05093	PF06031	PF07264	PF08240
PF00024	PF00337	PF00717	PF01131	PF01571	PF02184	PF02841	PF03517	PF04388	PF05104	PF06046	PF07281	PF08241
PF00025	PF00338	PF00719	PF01133	PF01575	PF02185	PF02843	PF03520	PF04389	PF05110	PF06049	PF07286	PF08242
PF00026	PF00339	PF00727	PF01134	PF01582	PF02186	PF02844	PF03521	PF04406	PF05111	PF06052	PF07292	PF08246
PF00027	PF00341	PF00728	PF01135	PF01583	PF02187	PF02845	PF03522	PF04408	PF05118	PF06058	PF07297	PF08264
PF00029	PF00342	PF00731	PF01138	PF01585	PF02189	PF02847	PF03523	PF04410	PF05127	PF06060	PF07303	PF08265
PF00030	PF00343	PF00732	PF01140	PF01586	PF02190	PF02852	PF03528	PF04419	PF05131	PF06068	PF07324	PF08266
PF00032	PF00344	PF00733	PF01142	PF01588	PF02191	PF02854	PF03529	PF04420	PF05132	PF06071	PF07334	PF08271
PF00033	PF00346	PF00735	PF01144	PF01590	PF02192	PF02862	PF03530	PF04421	PF05147	PF06079	PF07347	PF08279
PF00034	PF00347	PF00736	PF01146	PF01591	PF02194	PF02864	PF03531	PF04423	PF05148	PF06083	PF07353	PF08285
PF00037	PF00348	PF00743	PF01148	PF01592	PF02196	PF02865	PF03533	PF04427	PF05153	PF06087	PF07354	PF08288
PF00038	PF00349	PF00748	PF01150	PF01596	PF02197	PF02866	PF03535	PF04433	PF05158	PF06105	PF07359	PF08292
PF00039	PF00350	PF00749	PF01151	PF01597	PF02198	PF02867	PF03540	PF04434	PF05160	PF06119	PF07400	PF08295
PF00040	PF00351	PF00750	PF01153	PF01598	PF02199	PF02870	PF03542	PF04437	PF05162	PF06121	PF07406	PF08311
PF00042	PF00352	PF00751	PF01154	PF01599	PF02201	PF02872	PF03546	PF04438	PF05181	PF06140	PF07412	PF08313

PF00044	PF00354	PF00752	PF01157	PF01602	PF02202	PF02874	PF03547	PF04440	PF05184	PF06148	PF07421	PF08318
PF00045	PF00355	PF00753	PF01158	PF01603	PF02204	PF02875	PF03556	PF04442	PF05185	PF06179	PF07443	PF08321
PF00046	PF00357	PF00755	PF01159	PF01608	PF02205	PF02877	PF03561	PF04487	PF05186	PF06189	PF07448	PF08324
PF00048	PF00361	PF00756	PF01160	PF01619	PF02207	PF02879	PF03567	PF04488	PF05187	PF06201	PF07452	PF08326
PF00049	PF00362	PF00757	PF01161	PF01624	PF02208	PF02880	PF03568	PF04494	PF05188	PF06202	PF07458	PF08327
PF00050	PF00363	PF00758	PF01162	PF01625	PF02209	PF02881	PF03571	PF04495	PF05189	PF06209	PF07469	PF08332
PF00051	PF00364	PF00762	PF01163	PF01630	PF02212	PF02882	PF03572	PF04503	PF05190	PF06211	PF07474	PF08333
PF00052	PF00365	PF00763	PF01166	PF01633	PF02213	PF02883	PF03577	PF04505	PF05191	PF06212	PF07479	PF08336
PF00054	PF00366	PF00764	PF01167	PF01642	PF02214	PF02885	PF03587	PF04511	PF05193	PF06214	PF07496	PF08337
PF00055	PF00368	PF00766	PF01168	PF01652	PF02218	PF02887	PF03600	PF04515	PF05195	PF06218	PF07500	PF08344
PF00056	PF00370	PF00769	PF01170	PF01655	PF02219	PF02888	PF03604	PF04538	PF05196	PF06220	PF07502	PF08351
PF00058	PF00375	PF00773	PF01172	PF01661	PF02221	PF02889	PF03607	PF04547	PF05199	PF06221	PF07521	PF08357
PF00060	PF00377	PF00774	PF01175	PF01663	PF02223	PF02891	PF03615	PF04548	PF05207	PF06229	PF07522	PF08365
PF00062	PF00378	PF00777	PF01176	PF01667	PF02225	PF02892	PF03619	PF04549	PF05208	PF06237	PF07524	PF08368
PF00066	PF00380	PF00778	PF01179	PF01669	PF02229	PF02893	PF03623	PF04553	PF05222	PF06248	PF07525	PF08373
PF00068	PF00382	PF00779	PF01180	PF01687	PF02230	PF02897	PF03630	PF04557	PF05236	PF06268	PF07527	PF08374
PF00072	PF00383	PF00780	PF01182	PF01694	PF02233	PF02898	PF03635	PF04558	PF05237	PF06292	PF07528	PF08375
PF00074	PF00384	PF00781	PF01186	PF01699	PF02234	PF02902	PF03637	PF04560	PF05240	PF06294	PF07529	PF08377
PF00075	PF00385	PF00782	PF01187	PF01702	PF02237	PF02910	PF03643	PF04561	PF05241	PF06297	PF07531	PF08383
PF00077	PF00386	PF00784	PF01189	PF01704	PF02238	PF02911	PF03645	PF04565	PF05250	PF06309	PF07533	PF08384
PF00080	PF00387	PF00786	PF01191	PF01709	PF02244	PF02913	PF03647	PF04566	PF05251	PF06311	PF07539	PF08385
PF00081	PF00388	PF00787	PF01192	PF01712	PF02245	PF02919	PF03651	PF04567	PF05253	PF06312	PF07540	PF08389
PF00082	PF00390	PF00789	PF01194	PF01713	PF02251	PF02920	PF03660	PF04568	PF05254	PF06320	PF07541	PF08390
PF00083	PF00393	PF00790	PF01198	PF01715	PF02252	PF02921	PF03661	PF04571	PF05255	PF06327	PF07542	PF08391
PF00084	PF00394	PF00791	PF01199	PF01722	PF02257	PF02922	PF03665	PF04572	PF05276	PF06328	PF07545	PF08393
PF00085	PF00396	PF00792	PF01200	PF01725	PF02259	PF02928	PF03666	PF04573	PF05279	PF06331	PF07546	PF08397
PF00088	PF00397	PF00794	PF01201	PF01729	PF02260	PF02931	PF03669	PF04579	PF05281	PF06333	PF07562	PF08399
PF00090	PF00398	PF00795	PF01202	PF01731	PF02262	PF02932	PF03670	PF04587	PF05282	PF06337	PF07565	PF08403
PF00091	PF00401	PF00797	PF01204	PF01733	PF02267	PF02933	PF03671	PF04588	PF05283	PF06345	PF07569	PF08412
PF00092	PF00402	PF00801	PF01207	PF01734	PF02268	PF02934	PF03676	PF04589	PF05287	PF06350	PF07571	PF08418
PF00093	PF00403	PF00804	PF01208	PF01735	PF02269	PF02935	PF03690	PF04592	PF05291	PF06365	PF07576	PF08420
PF00094	PF00405	PF00805	PF01210	PF01740	PF02270	PF02936	PF03694	PF04593	PF05292	PF06367	PF07593	PF08423

PF00095	PF00408	PF00806	PF01213	PF01743	PF02271	PF02937	PF03700	PF04597	PF05296	PF06372	PF07647	PF08424
PF00096	PF00410	PF00808	PF01214	PF01747	PF02272	PF02939	PF03712	PF04598	PF05300	PF06373	PF07648	PF08429
PF00098	PF00411	PF00809	PF01215	PF01749	PF02274	PF02946	PF03715	PF04614	PF05303	PF06374	PF07649	PF08430
PF00105	PF00412	PF00810	PF01216	PF01751	PF02275	PF02947	PF03719	PF04615	PF05324	PF06375	PF07650	PF08433
PF00106	PF00414	PF00812	PF01217	PF01753	PF02284	PF02953	PF03720	PF04617	PF05326	PF06384	PF07651	PF08441
PF00107	PF00416	PF00814	PF01218	PF01754	PF02285	PF02961	PF03721	PF04621	PF05328	PF06387	PF07657	PF08442
PF00108	PF00418	PF00817	PF01221	PF01756	PF02290	PF02965	PF03725	PF04627	PF05337	PF06391	PF07662	PF08447
PF00109	PF00420	PF00821	PF01222	PF01758	PF02291	PF02966	PF03726	PF04628	PF05347	PF06393	PF07670	PF08449
PF00111	PF00428	PF00822	PF01223	PF01759	PF02296	PF02969	PF03727	PF04629	PF05349	PF06396	PF07677	PF08450
PF00112	PF00433	PF00826	PF01227	PF01762	PF02301	PF02970	PF03730	PF04636	PF05350	PF06399	PF07678	PF08451
PF00113	PF00436	PF00827	PF01229	PF01765	PF02310	PF02971	PF03731	PF04641	PF05351	PF06400	PF07684	PF08454
PF00115	PF00439	PF00828	PF01230	PF01770	PF02312	PF02978	PF03735	PF04643	PF05355	PF06401	PF07687	PF08466
PF00116	PF00441	PF00831	PF01231	PF01773	PF02318	PF02984	PF03736	PF04644	PF05361	PF06418	PF07699	PF08473
PF00117	PF00444	PF00832	PF01233	PF01775	PF02319	PF02985	PF03747	PF04652	PF05362	PF06419	PF07700	PF08474
PF00118	PF00445	PF00833	PF01234	PF01776	PF02320	PF02990	PF03758	PF04658	PF05365	PF06424	PF07701	PF08477
PF00119	PF00446	PF00834	PF01237	PF01777	PF02330	PF02991	PF03764	PF04664	PF05366	PF06427	PF07703	PF08487
PF00120	PF00447	PF00835	PF01238	PF01778	PF02337	PF02996	PF03765	PF04667	PF05378	PF06428	PF07706	PF08490
PF00121	PF00448	PF00836	PF01239	PF01779	PF02338	PF03002	PF03770	PF04675	PF05383	PF06432	PF07710	PF08491
PF00123	PF00450	PF00837	PF01242	PF01780	PF02344	PF03006	PF03781	PF04679	PF05386	PF06441	PF07711	PF08492
PF00125	PF00452	PF00838	PF01243	PF01781	PF02345	PF03009	PF03792	PF04680	PF05392	PF06444	PF07716	PF08499
PF00129	PF00454	PF00839	PF01244	PF01784	PF02347	PF03016	PF03798	PF04683	PF05395	PF06446	PF07717	PF08504
PF00130	PF00458	PF00850	PF01245	PF01791	PF02348	PF03020	PF03803	PF04691	PF05399	PF06448	PF07718	PF08506
PF00131	PF00459	PF00852	PF01246	PF01794	PF02350	PF03024	PF03807	PF04692	PF05404	PF06455	PF07719	PF08510
PF00132	PF00462	PF00853	PF01247	PF01798	PF02351	PF03028	PF03810	PF04695	PF05405	PF06456	PF07722	PF08513
PF00133	PF00464	PF00855	PF01248	PF01799	PF02354	PF03029	PF03813	PF04696	PF05406	PF06459	PF07723	PF08514
PF00135	PF00466	PF00856	PF01249	PF01803	PF02359	PF03031	PF03815	PF04697	PF05422	PF06461	PF07724	PF08515
PF00136	PF00467	PF00858	PF01251	PF01805	PF02366	PF03034	PF03820	PF04698	PF05427	PF06462	PF07731	PF08516
PF00137	PF00468	PF00860	PF01253	PF01808	PF02372	PF03036	PF03821	PF04699	PF05428	PF06463	PF07732	PF08517
PF00143	PF00472	PF00861	PF01254	PF01812	PF02373	PF03039	PF03823	PF04704	PF05434	PF06464	PF07738	PF08518
PF00144	PF00473	PF00864	PF01255	PF01813	PF02374	PF03045	PF03826	PF04706	PF05438	PF06465	PF07741	PF08519
PF00145	PF00474	PF00865	PF01257	PF01821	PF02375	PF03051	PF03827	PF04707	PF05439	PF06466	PF07742	PF08523
PF00146	PF00476	PF00867	PF01259	PF01822	PF02376	PF03054	PF03828	PF04709	PF05450	PF06467	PF07743	PF08526

PF00149	PF00479	PF00868	PF01262	PF01823	PF02377	PF03055	PF03832	PF04710	PF05454	PF06468	PF07744	PF08527
PF00151	PF00480	PF00870	PF01263	PF01826	PF02383	PF03056	PF03834	PF04711	PF05456	PF06469	PF07748	PF08534
PF00152	PF00481	PF00879	PF01265	PF01833	PF02389	PF03061	PF03835	PF04716	PF05458	PF06470	PF07749	PF08540
PF00153	PF00485	PF00883	PF01267	PF01834	PF02390	PF03062	PF03836	PF04718	PF05460	PF06472	PF07763	PF08542
PF00155	PF00487	PF00884	PF01268	PF01835	PF02391	PF03068	PF03847	PF04719	PF05461	PF06479	PF07766	PF08544
PF00156	PF00488	PF00886	PF01269	PF01839	PF02394	PF03073	PF03849	PF04724	PF05463	PF06480	PF07767	
PF00157	PF00489	PF00887	PF01271	PF01840	PF02403	PF03074	PF03850	PF04727	PF05466	PF06482	PF07776	
PF00159	PF00490	PF00889	PF01273	PF01841	PF02404	PF03081	PF03868	PF04731	PF05470	PF06487	PF07778	
PF00160	PF00491	PF00890	PF01275	PF01842	PF02412	PF03088	PF03870	PF04732	PF05474	PF06512	PF07782	
PF00162	PF00493	PF00891	PF01279	PF01843	PF02423	PF03089	PF03871	PF04733	PF05477	PF06524	PF07807	
PF00163	PF00494	PF00895	PF01280	PF01846	PF02434	PF03091	PF03874	PF04739	PF05478	PF06534	PF07808	
PF00166	PF00498	PF00896	PF01281	PF01847	PF02436	PF03095	PF03875	PF04749	PF05483	PF06535	PF07810	
PF00169	PF00499	PF00899	PF01282	PF01852	PF02437	PF03096	PF03876	PF04750	PF05485	PF06540	PF07815	
PF00171	PF00503	PF00900	PF01283	PF01853	PF02441	PF03097	PF03896	PF04756	PF05486	PF06544	PF07830	
PF00173	PF00507	PF00903	PF01284	PF01857	PF02450	PF03099	PF03900	PF04757	PF05493	PF06546	PF07831	
PF00174	PF00510	PF00904	PF01285	PF01858	PF02453	PF03102	PF03901	PF04758	PF05495	PF06553	PF07834	
PF00175	PF00520	PF00907	PF01286	PF01868	PF02460	PF03104	PF03908	PF04762	PF05499	PF06554	PF07837	
PF00177	PF00521	PF00909	PF01287	PF01869	PF02466	PF03109	PF03909	PF04768	PF05502	PF06566	PF07842	
PF00178	PF00525	PF00916	PF01290	PF01871	PF02469	PF03114	PF03911	PF04774	PF05507	PF06567	PF07847	
PF00179	PF00530	PF00917	PF01291	PF01873	PF02485	PF03127	PF03914	PF04775	PF05510	PF06573	PF07850	
PF00180	PF00531	PF00918	PF01294	PF01875	PF02487	PF03128	PF03919	PF04777	PF05511	PF06581	PF07859	
PF00181	PF00534	PF00919	PF01299	PF01876	PF02493	PF03129	PF03920	PF04790	PF05517	PF06583	PF07883	
PF00184	PF00538	PF00923	PF01302	PF01896	PF02494	PF03131	PF03921	PF04795	PF05529	PF06588	PF07884	
PF00185	PF00542	PF00928	PF01315	PF01900	PF02513	PF03133	PF03931	PF04799	PF05557	PF06602	PF07885	
PF00186	PF00549	PF00929	PF01323	PF01907	PF02515	PF03134	PF03932	PF04800	PF05571	PF06607	PF07894	
PF00188	PF00550	PF00930	PF01327	PF01909	PF02516	PF03143	PF03939	PF04801	PF05572	PF06608	PF07904	
PF00189	PF00551	PF00931	PF01329	PF01912	PF02517	PF03144	PF03941	PF04803	PF05577	PF06614	PF07912	
PF00191	PF00552	PF00935	PF01331	PF01916	PF02525	PF03145	PF03943	PF04810	PF05586	PF06617	PF07915	
PF00193	PF00554	PF00939	PF01335	PF01918	PF02535	PF03146	PF03946	PF04811	PF05587	PF06621	PF07923	
PF00194	PF00557	PF00940	PF01336	PF01922	PF02536	PF03148	PF03947	PF04812	PF05600	PF06623	PF07926	
PF00198	PF00560	PF00941	PF01342	PF01923	PF02538	PF03149	PF03949	PF04813	PF05604	PF06625	PF07928	
PF00199	PF00562	PF00953	PF01347	PF01926	PF02544	PF03151	PF03950	PF04814	PF05622	PF06631	PF07933	

PF00201	PF00564	PF00955	PF01351	PF01928	PF02545	PF03152	PF03952	PF04815	PF05640	PF06632	PF07934
PF00202	PF00566	PF00956	PF01354	PF01929	PF02567	PF03153	PF03953	PF04818	PF05641	PF06637	PF07941
PF00203	PF00568	PF00957	PF01363	PF01937	PF02574	PF03155	PF03954	PF04819	PF05645	PF06638	PF07942
PF00204	PF00569	PF00958	PF01365	PF01938	PF02578	PF03159	PF03957	PF04821	PF05648	PF06645	PF07959
PF00205	PF00570	PF00962	PF01369	PF01963	PF02580	PF03160	PF03966	PF04822	PF05649	PF06650	PF07965
PF00206	PF00571	PF00965	PF01379	PF01965	PF02585	PF03165	PF03981	PF04824	PF05652	PF06657	PF07966
PF00207	PF00572	PF00969	PF01380	PF01966	PF02594	PF03166	PF03991	PF04825	PF05653	PF06662	PF07967
PF00208	PF00573	PF00970	PF01381	PF01967	PF02597	PF03167	PF03992	PF04831	PF05669	PF06663	PF07970
PF00209	PF00574	PF00975	PF01387	PF01968	PF02602	PF03171	PF03997	PF04836	PF05670	PF06668	PF07973
PF00210	PF00575	PF00976	PF01388	PF01974	PF02607	PF03172	PF03998	PF04840	PF05676	PF06677	PF07974
PF00211	PF00576	PF00984	PF01390	PF01979	PF02617	PF03177	PF04003	PF04841	PF05679	PF06699	PF07975
PF00212	PF00578	PF00988	PF01392	PF01981	PF02628	PF03178	PF04006	PF04845	PF05680	PF06701	PF07978
PF00213	PF00579	PF00992	PF01393	PF01984	PF02629	PF03179	PF04031	PF04847	PF05682	PF06702	PF07985
PF00214	PF00581	PF00993	PF01394	PF01990	PF02630	PF03184	PF04032	PF04855	PF05693	PF06703	PF07986
PF00215	PF00584	PF00994	PF01396	PF01991	PF02637	PF03185	PF04037	PF04856	PF05694	PF06726	PF07988
PF00217	PF00586	PF00996	PF01398	PF01992	PF02666	PF03188	PF04042	PF04858	PF05699	PF06728	PF07989
PF00219	PF00587	PF00997	PF01399	PF01997	PF02668	PF03194	PF04044	PF04868	PF05700	PF06729	PF08005
PF00220	PF00590	PF00999	PF01400	PF02002	PF02671	PF03199	PF04045	PF04869	PF05706	PF06732	PF08016
PF00221	PF00591	PF01000	PF01401	PF02005	PF02678	PF03200	PF04046	PF04871	PF05712	PF06733	PF08022
PF00224	PF00594	PF01007	PF01403	PF02008	PF02686	PF03208	PF04047	PF04874	PF05715	PF06741	PF08030
PF00225	PF00596	PF01008	PF01404	PF02012	PF02709	PF03215	PF04049	PF04882	PF05719	PF06743	PF08033
PF00227	PF00605	PF01012	PF01408	PF02014	PF02724	PF03223	PF04050	PF04886	PF05721	PF06747	PF08035
PF00229	PF00607	PF01015	PF01409	PF02017	PF02727	PF03224	PF04051	PF04900	PF05724	PF06752	PF08038
PF00230	PF00609	PF01016	PF01410	PF02019	PF02728	PF03226	PF04053	PF04901	PF05726	PF06773	PF08039
PF00231	PF00610	PF01017	PF01411	PF02020	PF02729	PF03227	PF04055	PF04902	PF05729	PF06775	PF08040
PF00232	PF00611	PF01018	PF01412	PF02022	PF02731	PF03232	PF04056	PF04904	PF05731	PF06777	PF08059
PF00233	PF00612	PF01020	PF01413	PF02025	PF02732	PF03234	PF04057	PF04905	PF05735	PF06783	PF08060
PF00235	PF00613	PF01023	PF01414	PF02026	PF02735	PF03247	PF04062	PF04908	PF05739	PF06784	PF08061
PF00236	PF00614	PF01025	PF01415	PF02036	PF02736	PF03248	PF04065	PF04909	PF05741	PF06807	PF08062
PF00237	PF00616	PF01026	PF01416	PF02037	PF02737	PF03250	PF04068	PF04910	PF05743	PF06809	PF08063
PF00238	PF00617	PF01027	PF01417	PF02038	PF02738	PF03253	PF04072	PF04912	PF05746	PF06814	PF08064
PF00241	PF00618	PF01028	PF01419	PF02039	PF02740	PF03256	PF04081	PF04921	PF05753	PF06816	PF08065

PF00243	PF00619	PF01030	PF01421	PF02044	PF02743	PF03259	PF04083	PF04923	PF05756	PF06817	PF08066
PF00244	PF00620	PF01031	PF01422	PF02046	PF02744	PF03261	PF04084	PF04926	PF05760	PF06818	PF08067
PF00245	PF00621	PF01033	PF01424	PF02055	PF02747	PF03265	PF04086	PF04928	PF05761	PF06821	PF08068
PF00246	PF00622	PF01035	PF01425	PF02057	PF02749	PF03266	PF04088	PF04934	PF05764	PF06825	PF08069
PF00248	PF00623	PF01039	PF01426	PF02058	PF02750	PF03271	PF04089	PF04935	PF05773	PF06828	PF08070
PF00249	PF00627	PF01040	PF01428	PF02059	PF02751	PF03285	PF04091	PF04938	PF05778	PF06831	PF08071
PF00250	PF00629	PF01042	PF01429	PF02064	PF02752	PF03297	PF04095	PF04939	PF05781	PF06839	PF08072
PF00253	PF00631	PF01048	PF01431	PF02070	PF02755	PF03298	PF04096	PF04950	PF05782	PF06858	PF08073
PF00255	PF00632	PF01053	PF01432	PF02071	PF02757	PF03299	PF04097	PF04952	PF05783	PF06870	PF08074
PF00256	PF00633	PF01055	PF01434	PF02072	PF02758	PF03301	PF04098	PF04959	PF05786	PF06875	PF08075
PF00258	PF00634	PF01056	PF01435	PF02077	PF02759	PF03308	PF04099	PF04960	PF05790	PF06881	PF08079
PF00260	PF00635	PF01058	PF01436	PF02078	PF02760	PF03311	PF04103	PF04961	PF05793	PF06883	PF08080
PF00261	PF00636	PF01059	PF01437	PF02079	PF02761	PF03321	PF04104	PF04968	PF05804	PF06886	PF08081
PF00262	PF00637	PF01061	PF01448	PF02083	PF02762	PF03332	PF04111	PF04969	PF05805	PF06905	PF08082
PF00264	PF00638	PF01062	PF01451	PF02089	PF02765	PF03345	PF04113	PF04970	PF05806	PF06907	PF08083
PF00265	PF00639	PF01063	PF01459	PF02093	PF02769	PF03351	PF04114	PF04979	PF05817	PF06910	PF08084
PF00266	PF00642	PF01064	PF01462	PF02096	PF02770	PF03357	PF04117	PF04983	PF05821	PF06916	PF08122
PF00268	PF00643	PF01066	PF01463	PF02099	PF02771	PF03359	PF04118	PF04988	PF05822	PF06920	PF08123
PF00273	PF00644	PF01067	PF01464	PF02100	PF02775	PF03360	PF04121	PF04990	PF05824	PF06936	PF08127
PF00274	PF00645	PF01068	PF01465	PF02101	PF02776	PF03366	PF04124	PF04992	PF05825	PF06937	PF08142
PF00276	PF00646	PF01070	PF01466	PF02104	PF02777	PF03367	PF04127	PF04997	PF05826	PF06941	PF08143
PF00277	PF00647	PF01071	PF01467	PF02106	PF02779	PF03368	PF04128	PF05000	PF05827	PF06951	PF08144
PF00278	PF00648	PF01073	PF01469	PF02109	PF02780	PF03370	PF04130	PF05001	PF05832	PF06954	PF08145
PF00281	PF00653	PF01074	PF01470	PF02114	PF02781	PF03372	PF04132	PF05002	PF05835	PF06957	PF08146
PF00285	PF00654	PF01079	PF01472	PF02115	PF02782	PF03378	PF04133	PF05004	PF05843	PF06959	PF08147
PF00287	PF00656	PF01080	PF01477	PF02121	PF02784	PF03388	PF04135	PF05005	PF05856	PF06963	PF08148
PF00288	PF00657	PF01082	PF01479	PF02126	PF02785	PF03398	PF04136	PF05008	PF05863	PF06978	PF08149
PF00289	PF00658	PF01084	PF01480	PF02127	PF02786	PF03399	PF04137	PF05010	PF05871	PF06984	PF08150
PF00291	PF00659	PF01085	PF01483	PF02130	PF02787	PF03402	PF04140	PF05014	PF05873	PF06990	PF08151
PF00292	PF00662	PF01086	PF01485	PF02134	PF02789	PF03403	PF04142	PF05019	PF05875	PF06991	PF08152
PF00293	PF00665	PF01087	PF01490	PF02135	PF02790	PF03404	PF04144	PF05020	PF05881	PF07001	PF08153
PF00294	PF00666	PF01088	PF01491	PF02136	PF02791	PF03414	PF04145	PF05021	PF05889	PF07002	PF08154

PF00297	PF00667	PF01090	PF01496	PF02137	PF02792	PF03446	PF04146	PF05022	PF05890	PF07010	PF08155
PF00298	PF00670	PF01091	PF01501	PF02138	PF02793	PF03450	PF04147	PF05024	PF05902	PF07019	PF08156
PF00300	PF00673	PF01092	PF01504	PF02140	PF02798	PF03451	PF04152	PF05026	PF05903	PF07034	PF08157
PF00303	PF00675	PF01093	PF01505	PF02141	PF02799	PF03453	PF04153	PF05028	PF05918	PF07035	PF08159
PF00305	PF00676	PF01096	PF01509	PF02142	PF02800	PF03454	PF04157	PF05029	PF05922	PF07047	PF08160
PF00306	PF00679	PF01099	PF01510	PF02145	PF02801	PF03455	PF04177	PF05030	PF05923	PF07074	PF08161
PF00307	PF00682	PF01101	PF01512	PF02146	PF02803	PF03456	PF04191	PF05033	PF05924	PF07084	PF08162
PF00310	PF00683	PF01103	PF01513	PF02148	PF02806	PF03462	PF04192	PF05038	PF05937	PF07093	PF08163
PF00312	PF00684	PF01105	PF01529	PF02149	PF02807	PF03463	PF04193	PF05039	PF05956	PF07111	PF08164
PF00313	PF00685	PF01106	PF01530	PF02150	PF02809	PF03464	PF04194	PF05040	PF05964	PF07140	PF08165
PF00316	PF00686	PF01109	PF01531	PF02157	PF02812	PF03465	PF04201	PF05041	PF05965	PF07142	PF08168
PF00317	PF00687	PF01110	PF01532	PF02159	PF02815	PF03467	PF04209	PF05044	PF05967	PF07145	PF08169
PF00318	PF00688	PF01111	PF01534	PF02161	PF02816	PF03477	PF04212	PF05046	PF05972	PF07147	PF08170
PF00319	PF00689	PF01112	PF01535	PF02165	PF02817	PF03483	PF04218	PF05047	PF05978	PF07156	PF08180
PF00320	PF00690	PF01114	PF01536	PF02167	PF02820	PF03484	PF04253	PF05051	PF05983	PF07159	PF08185
PF00322	PF00692	PF01115	PF01545	PF02170	PF02822	PF03485	PF04258	PF05053	PF05995	PF07163	PF08198

**Vertebrate** Pfam ID												
PF00005	PF00342	PF00753	PF01162	PF01633	PF02229	PF02910	PF03630	PF04548	PF05240	PF06248	PF07524	PF08327
PF00006	PF00343	PF00755	PF01163	PF01642	PF02230	PF02911	PF03635	PF04549	PF05241	PF06268	PF07525	PF08332
PF00009	PF00344	PF00756	PF01166	PF01652	PF02233	PF02913	PF03637	PF04553	PF05242	PF06292	PF07527	PF08333
PF00010	PF00346	PF00758	PF01167	PF01655	PF02234	PF02919	PF03643	PF04557	PF05250	PF06294	PF07528	PF08336
PF00013	PF00347	PF00762	PF01168	PF01661	PF02237	PF02920	PF03645	PF04558	PF05251	PF06297	PF07529	PF08337
PF00014	PF00348	PF00763	PF01169	PF01663	PF02238	PF02921	PF03647	PF04560	PF05253	PF06309	PF07531	PF08344
PF00017	PF00349	PF00764	PF01170	PF01667	PF02244	PF02922	PF03651	PF04561	PF05254	PF06311	PF07533	PF08351
PF00019	PF00350	PF00766	PF01172	PF01669	PF02245	PF02928	PF03660	PF04565	PF05255	PF06312	PF07539	PF08357
PF00024	PF00351	PF00769	PF01175	PF01687	PF02251	PF02931	PF03661	PF04566	PF05276	PF06320	PF07540	PF08365
PF00025	PF00352	PF00773	PF01176	PF01694	PF02252	PF02932	PF03665	PF04567	PF05279	PF06327	PF07541	PF08368
PF00026	PF00354	PF00774	PF01179	PF01699	PF02257	PF02933	PF03666	PF04568	PF05281	PF06328	PF07542	PF08373
PF00027	PF00355	PF00777	PF01180	PF01702	PF02259	PF02934	PF03669	PF04571	PF05282	PF06331	PF07545	PF08374
PF00029	PF00357	PF00778	PF01182	PF01704	PF02260	PF02935	PF03670	PF04572	PF05283	PF06333	PF07546	PF08375
PF00030	PF00362	PF00779	PF01187	PF01709	PF02262	PF02936	PF03671	PF04573	PF05287	PF06337	PF07562	PF08377
PF00032	PF00364	PF00780	PF01189	PF01712	PF02267	PF02937	PF03676	PF04579	PF05291	PF06345	PF07565	PF08383
PF00034	PF00365	PF00781	PF01191	PF01713	PF02268	PF02939	PF03690	PF04587	PF05292	PF06350	PF07569	PF08384
PF00037	PF00366	PF00782	PF01192	PF01715	PF02269	PF02946	PF03694	PF04588	PF05296	PF06365	PF07571	PF08385
PF00039	PF00368	PF00784	PF01194	PF01722	PF02270	PF02947	PF03700	PF04589	PF05298	PF06367	PF07576	PF08389
PF00040	PF00370	PF00786	PF01198	PF01725	PF02271	PF02948	PF03712	PF04592	PF05300	PF06372	PF07593	PF08390
PF00044	PF00375	PF00787	PF01199	PF01729	PF02272	PF02953	PF03715	PF04593	PF05303	PF06373	PF07646	PF08393
PF00045	PF00377	PF00789	PF01200	PF01731	PF02274	PF02961	PF03719	PF04597	PF05324	PF06374	PF07647	PF08397
PF00046	PF00378	PF00790	PF01201	PF01733	PF02275	PF02965	PF03720	PF04598	PF05326	PF06375	PF07649	PF08399
PF00048	PF00382	PF00791	PF01202	PF01734	PF02284	PF02966	PF03721	PF04614	PF05328	PF06384	PF07650	PF08403
PF00050	PF00383	PF00792	PF01204	PF01735	PF02285	PF02969	PF03725	PF04615	PF05337	PF06387	PF07651	PF08412
PF00051	PF00384	PF00795	PF01207	PF01740	PF02290	PF02970	PF03726	PF04617	PF05347	PF06391	PF07657	PF08416
PF00052	PF00385	PF00797	PF01208	PF01743	PF02291	PF02971	PF03727	PF04621	PF05349	PF06393	PF07662	PF08418
PF00054	PF00386	PF00801	PF01210	PF01747	PF02296	PF02978	PF03730	PF04627	PF05350	PF06396	PF07670	PF08420
PF00055	PF00388	PF00804	PF01213	PF01749	PF02301	PF02984	PF03731	PF04628	PF05351	PF06399	PF07677	PF08423
PF00056	PF00390	PF00806	PF01214	PF01751	PF02310	PF02985	PF03735	PF04629	PF05355	PF06400	PF07678	PF08424

PF00058	PF00393	PF00808	PF01215	PF01753	PF02312	PF02990	PF03736	PF04636	PF05362	PF06401	PF07684	PF08429
PF00060	PF00394	PF00809	PF01216	PF01754	PF02318	PF02991	PF03747	PF04641	PF05365	PF06403	PF07687	PF08430
PF00062	PF00396	PF00810	PF01217	PF01756	PF02319	PF02994	PF03758	PF04643	PF05366	PF06418	PF07699	PF08433
PF00066	PF00397	PF00811	PF01218	PF01758	PF02320	PF02996	PF03762	PF04652	PF05378	PF06419	PF07701	PF08434
PF00068	PF00398	PF00812	PF01221	PF01759	PF02330	PF03002	PF03764	PF04658	PF05383	PF06424	PF07703	PF08441
PF00072	PF00401	PF00814	PF01222	PF01762	PF02337	PF03006	PF03765	PF04664	PF05386	PF06427	PF07706	PF08442
PF00074	PF00402	PF00817	PF01223	PF01765	PF02338	PF03009	PF03770	PF04667	PF05392	PF06428	PF07710	PF08447
PF00075	PF00403	PF00819	PF01227	PF01770	PF02344	PF03016	PF03781	PF04675	PF05395	PF06432	PF07711	PF08449
PF00077	PF00408	PF00821	PF01229	PF01773	PF02345	PF03020	PF03792	PF04679	PF05399	PF06441	PF07716	PF08450
PF00080	PF00410	PF00822	PF01230	PF01775	PF02347	PF03024	PF03798	PF04680	PF05404	PF06446	PF07717	PF08451
PF00081	PF00411	PF00826	PF01231	PF01776	PF02348	PF03028	PF03803	PF04683	PF05405	PF06448	PF07718	PF08454
PF00083	PF00412	PF00827	PF01233	PF01777	PF02350	PF03029	PF03807	PF04691	PF05406	PF06455	PF07719	PF08466
PF00084	PF00414	PF00828	PF01234	PF01778	PF02351	PF03031	PF03810	PF04692	PF05418	PF06456	PF07720	PF08473
PF00085	PF00416	PF00831	PF01237	PF01779	PF02354	PF03032	PF03813	PF04695	PF05422	PF06459	PF07722	PF08474
PF00087	PF00418	PF00832	PF01238	PF01780	PF02359	PF03034	PF03815	PF04696	PF05427	PF06461	PF07723	PF08477
PF00088	PF00420	PF00833	PF01239	PF01781	PF02366	PF03036	PF03820	PF04697	PF05428	PF06462	PF07724	PF08487
PF00090	PF00428	PF00834	PF01242	PF01783	PF02372	PF03039	PF03821	PF04698	PF05434	PF06463	PF07731	PF08490
PF00091	PF00433	PF00835	PF01243	PF01784	PF02373	PF03045	PF03823	PF04699	PF05438	PF06464	PF07738	PF08491
PF00092	PF00436	PF00836	PF01244	PF01791	PF02374	PF03051	PF03826	PF04704	PF05439	PF06465	PF07741	PF08492
PF00093	PF00439	PF00837	PF01245	PF01794	PF02375	PF03054	PF03827	PF04706	PF05450	PF06466	PF07742	PF08499
PF00094	PF00441	PF00838	PF01246	PF01798	PF02376	PF03055	PF03828	PF04707	PF05454	PF06467	PF07743	PF08504
PF00095	PF00444	PF00839	PF01247	PF01799	PF02377	PF03056	PF03832	PF04709	PF05456	PF06468	PF07744	PF08506
PF00096	PF00445	PF00850	PF01248	PF01803	PF02383	PF03061	PF03834	PF04710	PF05458	PF06469	PF07748	PF08510
PF00098	PF00446	PF00852	PF01249	PF01805	PF02389	PF03062	PF03835	PF04711	PF05460	PF06470	PF07749	PF08513
PF00105	PF00447	PF00853	PF01251	PF01808	PF02390	PF03068	PF03836	PF04716	PF05461	PF06472	PF07763	PF08514
PF00106	PF00448	PF00855	PF01253	PF01812	PF02391	PF03073	PF03847	PF04718	PF05463	PF06479	PF07766	PF08515
PF00107	PF00450	PF00856	PF01254	PF01813	PF02403	PF03074	PF03849	PF04719	PF05466	PF06480	PF07767	PF08516
PF00108	PF00452	PF00858	PF01255	PF01821	PF02404	PF03081	PF03850	PF04724	PF05470	PF06482	PF07776	PF08517
PF00109	PF00454	PF00860	PF01257	PF01822	PF02412	PF03088	PF03868	PF04727	PF05477	PF06487	PF07778	PF08518
PF00111	PF00458	PF00861	PF01259	PF01823	PF02422	PF03089	PF03870	PF04731	PF05478	PF06512	PF07782	PF08519
PF00112	PF00459	PF00864	PF01262	PF01833	PF02423	PF03091	PF03871	PF04732	PF05483	PF06524	PF07807	PF08523
PF00113	PF00462	PF00865	PF01263	PF01835	PF02434	PF03095	PF03874	PF04733	PF05485	PF06534	PF07808	PF08526

PF00115	PF00464	PF00867	PF01265	PF01839	PF02436	PF03096	PF03875	PF04739	PF05486	PF06535	PF07810	PF08527
PF00116	PF00466	PF00868	PF01267	PF01840	PF02437	PF03097	PF03876	PF04749	PF05493	PF06540	PF07815	PF08534
PF00117	PF00467	PF00870	PF01268	PF01841	PF02441	PF03099	PF03896	PF04750	PF05495	PF06544	PF07818	PF08540
PF00118	PF00468	PF00875	PF01269	PF01842	PF02450	PF03102	PF03900	PF04756	PF05499	PF06546	PF07830	PF08542
PF00119	PF00472	PF00879	PF01271	PF01843	PF02453	PF03104	PF03901	PF04757	PF05502	PF06553	PF07831	PF08544
PF00120	PF00473	PF00883	PF01273	PF01846	PF02460	PF03109	PF03908	PF04758	PF05507	PF06554	PF07834	
PF00121	PF00474	PF00884	PF01275	PF01847	PF02466	PF03127	PF03909	PF04762	PF05510	PF06566	PF07837	
PF00123	PF00476	PF00886	PF01279	PF01852	PF02469	PF03128	PF03911	PF04768	PF05517	PF06567	PF07842	
PF00125	PF00479	PF00887	PF01280	PF01853	PF02485	PF03129	PF03914	PF04774	PF05529	PF06573	PF07847	
PF00129	PF00480	PF00889	PF01281	PF01857	PF02487	PF03131	PF03919	PF04775	PF05557	PF06581	PF07850	
PF00130	PF00481	PF00890	PF01282	PF01858	PF02493	PF03133	PF03920	PF04777	PF05571	PF06583	PF07859	
PF00131	PF00485	PF00891	PF01283	PF01868	PF02494	PF03134	PF03921	PF04790	PF05572	PF06588	PF07883	
PF00132	PF00487	PF00895	PF01284	PF01869	PF02513	PF03143	PF03931	PF04795	PF05577	PF06602	PF07884	
PF00135	PF00488	PF00896	PF01285	PF01871	PF02515	PF03144	PF03932	PF04799	PF05586	PF06608	PF07885	
PF00136	PF00489	PF00899	PF01286	PF01873	PF02516	PF03145	PF03939	PF04800	PF05587	PF06614	PF07894	
PF00137	PF00490	PF00900	PF01287	PF01875	PF02517	PF03146	PF03940	PF04801	PF05600	PF06621	PF07904	
PF00144	PF00491	PF00903	PF01290	PF01876	PF02525	PF03148	PF03941	PF04803	PF05604	PF06623	PF07912	
PF00145	PF00493	PF00904	PF01291	PF01896	PF02535	PF03149	PF03943	PF04810	PF05609	PF06625	PF07915	
PF00146	PF00494	PF00907	PF01294	PF01900	PF02536	PF03151	PF03946	PF04811	PF05622	PF06631	PF07923	
PF00149	PF00498	PF00909	PF01299	PF01907	PF02538	PF03152	PF03947	PF04812	PF05640	PF06632	PF07926	
PF00151	PF00499	PF00916	PF01302	PF01909	PF02544	PF03153	PF03949	PF04813	PF05641	PF06637	PF07928	
PF00152	PF00503	PF00917	PF01315	PF01912	PF02545	PF03155	PF03950	PF04814	PF05645	PF06638	PF07933	
PF00153	PF00507	PF00919	PF01323	PF01916	PF02567	PF03159	PF03952	PF04815	PF05648	PF06645	PF07934	
PF00156	PF00510	PF00923	PF01327	PF01918	PF02574	PF03160	PF03953	PF04818	PF05649	PF06650	PF07941	
PF00157	PF00521	PF00928	PF01329	PF01922	PF02578	PF03165	PF03954	PF04819	PF05652	PF06657	PF07942	
PF00159	PF00525	PF00929	PF01331	PF01923	PF02580	PF03166	PF03957	PF04821	PF05653	PF06662	PF07959	
PF00160	PF00531	PF00930	PF01335	PF01926	PF02585	PF03167	PF03966	PF04822	PF05669	PF06663	PF07965	
PF00162	PF00534	PF00931	PF01336	PF01928	PF02594	PF03171	PF03981	PF04824	PF05670	PF06668	PF07966	
PF00163	PF00538	PF00932	PF01342	PF01929	PF02597	PF03172	PF03991	PF04825	PF05676	PF06677	PF07967	
PF00166	PF00542	PF00935	PF01347	PF01937	PF02602	PF03177	PF03992	PF04831	PF05679	PF06699	PF07970	
PF00169	PF00549	PF00939	PF01351	PF01938	PF02607	PF03178	PF03997	PF04836	PF05680	PF06701	PF07973	
PF00171	PF00550	PF00940	PF01354	PF01958	PF02617	PF03179	PF03998	PF04840	PF05682	PF06702	PF07974	

PF00173	PF00551	PF00941	PF01363	PF01963	PF02628	PF03184	PF04003	PF04841	PF05693	PF06703	PF07975
PF00174	PF00552	PF00953	PF01365	PF01965	PF02629	PF03185	PF04006	PF04845	PF05694	PF06726	PF07978
PF00175	PF00554	PF00955	PF01369	PF01966	PF02630	PF03188	PF04030	PF04847	PF05699	PF06728	PF07985
PF00177	PF00557	PF00956	PF01379	PF01967	PF02637	PF03194	PF04031	PF04855	PF05700	PF06729	PF07986
PF00178	PF00560	PF00957	PF01380	PF01968	PF02666	PF03199	PF04032	PF04856	PF05706	PF06732	PF07988
PF00179	PF00562	PF00958	PF01381	PF01974	PF02668	PF03200	PF04037	PF04858	PF05712	PF06733	PF07989
PF00180	PF00564	PF00962	PF01382	PF01979	PF02671	PF03208	PF04042	PF04868	PF05715	PF06741	PF08005
PF00181	PF00566	PF00965	PF01387	PF01981	PF02678	PF03215	PF04044	PF04869	PF05719	PF06743	PF08016
PF00184	PF00568	PF00969	PF01388	PF01984	PF02709	PF03223	PF04045	PF04871	PF05721	PF06747	PF08022
PF00185	PF00569	PF00970	PF01390	PF01990	PF02724	PF03224	PF04046	PF04874	PF05724	PF06752	PF08023
PF00186	PF00570	PF00975	PF01392	PF01991	PF02727	PF03226	PF04047	PF04882	PF05726	PF06753	PF08030
PF00188	PF00571	PF00984	PF01393	PF01992	PF02728	PF03227	PF04049	PF04886	PF05729	PF06773	PF08033
PF00189	PF00572	PF00988	PF01394	PF01997	PF02729	PF03232	PF04050	PF04900	PF05731	PF06775	PF08035
PF00191	PF00573	PF00992	PF01396	PF02002	PF02731	PF03234	PF04051	PF04901	PF05735	PF06777	PF08038
PF00193	PF00574	PF00993	PF01398	PF02005	PF02732	PF03247	PF04053	PF04902	PF05739	PF06783	PF08039
PF00194	PF00575	PF00994	PF01399	PF02008	PF02735	PF03248	PF04055	PF04904	PF05741	PF06784	PF08040
PF00198	PF00576	PF00996	PF01400	PF02012	PF02736	PF03250	PF04056	PF04905	PF05742	PF06807	PF08059
PF00199	PF00578	PF00999	PF01401	PF02014	PF02737	PF03253	PF04057	PF04908	PF05743	PF06809	PF08060
PF00201	PF00579	PF01000	PF01403	PF02017	PF02738	PF03256	PF04062	PF04909	PF05746	PF06814	PF08061
PF00202	PF00581	PF01007	PF01404	PF02019	PF02740	PF03259	PF04065	PF04910	PF05753	PF06816	PF08062
PF00203	PF00583	PF01008	PF01408	PF02020	PF02743	PF03261	PF04068	PF04912	PF05756	PF06817	PF08063
PF00204	PF00584	PF01012	PF01409	PF02022	PF02744	PF03265	PF04072	PF04921	PF05760	PF06818	PF08064
PF00205	PF00586	PF01014	PF01410	PF02025	PF02747	PF03266	PF04081	PF04923	PF05761	PF06821	PF08065
PF00206	PF00587	PF01015	PF01411	PF02026	PF02749	PF03271	PF04083	PF04926	PF05764	PF06825	PF08066
PF00207	PF00590	PF01016	PF01412	PF02036	PF02750	PF03285	PF04084	PF04934	PF05773	PF06828	PF08067
PF00208	PF00591	PF01017	PF01413	PF02037	PF02751	PF03297	PF04086	PF04935	PF05778	PF06831	PF08068
PF00209	PF00594	PF01018	PF01414	PF02039	PF02752	PF03299	PF04088	PF04938	PF05781	PF06839	PF08069
PF00210	PF00596	PF01020	PF01415	PF02044	PF02755	PF03301	PF04089	PF04939	PF05782	PF06858	PF08070
PF00211	PF00605	PF01023	PF01416	PF02046	PF02757	PF03308	PF04091	PF04950	PF05783	PF06870	PF08071
PF00213	PF00609	PF01025	PF01417	PF02055	PF02758	PF03311	PF04095	PF04952	PF05786	PF06875	PF08072
PF00214	PF00610	PF01026	PF01419	PF02057	PF02759	PF03321	PF04096	PF04959	PF05790	PF06881	PF08073
PF00215	PF00611	PF01027	PF01421	PF02058	PF02760	PF03332	PF04097	PF04960	PF05793	PF06886	PF08074

PF00217	PF00612	PF01028	PF01422	PF02059	PF02761	PF03345	PF04098	PF04961	PF05794	PF06905	PF08075
PF00219	PF00613	PF01030	PF01424	PF02064	PF02762	PF03351	PF04099	PF04968	PF05804	PF06907	PF08079
PF00220	PF00614	PF01031	PF01425	PF02071	PF02765	PF03357	PF04103	PF04969	PF05805	PF06910	PF08080
PF00221	PF00616	PF01033	PF01426	PF02077	PF02769	PF03359	PF04104	PF04970	PF05806	PF06916	PF08081
PF00224	PF00617	PF01035	PF01428	PF02078	PF02770	PF03360	PF04111	PF04979	PF05808	PF06920	PF08082
PF00225	PF00618	PF01039	PF01431	PF02079	PF02771	PF03366	PF04113	PF04983	PF05817	PF06936	PF08083
PF00227	PF00620	PF01040	PF01432	PF02083	PF02775	PF03367	PF04114	PF04988	PF05821	PF06937	PF08084
PF00230	PF00621	PF01042	PF01434	PF02089	PF02776	PF03368	PF04117	PF04990	PF05822	PF06941	PF08103
PF00231	PF00623	PF01048	PF01435	PF02093	PF02777	PF03370	PF04118	PF04992	PF05824	PF06951	PF08110
PF00232	PF00626	PF01053	PF01436	PF02096	PF02779	PF03372	PF04121	PF05000	PF05826	PF06954	PF08121
PF00233	PF00627	PF01055	PF01437	PF02099	PF02780	PF03378	PF04124	PF05001	PF05827	PF06957	PF08122
PF00235	PF00629	PF01058	PF01448	PF02100	PF02781	PF03388	PF04127	PF05002	PF05832	PF06959	PF08123
PF00236	PF00631	PF01059	PF01451	PF02101	PF02782	PF03398	PF04128	PF05004	PF05835	PF06963	PF08127
PF00237	PF00632	PF01061	PF01459	PF02104	PF02784	PF03399	PF04130	PF05005	PF05843	PF06970	PF08131
PF00238	PF00635	PF01062	PF01462	PF02106	PF02785	PF03402	PF04132	PF05008	PF05856	PF06978	PF08142
PF00241	PF00636	PF01063	PF01463	PF02109	PF02786	PF03403	PF04133	PF05010	PF05863	PF06984	PF08143
PF00243	PF00637	PF01064	PF01464	PF02114	PF02787	PF03404	PF04135	PF05014	PF05871	PF06990	PF08144
PF00244	PF00638	PF01066	PF01465	PF02115	PF02789	PF03414	PF04136	PF05019	PF05873	PF06991	PF08145
PF00245	PF00639	PF01067	PF01466	PF02121	PF02791	PF03441	PF04137	PF05020	PF05875	PF06994	PF08146
PF00246	PF00642	PF01068	PF01467	PF02126	PF02792	PF03446	PF04140	PF05021	PF05881	PF07001	PF08147
PF00248	PF00643	PF01070	PF01469	PF02127	PF02793	PF03447	PF04142	PF05022	PF05889	PF07002	PF08148
PF00249	PF00644	PF01071	PF01470	PF02130	PF02799	PF03450	PF04144	PF05024	PF05890	PF07010	PF08149
PF00250	PF00645	PF01073	PF01472	PF02134	PF02800	PF03451	PF04145	PF05026	PF05902	PF07019	PF08150
PF00253	PF00646	PF01074	PF01477	PF02135	PF02801	PF03453	PF04146	PF05028	PF05903	PF07034	PF08151
PF00255	PF00647	PF01079	PF01479	PF02136	PF02803	PF03454	PF04147	PF05029	PF05918	PF07035	PF08152
PF00256	PF00648	PF01080	PF01480	PF02137	PF02806	PF03455	PF04152	PF05030	PF05922	PF07047	PF08153
PF00258	PF00653	PF01082	PF01483	PF02138	PF02807	PF03456	PF04153	PF05033	PF05923	PF07074	PF08154
PF00260	PF00654	PF01084	PF01485	PF02140	PF02809	PF03462	PF04157	PF05038	PF05924	PF07084	PF08155
PF00261	PF00656	PF01085	PF01490	PF02141	PF02812	PF03463	PF04177	PF05039	PF05937	PF07093	PF08156
PF00262	PF00657	PF01086	PF01491	PF02142	PF02815	PF03464	PF04191	PF05040	PF05956	PF07111	PF08157
PF00264	PF00658	PF01087	PF01496	PF02146	PF02816	PF03465	PF04192	PF05041	PF05964	PF07140	PF08159
PF00265	PF00659	PF01088	PF01501	PF02148	PF02817	PF03467	PF04193	PF05044	PF05965	PF07142	PF08160

PF00266	PF00662	PF01090	PF01504	PF02149	PF02820	PF03477	PF04194	PF05046	PF05967	PF07145	PF08161
PF00268	PF00666	PF01091	PF01505	PF02150	PF02822	PF03483	PF04201	PF05047	PF05972	PF07147	PF08162
PF00271	PF00667	PF01092	PF01509	PF02157	PF02823	PF03484	PF04209	PF05051	PF05978	PF07156	PF08163
PF00273	PF00670	PF01093	PF01510	PF02165	PF02824	PF03485	PF04212	PF05053	PF05983	PF07159	PF08164
PF00274	PF00673	PF01096	PF01512	PF02167	PF02825	PF03487	PF04218	PF05060	PF05995	PF07163	PF08165
PF00276	PF00675	PF01101	PF01513	PF02170	PF02826	PF03489	PF04253	PF05063	PF05997	PF07169	PF08168
PF00278	PF00676	PF01103	PF01529	PF02171	PF02827	PF03491	PF04258	PF05064	PF06001	PF07175	PF08169
PF00281	PF00679	PF01105	PF01530	PF02172	PF02828	PF03493	PF04263	PF05071	PF06008	PF07177	PF08170
PF00285	PF00682	PF01106	PF01531	PF02173	PF02836	PF03494	PF04265	PF05083	PF06009	PF07189	PF08180
PF00287	PF00683	PF01109	PF01532	PF02174	PF02837	PF03501	PF04272	PF05089	PF06010	PF07202	PF08185
PF00288	PF00684	PF01110	PF01534	PF02177	PF02838	PF03508	PF04275	PF05090	PF06012	PF07225	PF08188
PF00289	PF00685	PF01111	PF01536	PF02178	PF02840	PF03509	PF04280	PF05091	PF06017	PF07260	PF08189
PF00291	PF00686	PF01112	PF01545	PF02179	PF02841	PF03511	PF04300	PF05093	PF06025	PF07263	PF08198
PF00292	PF00687	PF01114	PF01549	PF02180	PF02843	PF03516	PF04376	PF05104	PF06026	PF07264	PF08202
PF00293	PF00689	PF01115	PF01551	PF02181	PF02844	PF03517	PF04377	PF05110	PF06031	PF07276	PF08204
PF00294	PF00690	PF01119	PF01553	PF02182	PF02845	PF03520	PF04379	PF05111	PF06046	PF07281	PF08210
PF00297	PF00692	PF01120	PF01556	PF02184	PF02847	PF03521	PF04382	PF05118	PF06049	PF07286	PF08212
PF00298	PF00694	PF01121	PF01557	PF02185	PF02852	PF03522	PF04388	PF05127	PF06052	PF07292	PF08213
PF00300	PF00696	PF01122	PF01562	PF02186	PF02854	PF03523	PF04389	PF05131	PF06058	PF07297	PF08215
PF00303	PF00698	PF01124	PF01564	PF02187	PF02862	PF03528	PF04406	PF05132	PF06060	PF07303	PF08216
PF00305	PF00704	PF01125	PF01565	PF02189	PF02864	PF03529	PF04408	PF05147	PF06068	PF07324	PF08221
PF00306	PF00705	PF01126	PF01566	PF02190	PF02865	PF03530	PF04410	PF05148	PF06071	PF07334	PF08225
PF00307	PF00707	PF01127	PF01569	PF02191	PF02866	PF03531	PF04418	PF05153	PF06079	PF07347	PF08232
PF00310	PF00708	PF01129	PF01571	PF02192	PF02867	PF03533	PF04419	PF05158	PF06083	PF07353	PF08235
PF00312	PF00709	PF01130	PF01575	PF02194	PF02870	PF03535	PF04420	PF05160	PF06087	PF07354	PF08240
PF00313	PF00710	PF01131	PF01582	PF02196	PF02872	PF03540	PF04421	PF05162	PF06093	PF07359	PF08241
PF00316	PF00711	PF01133	PF01583	PF02197	PF02874	PF03542	PF04423	PF05181	PF06105	PF07400	PF08242
PF00317	PF00714	PF01134	PF01585	PF02198	PF02875	PF03546	PF04427	PF05184	PF06119	PF07406	PF08246
PF00318	PF00715	PF01135	PF01586	PF02199	PF02877	PF03547	PF04433	PF05185	PF06121	PF07412	PF08248
PF00319	PF00717	PF01138	PF01588	PF02201	PF02879	PF03556	PF04434	PF05186	PF06140	PF07421	PF08256
PF00320	PF00719	PF01140	PF01590	PF02202	PF02880	PF03561	PF04437	PF05187	PF06148	PF07425	PF08264
PF00323	PF00727	PF01142	PF01591	PF02204	PF02881	PF03567	PF04438	PF05188	PF06179	PF07440	PF08265

PF00324	PF00728	PF01144	PF01592	PF02205	PF02882	PF03568	PF04440	PF05189	PF06189	PF07443	PF08266
PF00326	PF00731	PF01146	PF01596	PF02207	PF02883	PF03571	PF04442	PF05190	PF06201	PF07448	PF08271
PF00327	PF00732	PF01148	PF01597	PF02208	PF02885	PF03572	PF04487	PF05191	PF06202	PF07452	PF08279
PF00328	PF00733	PF01150	PF01598	PF02209	PF02887	PF03575	PF04488	PF05193	PF06209	PF07458	PF08285
PF00329	PF00735	PF01151	PF01599	PF02212	PF02888	PF03577	PF04494	PF05195	PF06211	PF07469	PF08288
PF00330	PF00736	PF01153	PF01602	PF02213	PF02889	PF03587	PF04495	PF05196	PF06212	PF07474	PF08292
PF00333	PF00743	PF01154	PF01603	PF02214	PF02891	PF03600	PF04503	PF05199	PF06214	PF07479	PF08295
PF00334	PF00748	PF01157	PF01608	PF02218	PF02892	PF03604	PF04505	PF05207	PF06218	PF07496	PF08311
PF00335	PF00749	PF01158	PF01619	PF02219	PF02893	PF03607	PF04511	PF05208	PF06220	PF07500	PF08313
PF00337	PF00750	PF01159	PF01624	PF02221	PF02897	PF03615	PF04515	PF05222	PF06221	PF07502	PF08318
PF00338	PF00751	PF01160	PF01625	PF02223	PF02898	PF03619	PF04538	PF05236	PF06229	PF07521	PF08321
PF00339	PF00752	PF01161	PF01630	PF02225	PF02902	PF03623	PF04547	PF05237	PF06237	PF07522	PF08326

**Metazoa + Fungi** Pfam ID														
PF00005	PF00398	PF00809	PF01187	PF01599	PF02140	PF02760	PF03260	PF03896	PF04500	PF05060	PF05781	PF06644	PF07711	PF08170
PF00006	PF00399	PF00810	PF01191	PF01602	PF02141	PF02761	PF03261	PF03900	PF04502	PF05063	PF05782	PF06645	PF07716	PF08171
PF00010	PF00401	PF00811	PF01192	PF01603	PF02144	PF02762	PF03265	PF03901	PF04503	PF05064	PF05783	PF06650	PF07717	PF08174
PF00013	PF00402	PF00812	PF01194	PF01608	PF02146	PF02765	PF03266	PF03902	PF04505	PF05071	PF05786	PF06652	PF07718	PF08180
PF00014	PF00403	PF00814	PF01195	PF01619	PF02148	PF02769	PF03271	PF03907	PF04506	PF05083	PF05790	PF06657	PF07719	PF08184
PF00017	PF00410	PF00815	PF01196	PF01625	PF02149	PF02770	PF03285	PF03908	PF04511	PF05089	PF05793	PF06658	PF07720	PF08185
PF00019	PF00411	PF00817	PF01199	PF01627	PF02150	PF02771	PF03291	PF03909	PF04515	PF05090	PF05794	PF06662	PF07722	PF08187
PF00025	PF00412	PF00819	PF01200	PF01630	PF02152	PF02772	PF03297	PF03911	PF04516	PF05091	PF05804	PF06663	PF07723	PF08188
PF00026	PF00414	PF00821	PF01201	PF01634	PF02156	PF02773	PF03299	PF03914	PF04538	PF05093	PF05805	PF06668	PF07724	PF08189
PF00029	PF00416	PF00822	PF01202	PF01642	PF02157	PF02774	PF03301	PF03915	PF04547	PF05104	PF05808	PF06677	PF07731	PF08192
PF00030	PF00418	PF00826	PF01204	PF01644	PF02162	PF02779	PF03308	PF03919	PF04548	PF05110	PF05811	PF06687	PF07732	PF08194
PF00032	PF00420	PF00827	PF01205	PF01645	PF02165	PF02780	PF03311	PF03921	PF04549	PF05111	PF05817	PF06699	PF07735	PF08198
PF00034	PF00425	PF00828	PF01207	PF01648	PF02167	PF02781	PF03321	PF03928	PF04553	PF05118	PF05821	PF06701	PF07738	PF08202
PF00037	PF00428	PF00829	PF01208	PF01652	PF02170	PF02784	PF03332	PF03931	PF04557	PF05127	PF05822	PF06702	PF07740	PF08203
PF00039	PF00433	PF00830	PF01209	PF01655	PF02171	PF02785	PF03344	PF03932	PF04558	PF05131	PF05823	PF06703	PF07741	PF08204
PF00040	PF00436	PF00831	PF01210	PF01658	PF02172	PF02789	PF03345	PF03935	PF04560	PF05132	PF05824	PF06726	PF07742	PF08206
PF00044	PF00438	PF00832	PF01212	PF01661	PF02173	PF02791	PF03348	PF03937	PF04561	PF05141	PF05826	PF06728	PF07743	PF08208
PF00045	PF00439	PF00833	PF01213	PF01663	PF02174	PF02792	PF03351	PF03939	PF04565	PF05147	PF05827	PF06729	PF07744	PF08210
PF00048	PF00444	PF00834	PF01214	PF01667	PF02177	PF02793	PF03359	PF03940	PF04566	PF05148	PF05832	PF06732	PF07745	PF08212
PF00050	PF00445	PF00835	PF01215	PF01669	PF02178	PF02795	PF03360	PF03941	PF04567	PF05151	PF05835	PF06733	PF07748	PF08213
PF00051	PF00446	PF00836	PF01216	PF01670	PF02179	PF02799	PF03366	PF03943	PF04568	PF05153	PF05836	PF06740	PF07749	PF08215
PF00052	PF00447	PF00837	PF01217	PF01679	PF02180	PF02800	PF03367	PF03946	PF04571	PF05154	PF05839	PF06741	PF07763	PF08216
PF00054	PF00448	PF00839	PF01218	PF01680	PF02181	PF02803	PF03368	PF03947	PF04572	PF05156	PF05841	PF06743	PF07766	PF08217
PF00055	PF00449	PF00840	PF01220	PF01681	PF02182	PF02804	PF03370	PF03949	PF04573	PF05158	PF05843	PF06752	PF07767	PF08221
PF00056	PF00450	PF00842	PF01221	PF01683	PF02184	PF02806	PF03371	PF03950	PF04574	PF05160	PF05849	PF06753	PF07770	PF08225
PF00058	PF00451	PF00849	PF01223	PF01687	PF02185	PF02807	PF03372	PF03952	PF04579	PF05161	PF05856	PF06756	PF07774	PF08226
PF00060	PF00452	PF00850	PF01226	PF01694	PF02186	PF02809	PF03378	PF03954	PF04587	PF05162	PF05859	PF06757	PF07776	PF08227
PF00062	PF00454	PF00852	PF01227	PF01697	PF02187	PF02811	PF03381	PF03957	PF04588	PF05172	PF05863	PF06766	PF07778	PF08228
PF00066	PF00457	PF00853	PF01229	PF01699	PF02189	PF02812	PF03388	PF03959	PF04589	PF05176	PF05871	PF06773	PF07780	PF08229

PF00068	PF00458	PF00855	PF01230	PF01702	PF02190	PF02815	PF03392	PF03964	PF04590	PF05178	PF05875	PF06775	PF07782	PF08232
PF00072	PF00459	PF00856	PF01231	PF01704	PF02191	PF02816	PF03398	PF03966	PF04592	PF05180	PF05879	PF06777	PF07798	PF08234
PF00074	PF00462	PF00857	PF01232	PF01705	PF02192	PF02817	PF03399	PF03969	PF04593	PF05181	PF05881	PF06783	PF07807	PF08235
PF00075	PF00463	PF00860	PF01233	PF01709	PF02194	PF02819	PF03402	PF03972	PF04597	PF05182	PF05889	PF06784	PF07808	PF08236
PF00077	PF00464	PF00861	PF01234	PF01712	PF02195	PF02820	PF03403	PF03973	PF04598	PF05183	PF05890	PF06807	PF07810	PF08240
PF00080	PF00465	PF00864	PF01237	PF01713	PF02196	PF02822	PF03404	PF03980	PF04603	PF05184	PF05891	PF06814	PF07815	PF08242
PF00081	PF00466	PF00865	PF01238	PF01715	PF02198	PF02823	PF03414	PF03981	PF04611	PF05185	PF05902	PF06816	PF07817	PF08243
PF00085	PF00467	PF00867	PF01239	PF01717	PF02199	PF02824	PF03416	PF03982	PF04614	PF05186	PF05903	PF06817	PF07818	PF08244
PF00087	PF00468	PF00868	PF01242	PF01722	PF02200	PF02825	PF03417	PF03983	PF04615	PF05187	PF05912	PF06818	PF07819	PF08245
PF00088	PF00472	PF00870	PF01243	PF01723	PF02201	PF02826	PF03422	PF03985	PF04617	PF05188	PF05916	PF06821	PF07822	PF08246
PF00090	PF00473	PF00875	PF01244	PF01725	PF02202	PF02827	PF03437	PF03986	PF04621	PF05189	PF05918	PF06824	PF07829	PF08248
PF00091	PF00474	PF00876	PF01245	PF01728	PF02204	PF02828	PF03439	PF03987	PF04622	PF05190	PF05922	PF06825	PF07830	PF08249
PF00093	PF00475	PF00879	PF01246	PF01729	PF02205	PF02833	PF03441	PF03990	PF04625	PF05191	PF05923	PF06827	PF07831	PF08250
PF00095	PF00476	PF00881	PF01247	PF01731	PF02206	PF02836	PF03443	PF03991	PF04626	PF05195	PF05924	PF06828	PF07834	PF08251
PF00096	PF00479	PF00884	PF01248	PF01733	PF02207	PF02840	PF03446	PF03992	PF04627	PF05196	PF05933	PF06831	PF07837	PF08252
PF00098	PF00480	PF00885	PF01249	PF01734	PF02208	PF02841	PF03447	PF03997	PF04628	PF05199	PF05937	PF06839	PF07842	PF08256
PF00107	PF00481	PF00886	PF01251	PF01740	PF02209	PF02843	PF03450	PF03998	PF04629	PF05202	PF05956	PF06858	PF07847	PF08257
PF00108	PF00484	PF00887	PF01253	PF01743	PF02212	PF02844	PF03451	PF03999	PF04636	PF05204	PF05958	PF06862	PF07850	PF08258
PF00112	PF00485	PF00889	PF01254	PF01746	PF02213	PF02845	PF03452	PF04001	PF04641	PF05205	PF05964	PF06870	PF07851	PF08259
PF00113	PF00487	PF00890	PF01255	PF01747	PF02218	PF02847	PF03453	PF04003	PF04643	PF05207	PF05965	PF06875	PF07856	PF08260
PF00116	PF00488	PF00891	PF01256	PF01748	PF02219	PF02852	PF03454	PF04004	PF04648	PF05208	PF05967	PF06881	PF07857	PF08261
PF00117	PF00489	PF00892	PF01257	PF01749	PF02221	PF02861	PF03455	PF04005	PF04652	PF05216	PF05971	PF06886	PF07859	PF08262
PF00121	PF00490	PF00895	PF01259	PF01751	PF02222	PF02862	PF03456	PF04006	PF04658	PF05222	PF05972	PF06888	PF07883	PF08265
PF00123	PF00491	PF00896	PF01261	PF01753	PF02223	PF02864	PF03460	PF04030	PF04664	PF05224	PF05978	PF06905	PF07884	PF08266
PF00129	PF00493	PF00899	PF01263	PF01754	PF02225	PF02865	PF03462	PF04031	PF04667	PF05225	PF05980	PF06907	PF07885	PF08267
PF00130	PF00494	PF00900	PF01264	PF01756	PF02229	PF02866	PF03463	PF04032	PF04668	PF05234	PF05995	PF06910	PF07894	PF08271
PF00131	PF00498	PF00903	PF01265	PF01757	PF02230	PF02867	PF03464	PF04034	PF04670	PF05236	PF05997	PF06916	PF07896	PF08279
PF00132	PF00499	PF00904	PF01267	PF01758	PF02233	PF02870	PF03465	PF04037	PF04675	PF05237	PF06001	PF06920	PF07904	PF08285
PF00136	PF00507	PF00909	PF01268	PF01759	PF02234	PF02872	PF03467	PF04042	PF04676	PF05238	PF06008	PF06936	PF07912	PF08286
PF00137	PF00510	PF00916	PF01269	PF01761	PF02237	PF02874	PF03477	PF04044	PF04677	PF05240	PF06009	PF06937	PF07915	PF08287
PF00141	PF00512	PF00917	PF01271	PF01762	PF02238	PF02875	PF03481	PF04045	PF04679	PF05241	PF06010	PF06941	PF07923	PF08288
PF00144	PF00521	PF00919	PF01273	PF01765	PF02244	PF02877	PF03483	PF04046	PF04680	PF05242	PF06011	PF06951	PF07926	PF08292

PF00145	PF00525	PF00920	PF01274	PF01770	PF02245	PF02879	PF03484	PF04047	PF04683	PF05250	PF06012	PF06954	PF07928	PF08293
PF00146	PF00531	PF00923	PF01275	PF01773	PF02251	PF02880	PF03485	PF04048	PF04691	PF05251	PF06017	PF06957	PF07933	PF08295
PF00152	PF00538	PF00925	PF01279	PF01775	PF02252	PF02881	PF03487	PF04049	PF04692	PF05253	PF06020	PF06959	PF07934	PF08296
PF00153	PF00542	PF00926	PF01280	PF01776	PF02257	PF02882	PF03489	PF04050	PF04695	PF05254	PF06025	PF06963	PF07936	PF08297
PF00157	PF00544	PF00928	PF01281	PF01777	PF02259	PF02883	PF03491	PF04051	PF04696	PF05255	PF06026	PF06964	PF07938	PF08302
PF00159	PF00545	PF00929	PF01282	PF01778	PF02260	PF02885	PF03493	PF04053	PF04697	PF05270	PF06031	PF06968	PF07941	PF08303
PF00160	PF00547	PF00930	PF01283	PF01779	PF02262	PF02887	PF03494	PF04054	PF04698	PF05276	PF06046	PF06970	PF07942	PF08304
PF00162	PF00549	PF00931	PF01284	PF01780	PF02265	PF02888	PF03501	PF04055	PF04699	PF05277	PF06049	PF06978	PF07945	PF08311
PF00163	PF00551	PF00932	PF01285	PF01781	PF02267	PF02889	PF03508	PF04056	PF04704	PF05279	PF06052	PF06984	PF07946	PF08312
PF00166	PF00552	PF00933	PF01286	PF01783	PF02268	PF02891	PF03509	PF04057	PF04706	PF05281	PF06058	PF06985	PF07954	PF08313
PF00171	PF00554	PF00935	PF01287	PF01784	PF02269	PF02892	PF03511	PF04059	PF04707	PF05282	PF06060	PF06990	PF07955	PF08315
PF00172	PF00560	PF00940	PF01288	PF01786	PF02270	PF02894	PF03516	PF04061	PF04709	PF05283	PF06068	PF06991	PF07956	PF08316
PF00173	PF00562	PF00941	PF01290	PF01791	PF02271	PF02897	PF03517	PF04062	PF04710	PF05285	PF06071	PF06994	PF07957	PF08317
PF00174	PF00564	PF00953	PF01291	PF01793	PF02272	PF02898	PF03520	PF04063	PF04711	PF05287	PF06079	PF07001	PF07958	PF08318
PF00177	PF00566	PF00956	PF01293	PF01794	PF02274	PF02902	PF03521	PF04064	PF04714	PF05291	PF06083	PF07002	PF07959	PF08319
PF00178	PF00568	PF00958	PF01300	PF01798	PF02275	PF02906	PF03522	PF04065	PF04715	PF05292	PF06087	PF07010	PF07960	PF08320
PF00179	PF00569	PF00961	PF01302	PF01799	PF02284	PF02910	PF03523	PF04068	PF04716	PF05296	PF06093	PF07019	PF07961	PF08321
PF00180	PF00570	PF00962	PF01303	PF01803	PF02285	PF02911	PF03525	PF04072	PF04718	PF05298	PF06105	PF07034	PF07962	PF08322
PF00181	PF00572	PF00969	PF01305	PF01805	PF02290	PF02913	PF03528	PF04078	PF04719	PF05300	PF06119	PF07035	PF07964	PF08325
PF00184	PF00573	PF00975	PF01315	PF01808	PF02291	PF02919	PF03529	PF04080	PF04722	PF05303	PF06121	PF07054	PF07965	PF08326
PF00185	PF00574	PF00977	PF01323	PF01812	PF02292	PF02920	PF03530	PF04081	PF04724	PF05316	PF06127	PF07061	PF07966	PF08327
PF00186	PF00575	PF00984	PF01327	PF01813	PF02296	PF02921	PF03531	PF04083	PF04727	PF05324	PF06140	PF07062	PF07967	PF08332
PF00187	PF00576	PF00988	PF01328	PF01814	PF02297	PF02922	PF03533	PF04084	PF04729	PF05326	PF06148	PF07064	PF07970	PF08333
PF00188	PF00578	PF00993	PF01329	PF01817	PF02301	PF02928	PF03535	PF04086	PF04731	PF05327	PF06151	PF07074	PF07973	PF08336
PF00189	PF00579	PF00994	PF01331	PF01822	PF02310	PF02929	PF03540	PF04088	PF04732	PF05328	PF06172	PF07081	PF07974	PF08337
PF00191	PF00580	PF00996	PF01335	PF01823	PF02312	PF02933	PF03542	PF04089	PF04733	PF05337	PF06179	PF07084	PF07975	PF08344
PF00193	PF00581	PF00999	PF01336	PF01827	PF02318	PF02934	PF03547	PF04090	PF04736	PF05345	PF06189	PF07093	PF07976	PF08351
PF00198	PF00582	PF01000	PF01341	PF01828	PF02319	PF02935	PF03556	PF04091	PF04739	PF05346	PF06201	PF07106	PF07978	PF08354
PF00199	PF00584	PF01007	PF01342	PF01833	PF02320	PF02937	PF03561	PF04095	PF04749	PF05347	PF06202	PF07111	PF07985	PF08355
PF00202	PF00585	PF01008	PF01347	PF01835	PF02330	PF02939	PF03567	PF04096	PF04750	PF05348	PF06209	PF07140	PF07986	PF08356
PF00203	PF00587	PF01011	PF01348	PF01839	PF02337	PF02940	PF03568	PF04097	PF04752	PF05349	PF06211	PF07142	PF07988	PF08357
PF00204	PF00588	PF01012	PF01351	PF01840	PF02338	PF02944	PF03571	PF04098	PF04756	PF05350	PF06212	PF07145	PF07989	PF08365

PF00206	PF00590	PF01014	PF01353	PF01841	PF02344	PF02946	PF03572	PF04099	PF04757	PF05351	PF06214	PF07147	PF07991	PF08366
PF00207	PF00591	PF01015	PF01354	PF01842	PF02345	PF02947	PF03575	PF04100	PF04758	PF05353	PF06218	PF07149	PF07993	PF08367
PF00208	PF00594	PF01016	PF01363	PF01843	PF02347	PF02948	PF03577	PF04101	PF04760	PF05355	PF06220	PF07156	PF07994	PF08368
PF00209	PF00596	PF01017	PF01365	PF01844	PF02348	PF02949	PF03583	PF04103	PF04762	PF05362	PF06221	PF07159	PF08005	PF08373
PF00210	PF00605	PF01018	PF01368	PF01846	PF02350	PF02961	PF03587	PF04104	PF04768	PF05365	PF06229	PF07163	PF08015	PF08374
PF00211	PF00609	PF01020	PF01369	PF01847	PF02351	PF02965	PF03595	PF04106	PF04774	PF05366	PF06237	PF07165	PF08016	PF08375
PF00213	PF00610	PF01021	PF01372	PF01852	PF02354	PF02966	PF03600	PF04109	PF04775	PF05374	PF06248	PF07169	PF08022	PF08377
PF00214	PF00612	PF01023	PF01379	PF01853	PF02358	PF02969	PF03604	PF04110	PF04777	PF05378	PF06268	PF07175	PF08023	PF08383
PF00215	PF00613	PF01025	PF01380	PF01857	PF02359	PF02970	PF03615	PF04111	PF04790	PF05383	PF06292	PF07177	PF08024	PF08384
PF00218	PF00614	PF01026	PF01382	PF01858	PF02364	PF02971	PF03619	PF04112	PF04791	PF05386	PF06294	PF07200	PF08025	PF08385
PF00219	PF00616	PF01027	PF01384	PF01866	PF02366	PF02973	PF03623	PF04113	PF04795	PF05387	PF06297	PF07202	PF08026	PF08389
PF00220	PF00617	PF01028	PF01387	PF01868	PF02372	PF02978	PF03630	PF04114	PF04799	PF05388	PF06309	PF07225	PF08029	PF08390
PF00221	PF00618	PF01033	PF01388	PF01869	PF02374	PF02982	PF03632	PF04115	PF04800	PF05390	PF06311	PF07228	PF08030	PF08393
PF00224	PF00621	PF01035	PF01390	PF01872	PF02376	PF02985	PF03633	PF04116	PF04801	PF05391	PF06312	PF07247	PF08032	PF08397
PF00225	PF00623	PF01036	PF01392	PF01873	PF02377	PF02987	PF03635	PF04117	PF04802	PF05392	PF06313	PF07249	PF08033	PF08399
PF00230	PF00624	PF01039	PF01393	PF01875	PF02383	PF02990	PF03636	PF04118	PF04803	PF05395	PF06314	PF07253	PF08034	PF08403
PF00231	PF00627	PF01040	PF01394	PF01876	PF02386	PF02991	PF03637	PF04119	PF04810	PF05397	PF06320	PF07260	PF08035	PF08407
PF00232	PF00629	PF01042	PF01395	PF01883	PF02390	PF02994	PF03639	PF04120	PF04811	PF05399	PF06324	PF07263	PF08036	PF08412
PF00235	PF00632	PF01048	PF01396	PF01885	PF02391	PF02996	PF03641	PF04121	PF04812	PF05404	PF06328	PF07264	PF08037	PF08416
PF00236	PF00635	PF01055	PF01397	PF01894	PF02403	PF03002	PF03643	PF04124	PF04813	PF05405	PF06330	PF07276	PF08038	PF08418
PF00237	PF00636	PF01058	PF01398	PF01896	PF02404	PF03006	PF03645	PF04127	PF04814	PF05406	PF06331	PF07281	PF08039	PF08420
PF00238	PF00638	PF01059	PF01401	PF01900	PF02410	PF03016	PF03647	PF04128	PF04815	PF05418	PF06333	PF07286	PF08040	PF08424
PF00241	PF00639	PF01060	PF01403	PF01907	PF02412	PF03020	PF03648	PF04129	PF04818	PF05427	PF06337	PF07292	PF08045	PF08426
PF00243	PF00642	PF01061	PF01404	PF01910	PF02422	PF03024	PF03651	PF04130	PF04819	PF05428	PF06342	PF07294	PF08058	PF08429
PF00244	PF00643	PF01063	PF01410	PF01912	PF02423	PF03027	PF03657	PF04132	PF04821	PF05433	PF06345	PF07303	PF08059	PF08430
PF00245	PF00644	PF01064	PF01411	PF01915	PF02425	PF03029	PF03659	PF04133	PF04822	PF05434	PF06350	PF07324	PF08060	PF08432
PF00246	PF00645	PF01066	PF01412	PF01916	PF02434	PF03031	PF03660	PF04135	PF04824	PF05436	PF06357	PF07327	PF08061	PF08434
PF00248	PF00646	PF01067	PF01413	PF01918	PF02436	PF03032	PF03661	PF04136	PF04825	PF05438	PF06365	PF07334	PF08062	PF08438
PF00250	PF00647	PF01068	PF01414	PF01922	PF02437	PF03033	PF03663	PF04137	PF04831	PF05439	PF06367	PF07347	PF08063	PF08441
PF00251	PF00649	PF01070	PF01415	PF01923	PF02441	PF03034	PF03664	PF04139	PF04836	PF05450	PF06369	PF07353	PF08064	PF08442
PF00252	PF00653	PF01071	PF01416	PF01926	PF02450	PF03036	PF03665	PF04140	PF04840	PF05453	PF06372	PF07354	PF08065	PF08448
PF00253	PF00654	PF01073	PF01417	PF01928	PF02453	PF03039	PF03666	PF04144	PF04841	PF05454	PF06373	PF07359	PF08066	PF08449

PF00255	PF00656	PF01074	PF01419	PF01929	PF02460	PF03045	PF03669	PF04145	PF04845	PF05456	PF06374	PF07365	PF08067	PF08451
PF00256	PF00657	PF01077	PF01421	PF01933	PF02466	PF03051	PF03670	PF04146	PF04847	PF05458	PF06375	PF07366	PF08068	PF08454
PF00258	PF00658	PF01079	PF01422	PF01937	PF02469	PF03055	PF03671	PF04147	PF04855	PF05460	PF06384	PF07367	PF08069	PF08466
PF00260	PF00659	PF01080	PF01424	PF01938	PF02485	PF03056	PF03674	PF04148	PF04856	PF05461	PF06387	PF07393	PF08070	PF08473
PF00262	PF00662	PF01082	PF01425	PF01946	PF02487	PF03061	PF03676	PF04152	PF04858	PF05463	PF06391	PF07400	PF08071	PF08474
PF00265	PF00666	PF01083	PF01426	PF01958	PF02492	PF03062	PF03690	PF04153	PF04868	PF05466	PF06393	PF07406	PF08072	PF08477
PF00268	PF00670	PF01084	PF01428	PF01960	PF02493	PF03068	PF03694	PF04157	PF04869	PF05469	PF06394	PF07412	PF08073	PF08487
PF00272	PF00673	PF01085	PF01432	PF01963	PF02494	PF03070	PF03700	PF04158	PF04871	PF05470	PF06395	PF07421	PF08074	PF08490
PF00273	PF00676	PF01086	PF01434	PF01965	PF02513	PF03073	PF03712	PF04163	PF04874	PF05476	PF06396	PF07425	PF08075	PF08491
PF00274	PF00677	PF01087	PF01435	PF01966	PF02515	PF03074	PF03715	PF04176	PF04882	PF05477	PF06398	PF07440	PF08079	PF08492
PF00275	PF00679	PF01088	PF01436	PF01967	PF02516	PF03081	PF03718	PF04177	PF04889	PF05478	PF06399	PF07442	PF08080	PF08493
PF00278	PF00683	PF01090	PF01437	PF01968	PF02517	PF03082	PF03719	PF04178	PF04892	PF05482	PF06400	PF07443	PF08081	PF08498
PF00280	PF00684	PF01091	PF01439	PF01974	PF02520	PF03088	PF03720	PF04179	PF04893	PF05483	PF06401	PF07448	PF08082	PF08499
PF00281	PF00685	PF01093	PF01448	PF01975	PF02525	PF03089	PF03721	PF04180	PF04898	PF05485	PF06403	PF07452	PF08083	PF08501
PF00285	PF00686	PF01095	PF01450	PF01981	PF02535	PF03091	PF03722	PF04181	PF04900	PF05486	PF06417	PF07458	PF08084	PF08502
PF00287	PF00687	PF01096	PF01451	PF01984	PF02536	PF03095	PF03723	PF04182	PF04901	PF05492	PF06418	PF07464	PF08086	PF08504
PF00288	PF00689	PF01101	PF01459	PF01988	PF02538	PF03096	PF03725	PF04185	PF04902	PF05493	PF06419	PF07469	PF08088	PF08506
PF00290	PF00690	PF01103	PF01462	PF01991	PF02541	PF03097	PF03726	PF04188	PF04904	PF05495	PF06420	PF07473	PF08089	PF08508
PF00291	PF00692	PF01105	PF01463	PF01992	PF02544	PF03099	PF03727	PF04189	PF04905	PF05499	PF06421	PF07474	PF08091	PF08509
PF00292	PF00694	PF01106	PF01464	PF01997	PF02545	PF03102	PF03730	PF04191	PF04908	PF05502	PF06423	PF07477	PF08092	PF08510
PF00293	PF00696	PF01109	PF01465	PF02005	PF02548	PF03104	PF03731	PF04192	PF04909	PF05507	PF06424	PF07479	PF08093	PF08513
PF00297	PF00699	PF01110	PF01466	PF02008	PF02550	PF03105	PF03733	PF04193	PF04910	PF05508	PF06425	PF07488	PF08094	PF08514
PF00298	PF00706	PF01111	PF01467	PF02012	PF02551	PF03109	PF03735	PF04194	PF04911	PF05510	PF06427	PF07491	PF08095	PF08515
PF00300	PF00707	PF01112	PF01469	PF02013	PF02567	PF03124	PF03736	PF04201	PF04912	PF05517	PF06428	PF07492	PF08096	PF08516
PF00303	PF00708	PF01114	PF01470	PF02014	PF02569	PF03126	PF03737	PF04209	PF04921	PF05522	PF06432	PF07493	PF08097	PF08517
PF00304	PF00709	PF01115	PF01472	PF02015	PF02578	PF03127	PF03746	PF04212	PF04923	PF05529	PF06441	PF07496	PF08098	PF08518
PF00305	PF00710	PF01116	PF01474	PF02017	PF02580	PF03128	PF03747	PF04218	PF04925	PF05546	PF06446	PF07500	PF08099	PF08519
PF00306	PF00711	PF01118	PF01477	PF02019	PF02581	PF03129	PF03758	PF04230	PF04926	PF05571	PF06448	PF07502	PF08101	PF08520
PF00307	PF00713	PF01119	PF01479	PF02020	PF02582	PF03130	PF03762	PF04253	PF04930	PF05572	PF06451	PF07519	PF08102	PF08523
PF00313	PF00714	PF01120	PF01480	PF02022	PF02585	PF03131	PF03765	PF04258	PF04934	PF05577	PF06456	PF07521	PF08103	PF08526
PF00317	PF00715	PF01121	PF01482	PF02025	PF02586	PF03133	PF03768	PF04272	PF04935	PF05586	PF06457	PF07522	PF08104	PF08527
PF00318	PF00717	PF01122	PF01483	PF02026	PF02594	PF03134	PF03769	PF04275	PF04938	PF05587	PF06459	PF07524	PF08105	PF08528

PF00319	PF00719	PF01124	PF01484	PF02035	PF02597	PF03145	PF03770	PF04280	PF04939	PF05600	PF06461	PF07525	PF08106	PF08534
PF00320	PF00723	PF01125	PF01485	PF02036	PF02598	PF03146	PF03778	PF04281	PF04950	PF05604	PF06462	PF07527	PF08108	PF08537
PF00323	PF00724	PF01126	PF01487	PF02037	PF02602	PF03147	PF03781	PF04299	PF04952	PF05609	PF06463	PF07528	PF08110	PF08540
PF00324	PF00727	PF01127	PF01491	PF02039	PF02607	PF03148	PF03782	PF04300	PF04959	PF05622	PF06464	PF07529	PF08111	PF08542
PF00327	PF00728	PF01129	PF01493	PF02044	PF02617	PF03149	PF03792	PF04318	PF04960	PF05637	PF06465	PF07530	PF08114	PF08544
PF00328	PF00731	PF01130	PF01496	PF02045	PF02626	PF03151	PF03798	PF04324	PF04961	PF05640	PF06466	PF07531	PF08115	PF08546
PF00329	PF00733	PF01131	PF01498	PF02046	PF02627	PF03152	PF03800	PF04325	PF04968	PF05641	PF06467	PF07533	PF08116	
PF00330	PF00734	PF01133	PF01502	PF02058	PF02628	PF03153	PF03801	PF04366	PF04969	PF05645	PF06468	PF07535	PF08117	
PF00331	PF00735	PF01134	PF01503	PF02059	PF02629	PF03155	PF03803	PF04370	PF04970	PF05648	PF06469	PF07539	PF08119	
PF00333	PF00736	PF01135	PF01504	PF02064	PF02630	PF03159	PF03807	PF04376	PF04979	PF05649	PF06470	PF07540	PF08120	
PF00334	PF00742	PF01139	PF01505	PF02066	PF02636	PF03160	PF03810	PF04377	PF04981	PF05653	PF06472	PF07541	PF08121	
PF00335	PF00743	PF01140	PF01507	PF02067	PF02637	PF03161	PF03813	PF04379	PF04983	PF05669	PF06479	PF07542	PF08122	
PF00338	PF00748	PF01142	PF01509	PF02071	PF02656	PF03164	PF03815	PF04382	PF04987	PF05670	PF06480	PF07543	PF08123	
PF00342	PF00749	PF01144	PF01512	PF02076	PF02666	PF03165	PF03820	PF04387	PF04988	PF05671	PF06482	PF07545	PF08125	
PF00343	PF00751	PF01146	PF01513	PF02077	PF02671	PF03166	PF03821	PF04388	PF04990	PF05676	PF06487	PF07546	PF08127	
PF00344	PF00752	PF01148	PF01521	PF02078	PF02676	PF03167	PF03823	PF04406	PF04992	PF05679	PF06495	PF07557	PF08131	
PF00346	PF00755	PF01150	PF01522	PF02079	PF02678	PF03169	PF03824	PF04408	PF05000	PF05680	PF06512	PF07558	PF08138	
PF00347	PF00756	PF01151	PF01529	PF02083	PF02682	PF03171	PF03826	PF04410	PF05001	PF05682	PF06519	PF07562	PF08140	
PF00348	PF00758	PF01153	PF01530	PF02084	PF02688	PF03172	PF03827	PF04418	PF05002	PF05693	PF06524	PF07565	PF08142	
PF00350	PF00762	PF01154	PF01531	PF02087	PF02696	PF03177	PF03828	PF04419	PF05004	PF05700	PF06534	PF07569	PF08143	
PF00351	PF00763	PF01156	PF01532	PF02088	PF02705	PF03178	PF03832	PF04420	PF05005	PF05706	PF06535	PF07571	PF08144	
PF00352	PF00764	PF01157	PF01534	PF02089	PF02709	PF03179	PF03834	PF04421	PF05007	PF05712	PF06540	PF07572	PF08145	
PF00354	PF00766	PF01158	PF01536	PF02093	PF02714	PF03184	PF03835	PF04423	PF05010	PF05715	PF06544	PF07573	PF08146	
PF00355	PF00769	PF01159	PF01541	PF02096	PF02724	PF03185	PF03836	PF04424	PF05014	PF05719	PF06546	PF07575	PF08147	
PF00357	PF00773	PF01160	PF01544	PF02098	PF02728	PF03188	PF03839	PF04425	PF05019	PF05721	PF06553	PF07576	PF08148	
PF00362	PF00774	PF01161	PF01545	PF02100	PF02729	PF03190	PF03847	PF04426	PF05020	PF05722	PF06554	PF07593	PF08149	
PF00364	PF00777	PF01162	PF01551	PF02101	PF02731	PF03194	PF03849	PF04427	PF05021	PF05724	PF06566	PF07647	PF08150	
PF00365	PF00778	PF01163	PF01554	PF02102	PF02732	PF03198	PF03850	PF04428	PF05022	PF05726	PF06567	PF07649	PF08151	
PF00366	PF00779	PF01166	PF01557	PF02104	PF02733	PF03199	PF03851	PF04433	PF05023	PF05729	PF06573	PF07650	PF08152	
PF00368	PF00780	PF01167	PF01562	PF02106	PF02734	PF03200	PF03853	PF04434	PF05024	PF05730	PF06581	PF07651	PF08153	
PF00372	PF00784	PF01168	PF01564	PF02109	PF02735	PF03208	PF03855	PF04437	PF05026	PF05731	PF06583	PF07657	PF08154	
PF00375	PF00786	PF01169	PF01565	PF02110	PF02737	PF03215	PF03856	PF04438	PF05028	PF05735	PF06584	PF07662	PF08155	

PF00377	PF00787	PF01170	PF01569	PF02112	PF02738	PF03224	PF03858	PF04440	PF05030	PF05741	PF06588	PF07670	PF08156	
PF00378	PF00790	PF01171	PF01575	PF02114	PF02740	PF03226	PF03867	PF04442	PF05032	PF05742	PF06602	PF07677	PF08157	
PF00382	PF00791	PF01172	PF01581	PF02115	PF02743	PF03227	PF03868	PF04446	PF05033	PF05743	PF06608	PF07678	PF08158	
PF00383	PF00792	PF01174	PF01583	PF02116	PF02744	PF03232	PF03870	PF04468	PF05038	PF05746	PF06614	PF07683	PF08159	
PF00384	PF00793	PF01175	PF01586	PF02121	PF02747	PF03234	PF03871	PF04478	PF05039	PF05753	PF06621	PF07684	PF08160	
PF00386	PF00795	PF01176	PF01588	PF02126	PF02749	PF03239	PF03874	PF04479	PF05040	PF05756	PF06623	PF07687	PF08161	
PF00388	PF00797	PF01177	PF01591	PF02127	PF02750	PF03247	PF03875	PF04487	PF05041	PF05760	PF06625	PF07691	PF08162	
PF00390	PF00800	PF01179	PF01592	PF02130	PF02751	PF03248	PF03876	PF04488	PF05046	PF05761	PF06628	PF07699	PF08163	
PF00393	PF00801	PF01180	PF01595	PF02133	PF02755	PF03250	PF03878	PF04493	PF05047	PF05764	PF06631	PF07701	PF08164	
PF00394	PF00804	PF01183	PF01596	PF02134	PF02757	PF03253	PF03879	PF04494	PF05051	PF05769	PF06632	PF07703	PF08165	
PF00396	PF00806	PF01184	PF01597	PF02135	PF02758	PF03256	PF03893	PF04495	PF05053	PF05777	PF06637	PF07706	PF08168	
PF00397	PF00808	PF01185	PF01598	PF02137	PF02759	PF03259	PF03894	PF04499	PF05057	PF05778	PF06638	PF07710	PF08169	
